# Herbivory increases diversification across insect clades

**DOI:** 10.1038/ncomms9370

**Published:** 2015-09-24

**Authors:** John J. Wiens, Richard T. Lapoint, Noah K. Whiteman

**Affiliations:** 1 Department of Ecology and Evolutionary Biology, University of Arizona, 1041 E. Lowell Street, BioSciences West 310, Tucson, Arizona 85721, USA

## Abstract

Insects contain more than half of all living species, but the causes of their remarkable diversity remain poorly understood. Many authors have suggested that herbivory has accelerated diversification in many insect clades. However, others have questioned the role of herbivory in insect diversification. Here, we test the relationships between herbivory and insect diversification across multiple scales. We find a strong, positive relationship between herbivory and diversification among insect orders. However, herbivory explains less variation in diversification within some orders (Diptera, Hemiptera) or shows no significant relationship with diversification in others (Coleoptera, Hymenoptera, Orthoptera). Thus, we support the overall importance of herbivory for insect diversification, but also show that its impacts can vary across scales and clades. In summary, our results illuminate the causes of species richness patterns in a group containing most living species, and show the importance of ecological impacts on diversification in explaining the diversity of life.

## Abstract (web summary)

Insects include most living species, yet the causes of this remarkable diversity remain unclear. Here, the authors show a positive relationship between herbivory and diversification among insect orders, which suggests that herbivory helps explain insect diversity.

## Graphical abstract

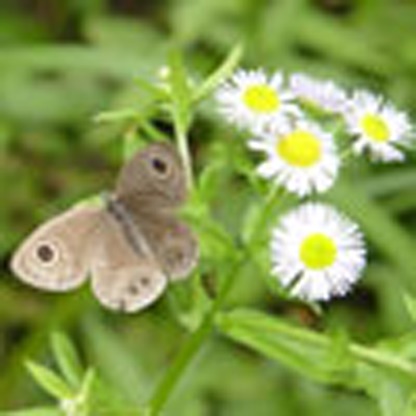

Insects contain ∼62% of all ∼1.6 million described living species^1^, but the causes of the exceptional diversity of this relatively recent branch (∼500 million years old) on the Tree of Life remain highly uncertain. Many authors have suggested that herbivory is a major driver of insect diversification^2^,^3^,^4^,^5^,^6^,^7^,^8^, especially feeding on living tissues of vascular plants (although some have also questioned its importance^9^,^10^). Thus, a strong relationship between herbivory and insect diversification, if supported, may be key to understanding the overall biodiversity of life on the Earth. The increasing availability of insect phylogenies (both within and between orders^10^,^11^,^12^,^13^) provides exciting new opportunities to test this relationship.

Given the potential importance of the herbivory-diversification relationship in insects, quantitative tests have been surprisingly few. In their classic study, Mitter *et al.*^5^ compared the species richness of 13 predominantly herbivorous clades with their non-herbivorous sister groups. Most comparisons were between sister clades (for example, families) within orders, including beetles. They found a significant positive impact of herbivory on diversification, based on the higher richness of the predominantly herbivorous clade in most sister-clade comparisons. Other studies have analysed the impact of transitions to phytophagy on diversification rates within beetles (Coleoptera; the most species-rich order^14^). These studies used a similar approach (comparing richness of sister clades). The first supported the role of herbivory in driving beetle diversification^6^, but a later, larger-scale study did not^10^. A paleontological analysis also questioned whether herbivory drives insect diversification, but based on the number of insect families over time rather than on patterns of species richness or diversification^9^. More recent studies have emphasized wings and complete metamorphosis (holometaboly) as drivers of insect diversification^12^,^15^, but without quantitative comparison of their effects relative to those of herbivory. Thus, the importance of herbivory to broad-scale insect diversification is currently uncertain, despite extensive work relating herbivory (and shifts in host plant species) to speciation at smaller phylogenetic scales^16^,^17^,^18^.

Here, we take advantage of increasing phylogenetic information for insects^10^,^11^,^12^,^13^ and more powerful comparative methods to address the relationship between herbivory and insect diversification across scales. Our results support the importance of herbivory in increasing insect diversification and helping to shape patterns of species richness among insect orders. However, the impact of herbivory on diversification is more variable within orders, with significant effects within some clades (Diptera, Hemiptera) but not others (Coleoptera, Hymenoptera, Orthoptera). Sister-clade comparisons support these two basic patterns. Wings and holometaboly may also be important drivers of insect diversification, but unlike herbivory, their impact depends strongly on the phylogeny considered. Thus, we demonstrate the importance of herbivory for insect diversification, while also showing that it is not the sole explanation for the remarkable diversity of insects and arthropods. More broadly, our results show that local-scale ecology (e.g., diet, species interactions) helps drive large-scale patterns of species richness in the Earth's most diverse clade of organisms and over a time span of ∼500 million years.

## Results and Discussion

### Overview

**Figure 1:**
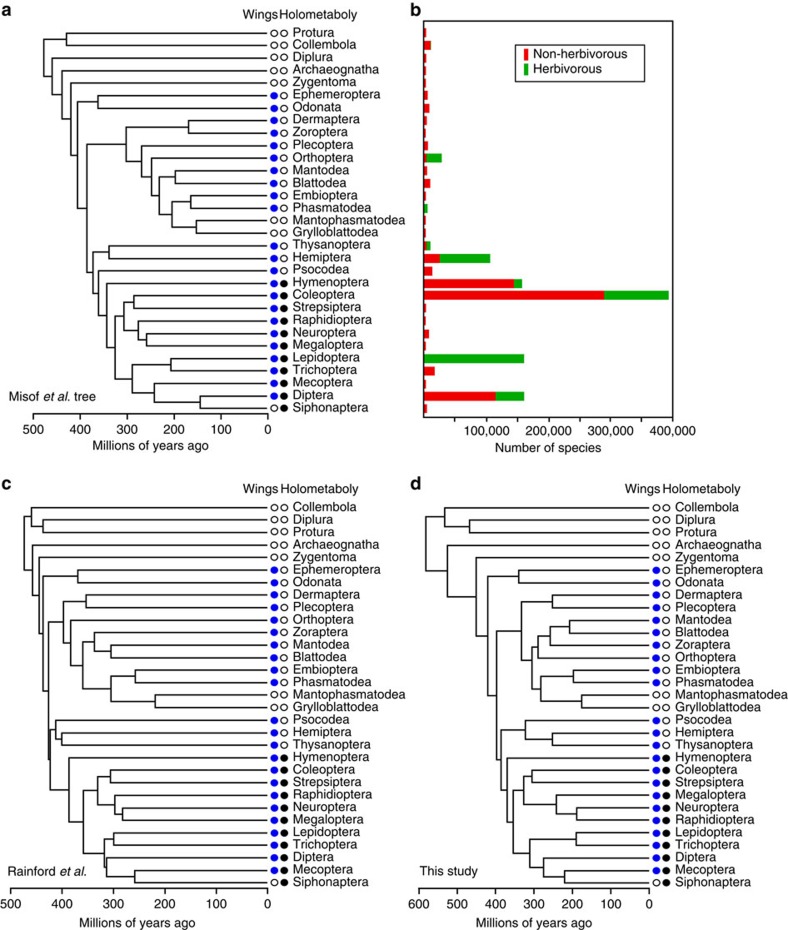
Phylogenies of insect orders and distributions of species richness and herbivory and other traits among clades. (**a**) Phylogeny from Misof *et al.*^11^, showing distribution of wings and holometaboly among clades, (**b**) species richness of hexapod clades, including the number of herbivorous and non-herbivorous species, (**c**) phylogeny from Rainford *et al.*^12^, (**d**) phylogeny from this study.

We used time-calibrated trees (Fig. 1) of hexapod orders (insects and close relatives) from phylogenomic data^11^, from extensive taxon sampling^12^, and our own analyses (Supplementary Figs 1 and 2, Supplementary Tables 1–3, Supplementary Methods and Supplementary Data 1–3). We estimated net diversification rates of insect orders using well-established methods^19^, based on summaries of described species numbers^14^ and estimated clade ages from these trees (Supplementary Tables 4–7). We also estimated the proportion of herbivorous species in each order (Supplementary Table 4 and Supplementary Methods). We then tested for a relationship between diversification rates of clades and their proportions of herbivorous species, using phylogenetic generalized least squares regression (PGLS)^20^. Using this general approach, we can include all insect orders (not just sister clades) and estimate how much variation in diversification rates can be explained by herbivory (and other traits), while accounting for the phylogenetic non-independence of clades. We also carried out similar analyses within most orders that are variable for herbivory (Coleoptera, Diptera, Hemiptera, Hymenoptera, Orthoptera), taking advantage of extensive family-level trees for these clades^10^,^12^,^13^ (Supplementary Data 4–8). We also confirmed these results with the more traditional approach of sister-clade comparisons^5^.

### Patterns of diversification among orders

We find a strong positive relationship between diversification rates of insect orders and their proportions of herbivorous species (PGLS: *P*<0.0005, *n*=31). Herbivory explains ∼25–35% of the variation in diversification rates among orders (Table 1 and Supplementary Tables 8–10). Exact values depend on the phylogeny used and the assumed ratios of extinction to speciation when estimating diversification rates. Similarly, herbivory explains ∼25% of the variation in species richness, and richness is strongly linked to diversification rates (PGLS: *r*^2^=0.62–0.89, *n*=31; Supplementary Tables 8–10). Thus, despite past controversy, we show that herbivory has a significant, positive impact on insect diversification and diversity patterns.

These results help explain the striking variation in current species diversity of insect orders (Supplementary Table 4). Insect orders vary from <50 described species (Grylloblattodea, Mantophasmotodea, Zoraptera) to >380,000 (Coleoptera)^14^. All three species-poor orders have no known herbivorous species. Conversely, all orders with >100,000 described species have at least 7% herbivorous species (Coleoptera, Diptera, Hemiptera, Hymenoptera, Lepidoptera), with high proportions in Hemiptera and Lepidoptera (∼80 and ∼100% herbivorous). Moreover, Lepidoptera has among the fastest diversification rates of any insect order (Supplementary Tables 5–7), possibly faster than Coleoptera (∼26% herbivorous species; Supplementary Table 4). The relatively rapid rates in predominantly herbivorous clades strongly suggest that the positive relationship between herbivory and diversification observed arises because herbivory does influence diversification, and does not simply arise because clades with higher diversification rates have more species and therefore more opportunities to evolve herbivory (for example, there was presumably only a single origin of herbivory in the rapidly diversifying Lepidoptera). The proportion of herbivorous species within orders need not be related to how many origins of herbivory they contain (see discussion in Supplementary Methods).

### Patterns of diversification within orders

We also explored the relationship between herbivory and diversification within five orders that are both highly diverse and highly variable for herbivory (∼7–94% herbivorous species). In beetles (Coleoptera), we find no relationship between herbivory and diversification rate (Supplementary Tables 11 and 12), as found by Hunt *et al.*^10^ using eight sister-clade comparisons. Our approach has greater statistical power (*n*=321 clades), but still shows no significant relationship. In contrast, we find a significant relationship between herbivory and diversification among 142 fly families (Diptera), with herbivory explaining ∼12–14% of the variation in diversification rates (Supplementary Tables 13 and 14). We find a significant but relatively weak relationship in Hemiptera (∼9% of variation explained; Supplementary Tables 15 and 16) and no significant relationships in Hymenoptera and Orthoptera (Supplementary Tables 17–20). These latter two orders are mostly invariant for herbivory (∼7% and ∼94% herbivorous; Supplementary Table 4). Although these results might imply that rate differences between herbivorous and non-herbivorous clades increase at deeper phylogenetic scales, we find that both across orders and within Diptera and Hemiptera, clades with herbivorous species have diversification rates ∼1.5–2 times higher than those in non-herbivorous clades (Supplementary Table 21).

### Sister-clade comparisons

These results are also generally supported using the traditional approach of sister-clade comparisons (Supplementary Table 22). Given that sister clades have identical stem ages, a tendency for clades with higher proportions of herbivorous species to have more species overall suggests that herbivory drives faster diversification rates. Among insect orders, five pairs of sister clades differ in their proportions of herbivorous species (Fig. 1; Phasmatodea–Embioptera, Lepidoptera–Trichoptera, Diptera–Siphonaptera, Hemiptera–Thysanoptera and Coleoptera–Strepsiptera). In all five pairs, the order with a higher proportion of herbivorous species has more species. This pattern is significant using a non-parametric paired, one-tailed sign test (*n*=5, *P*=0.0312). Within orders, the results generally parallel those based on explicit estimates of net diversification rates. Specifically, Diptera show a strong pattern (*n*=19 sister-clade pairs, with higher richness in the herbivorous family in 13 pairs; *P*=0.0022), whereas other orders are ambiguous or have too few suitable pairs (Coleoptera: *n*=21 pairs, 13 with higher richness in the herbivorous clade, *P*=0.1916; Hemiptera: *n*=4 pairs, 2 with higher herbivorous richness, *P*=1.00; Hymenoptera: *n*=4 pairs, none showing higher hervivorous richness; Orthoptera: *n*=3 pairs, 2 showing higher herbivorous richness). Importantly, when all non-overlapping sister-clade pairs are considered, both within orders (*n*=51 pairs) and between orders (*n*=2; Phasmatodea-Embioptera, Lepidoptera-Trichoptera), the pattern of higher richness in the more herbivorous clade is supported (*n*=53, *P*=0.0135). In summary, these results broadly support those from estimated diversification rates, and show that the effect of herbivory on diversification is supported when combining results both within and between orders. However, sister-clade comparisons do not quantify how much variation in diversification rates herbivory explains, nor do they allow including multiple traits.

### Impacts of wings and holometaboly on diversification

The effect of herbivory on diversification is highly significant among insect orders, but still explains less than half of the variation in rates (Table 1). However, the inclusion of one or two additional variables can explain much of the remaining variation. Specifically, phylogenetic multiple regression models including herbivory, the presence of wings and complete metamorphosis (holometaboly) explain ∼50–60% of the variation in diversification rates among orders, but only herbivory is consistently important (Table 1 and Supplementary Tables 8–10). Using the tree of Misof *et al.*^11^, only herbivory and wings contribute significantly to the model. For that of Rainford *et al.*^12^, only herbivory and holometaboly contribute significantly. For the tree generated here, all three contribute significantly. Thus, all three trees agree that herbivory is important, whereas the importance of wings and holometaboly are sensitive to the phylogeny considered.

### Sources of error

We note several potential sources of error in our study, but none should overturn our major conclusions. First, the richness estimates used here are doubtless underestimates of actual species numbers. However, our results on variation in diversification rates among clades should be supported as long as numbers of described and undescribed species in each clade are roughly proportional. Similarly, diet has not been described for all insect species either, but we merely assume that the proportions estimated here will remain similar as more species (and diets) are described. Also, our estimates of diversification rates may not be strictly accurate, as they assume rates are constant within clades over time^19^. However, our primary interest is explaining variation in these rates, not their absolute values. Moreover, our conclusions stand if we ignore rates entirely and simply consider species richness (Supplementary Tables 8–10), and are also supported by sister-clade comparisons.

### Mechanisms linking diet and other traits to diversification

Why might herbivory, wings and holometaboly increase diversification? For herbivory, two important factors may be (i) the great biomass offered by plants as a food resource, which is utilized by few insect orders overall^5^, and (ii) the diversity of plants, and the tendency for each insect species to utilize only a finite set of the available plant species at any given location (for example, due to plant defenses^2^,^17^). Wings may increase diversification by increasing dispersal ability, which could lead to lower extinction rates and higher speciation rates (reviewed by Mayhew^7^). Holometaboly may be important in allowing adults and juveniles to specialize on more and different resources, possibly increasing speciation rates^7^. Our results do not directly address these hypotheses but do provide important confirmation that herbivory does indeed increase insect diversification (along with wings and/or holometaboly). More work is clearly needed on the potential causal roles of all three variables in diversification.

### Explaining exceptional arthropod diversity

Moreover, we caution that neither herbivory, holometaboly nor wings are the sole explanations for the remarkable diversity of arthropods among animals. Aside from arthropods, the most diverse animal phyla^21^ are Annelida (∼18,000 species), Chordata (∼69,000) and Mollusca (∼73,000). Thus, even without insects, arthropods would be the most species-rich animal phylum, given their other species-rich higher taxa (for example, Arachnida: ∼112,000 species; Crustacea: ∼70,000)^14^. Thus, there must be some intrinsic trait in arthropods (besides herbivory, holometaboly, and wings) that helps drive high arthropod richness. Such traits might include the exoskeleton, body segmentation or their diverse and adaptable appendages^7^. Overall, we speculate that the exceptional richness of arthropods may be explained by a ratcheting of successive factors that each increase diversification, beginning with one or more factors present broadly across arthropods, followed by herbivory and possibly wings and/or holometaboly within insects.

Nevertheless, much of terrestrial arthropod diversity may not be fully independent of insect herbivory. The small size of predaceous arthropods may limit their potential prey to other taxa of similar size, including herbivorous insect species^22^. Thus, even if insect herbivory is not the sole or even primary explanation for high insect and arthropod diversity, it may still be critically important for arthropod foodwebs and for the diversity of many other insect and arthropod clades.

### Summary

Here we show that herbivory increases insect diversification at the broadest phylogenetic scales, and helps explain patterns of species richness across a clade containing more than half of all living species. More generally, our results show that local-scale ecological factors can strongly influence diversification over vast temporal scales (∼500 million years), and even more so than at shallower timescales. Further, we show that linkages between ecology and diversification rates can be important for understanding large-scale richness patterns^23^ despite recent controversy^24^.

## Methods

### Overview

We tested for a relationship between herbivory and net diversification rates among insect orders and within five orders that show variability for herbivory. We also performed sister-clade comparisons^5^. We used three estimates of insect phylogeny to obtain ages of orders and to account for phylogenetic non-independence of clades with PGLS analyses. We used the phylogenomic tree of Misof *et al.*^11^, the tree of Rainford *et al.*^12^ based on extensive taxon sampling and our own estimate. These trees allowed us to address the robustness of our results to reasonable variation in topologies and ages. Details of our tree estimation methods are described in the Supplementary Methods. The three topologies and dates are generally similar to each other and to other recent estimates (see Supplementary Methods).

For insect orders, we estimated the proportion of herbivorous species through a survey of the literature to the family level (for orders that vary for this trait). Details are provided in the Supplementary Methods. Our estimates at the ordinal level are generally very similar to those by Grimaldi and Engel^22^ and Hendrix^25^ (Supplementary Table 4). However, the estimates in those studies lacked explicit supporting documentation and therefore we used only our own estimates.

### Estimating diversification rates

Many approaches are available for estimating diversification rates (reviewed by Morlon^26^). Here, we utilize a relatively simple but standard approach, using the method-of-moments estimator for stem-group ages^19^. Other approaches would be impractical for several reasons. First, most other approaches would require detailed species-level phylogenies within each order, which are not presently available. Some approaches can account for moderate proportions of unsampled species (for example, ∼50% with BiSSE^27^) but would still be impractical given the very high proportions of unsampled species here (>98%). Moreover, many of these approaches (for example, BiSSE^27^) require that species included in the trees be randomly sampled. However, for the available trees, species have apparently been sampled to maximize phylogenetic representation (that is, include all higher taxa) and thus violate the assumption of random sampling.

We focus on stem-group ages for two reasons. First, in many cases, taxon sampling within orders was not sufficient to ensure that the crown-group split within each clade was represented. Failing to include the crown-group split could lead to underestimating the crown-group age (that is, sampling a shallower clade within the order) and overestimating the diversification rate. Second, we are interested in the diversification over the entire history of each clade. For example, a clade might have a stem group age of 250 Ma, a crown-group age of 10 Ma and 30 species. Basing the diversification rate on the crown group only would suggest that this clade has a rapid diversification rate (for example, because of rapid speciation within the crown group). However, considering the stem-group age would suggest a much slower net rate, likely due to extinction of clades over this long history, even if recent diversification was relatively rapid. We think that considering the entire history is important for a more complete picture of diversification within a clade.

Some authors have criticized the method-of-moments estimator because it does not take into account variation in diversification rates within each clade over time^28^, although this approach remains widely used, even by its critics^29^. It has been suggested that these estimators should only be used if there is a significant relationship between clade age and species richness. However, variation in diversification rates should itself decouple clade age and species richness, making this a highly problematic (and unnecessary) requirement. As an alternative, it has been suggested that species richness should be used instead of estimated diversification rates^28^. We used this approach also to test if our results were robust.

An important consequence of variation in diversification rates over time is that diversification rates and species richness could be decoupled. For example, if rapid diversification in young clades is not sustained over time, young clades might have high diversification rates but low richness, and factors that influence diversification rates might be unrelated to factors that explain the overall richness of clades. To address this possibility, we tested for a relationship between species richness and diversification rates among clades^23^.

The method-of-moments estimator is based on the estimated age and species richness of each clade. We used the stem-group age of each clade (order) obtained from our time-calibrated phylogeny and those of Misof *et al.*^11^ and Rainford *et al.*^12^. We obtained the estimated species richness of each clade from estimates provided by Zhang^14^. This is merely a summary of the number of described species. We recognize that the actual species richness of every insect order is almost certainly underestimated by the current number of described species. However, our analysis does not require that we know the true number of species in each clade. As we are interested in whether herbivory increases diversification, we are interested in the relative richness of clades (given their age). Therefore, our assumption is not that we know the actual number of species in every clade, but instead that the number of described species among clades is roughly proportional to their actual richness. For example, we assume that when (and if) all species are described, insect orders that are currently species rich and species poor will continue to be relatively species rich and poor.

Estimates of net diversification rate incorporate the outcome of both speciation and extinction. Nevertheless, estimates of net diversification may be more accurate if they allow for some level of extinction^19^. Although estimating speciation and extinction rates from clade ages and species numbers alone is problematic, a standard approach is to assume different ratios of extinction to speciation rates (relative extinction fractions, epsilon) across the sampled clades. We estimated the diversification rate of each clade using three relative extinction fractions (epsilon=extinction/speciation). We used a fraction of 0 (no extinction), high extinction (*e*=0.90, for justification see Magallón and Sanderson^19^) and an intermediate value (*e*=0.50). Importantly, our results on the herbivory–diversification relationship were largely consistent across different values of epsilon.

We generally followed the ordinal-level taxonomy of Zhang^14^, with two exceptions. First, we included Collembola, Diplura and Protura in this analysis as if they were insects, although most classifications treat them as clades closely related to Insecta within Hexapoda, but not insects themselves. Second, we recognized Psocodea instead of separate orders for Psocoptera and Phthiraptera. Several recent studies have found that Phthiraptera are nested inside of Psocoptera^11^,^12^,^30^,^31^,^32^,^33^, and (to our knowledge) none have contradicted this finding. Note that Blattodea includes the termites (Isoptera) in our classification and that of Zhang^14^. We recognize that ‘order' is an arbitary taxonomic rank. However, the fact that we are generally relating herbivory to diversification rates (instead of raw richness) accounts for the possibility that different orders may have very different ages.

### Testing relationships between diversification and herbivory

We tested for a relationship between diversification rates and herbivory among clades using PGLS^20^ to account for the phylogenetic non-independence of clades. We first created a reduced tree among insect order for each phylogeny, by deleting all species in each order except for one. The choice of species to include was arbitrary, as all species in a clade have the same branch length (time) from the present day to the origin of that clade. We then tested for a relationship between the proportion of herbivorous species in each clade (independent variable) and the clade's rate of net diversification (dependent variable), using PGLS in the R package caper^34^, version 0.5. We did not arcsine-transform these proportions, as this transformation can be problematic^35^. Note that PGLS is also appropriate when the independent variable is categorical and the dependent variable is continuous^20^, making logistic regression unnecessary. For caper analyses, we used the estimated values of lambda^36^ and fixed kappa and delta at 1. We did not perform Bonferroni corrections, as most of the large numbers of analyses within tables were associated with replicating analyses to address the robustness of our results to different relative extinction fractions and different topologies. We also present Akaike Information Criterion (AIC) values as an alternative approach to assessing statistical significance.

We also tested whether two other variables might contribute to variation in diversification rates among insect orders: the presence or absence of wings and the presence or absence of complete metamorphosis (holometaboly). These variables have been emphasized in recent studies as the main drivers of insect diversification^11^,^12^,^15^. To code the presence of wings among orders, we utilized the summaries of these character states in Grimaldi and Engel^22^. For simplicity, we coded wings as present in a clade if functional wings (enabling powered flight) were present in any extant species, even if wings were not present in all species. Therefore, wings were considered to be present in all clades of Pterygota (all Hexapoda excluding Collembola, Diplura, Protura, Archeognatha and Zygentoma) except for Siphonaptera, Grylloblattodea and Mantophasmatodea. For complete metamorphosis, we simply coded all clades in Holometabola as having this character state (holometaboly). We tested for the separate effects of herbivory, wings and holometaboly on diversification rates using PGLS and then tested for their combined effects on diversification rates using phylogenetic multiple regression in caper.

The phylogenies used in these analyses are presented in nexus format in Supplementary Data 1–3. Data on herbivory and species numbers are summarized in Supplementary Table 4. Data on stem ages and diversification rates are presented in Supplementary Table 5 (for the tree of Misof *et al.*^11^), Supplementary Table 6 (for the tree of Rainford *et al.*^12^) and Supplementary Table 7 (for our tree). Results of the PGLS analyses are shown in Supplementary Tables 8–10.

### Analyses of herbivory and diversification rates within orders

We also tested for a relationship between herbivory and diversification in most of the insect orders that are variable for the presence or absence of herbivory: Coleoptera, Diptera, Hemiptera, Hymenoptera and Orthoptera. Lepidoptera and Collembola are effectively invariant for the presence of herbivory, and we did not include Thysanoptera because the most recent and extensive phylogeny among families^12^ included too few families (*n*=4) to allow for a rigorous comparative analysis. Methods for estimating diversification rates and conducting PGLS regression generally followed those across orders, as described above.

For Coleoptera, we used the time-calibrated phylogeny of beetle families and subfamilies from the study by Hunt *et al.*^10^, from their Fig. 3. Their sampling encompassed >80% of all families, >60% of subfamilies and >95% of species^10^. They also included data on species richness and feeding habits for almost all higher taxa in this tree (their Supplementary Table 8). Notably, they considered xylophagy as distinct from herbivory. We followed these authors in only considering taxa feeding on living tracheophyte tissues to be herbivorous. For some clades, they listed multiple feeding types as present, without quantifying specific proportions. In these cases, we arbitrarily assumed that different feeding types were at equal frequencies within each clades (for example, if both herbivorous and predaceous habits were listed, we consider this clade to be 50% herbivorous). However, we also performed an analysis coding clades as herbivorous (state 1) or not (state 0), and any occurrence of herbivory in the clade was coded as herbivory present, even if other diets were also present. This approach provided a parallel to the methodology used for Diptera (see below). For six higher taxa, they listed the feeding habits as unknown. These taxa were excluded from the comparative analyses. Furthermore, Hunt *et al.*^10^ included a few subfamilies that their tree suggested were not monophyletic. In these cases, they took the estimated number of described species in each subfamily and assigned equal numbers of species to each taxon. Here, we took a different approach. Rather than using non-monophyletic subfamilies with uncertain ages and species numbers, we simply used the entire family as a terminal unit in these cases (combining the monophyletic and non-monophyletic subfamilies). We did this for the Cryptophagidae (represented in our tree by Cryptophagiinae), Monotomidae (represented by Monotominae), Sphindidae (represented by *Sphindus*) and Hydraenidae (represented by *Hydraena*). For Elateridae, we combine the two representatives of Denticollinae into one subfamily (represented by *Denticollis*). For Leiodidae, we combine the subfamilies Cholevinae and Leiodinae into one clade (represented by *Nargus*). The final analysis included 321 higher taxa of beetles. We then estimated net diversification rates for each clade given the species numbers for each clade they provided and the stem ages from their tree, using the method-of-moment estimator as described above. We tested for a relationship between herbivory and diversification rates, again using PGLS. The reduced tree used in these analyses is shown in nexus format in Supplementary Data 4 and the matching data on species richness, stem ages, diversification rates and herbivory are shown in Supplementary Table 11.

In theory, we could have carried out the analyses of Coleoptera using the tree of Rainford *et al.*^12^. However, this would have required amalgamating higher taxa (Hunt *et al.*^10^ generally used subfamilies, whereas Rainford *et al.*^12^ used families), and therefore dramatically reduced sample sizes and statistical power (*n*=321 versus 141 taxa). Furthermore, this amalgamation of subfamilies would have led to even less precise estimates of the proportion of herbivory in each taxon.

For Diptera, we used the data on phylogeny, divergence times, species richness and diets for dipteran families (∼90% sampled, ∼97% of species included) provided by Wiegmann *et al.*^13^. However, some minor modifications were necessary (see Methods in Supplementary Information). We followed Wiegmann *et al.*^13^ and coded families as herbivorous if they were listed as herbivorous (phytophagous) in their Fig. 1. We then estimated diversification rates for each family using the method-of-moment estimator for stem ages^19^, and then tested for a relationship between herbivory and diversification rates using PGLS (see above). The tree used in these analyses is shown in nexus format in Supplementary Data 5 and data on species richness, herbivory, stem ages and diversification rates are shown in Supplementary Table 13. The results are shown in Supplementary Table 14.

Again, these analyses could have been repeated using the dipteran tree of Rainford *et al.*^12^. However, this would have reduced the included taxa from 142 to 118 (making it difficult to tell whether any differences in results were due to different trees or to reduced sample sizes instead).

For Hemiptera, we used the data on phylogeny, clade ages and species richness for the 93 hemipteran families from Rainford *et al.*^12^ (including ∼73% of ∼128 families^1^, ∼95% of species). As all but five of these families have either 0% or 100% herbivorous species (see Methods in Supplementary Information), we treated the presence of herbivory in a family as a binary variable. The tree used in these analyses is shown in nexus format in Supplementary Data 6 and the matching data on species richness, herbivory, stem ages and diversification rates are shown in Supplementary Table 15. The results are shown in Supplementary Table 16.

For Hymenoptera, we used the data on phylogeny, clade ages and species richness for 77 families from Rainford *et al.*^12^ (including ∼58% of ∼132 families^14^; ∼92% of species). Families were treated as herbivorous or not (binary variable), except for families thought to have a very low proportion of herbivorous species (see Methods in Supplementary Information). The tree used in these analyses is shown in nexus format in Supplementary Data 7 and the matching data on species richness, herbivory, stem ages and diversification rates are shown in Supplementary Table 17. The results are shown in Supplementary Table 18.

For Orthoptera, we used the phylogeny, clade ages and species richness from Rainford *et al.*^12^. However, we excluded two families (Rhipipterygidae and Xyronotidae, see above) lacking data on diet. In total, 26 families were included (∼65–87% of ∼30–40 families^21^,^22^; ∼96% of species). Families were treated as herbivorous or not (binary variable). The tree used in these analyses is shown in nexus format in Supplementary Data 8 and the matching data on species richness, herbivory, stem ages and diversification rates are shown in Supplementary Table 19. Results are shown in Supplementary Table 20.

### Sister-clade comparisons

To complement the analyses based on diversification rates and species richness, we also conducted sister-clade comparisons. Following previous authors^5^,^10^, we assumed that higher diversification rates in predominantly herbivorous clades would lead to greater species numbers in clades with a higher proportion of herbivorous species. Because sister clades share the same stem age, higher richness in one clade must be explained by a higher net diversification rate. The effect of herbivory on diversification is then manifested by a significant number of pairs showing higher richness in the more herbivorous clade (relative to a 50:50 expectation given no effect).

We first analysed patterns among orders. Five sister-pairs of orders differed in their proportions of herbivorous species (the minimum number for a significant pattern). For two pairs, each clade was largely invariant for the presence or absence of herbivory (Lepidoptera–Trichoptera and Phasmatodea–Embioptera), two pairs involved a clade lacking herbivory and another with a modest proportion of herbivorous species (Coleoptera–Strepsiptera and Diptera–Siphonaptera), and in one pair both clades were dominated by herbivorous species but differed in their proportions (Hemiptera–Thysanoptera).

We then addressed patterns within the five orders that showed major variation in herbivory among their subclades (Coleoptera, Diptera, Hemiptera, Hymenoptera, Orthoptera). Within each order, we identified sister pairs of clades in which herbivory was present in one (regardless of proportion) and absent in the other. The same phylogenies, clades and herbivory data within each order were used as described above. When selecting sister clades, we focused on the least inclusive pair of clades that differed for the presence of herbivory (usually pairs of families or subfamilies), to maximize sample sizes and statistical power. In some cases, we used clades including multiple families or subfamilies, to maximize sample sizes. Nevertheless, the number of pairs was still very limited in Hemiptera, Hymenoptera and Orthoptera (*n*=4, 4 and 3), largely because subclades within these orders are relatively invariant for herbivory. These sample sizes were too small for separate statistical analyses of these orders, but the data were used in an analysis that spanned all five orders and the two pairs of orders excluding these five. Sample sizes were larger for Diptera (*n*=19) and Coleoptera (*n*=21), and these orders were analyzed separately. In each case, data on species richness (summarized in Supplementary Table 22) were analyzed using a non-parametric one-tailed, paired sign test (in Statview).

## Additional information

**How to cite this article:** Wiens, J. J. *et al.* Herbivory increases diversification across insect clades. *Nat. Commun.* 6:8370 doi: 10.1038/ncomms9370 (2015).

## Supplementary Material

Supplementary Information Supplementary Figures 1-2, Supplementary Tables 1-22, Supplementary Methods and Supplementary References

Supplementary Data 1 Phylogeny of insect orders based on figure 1 of Misof and colleagues^11^

Supplementary Data 2 Phylogeny of insect orders based on the study of Rainford and colleagues^12^

Supplementary Data 3 Time-calibrated phylogeny of insect orders based on the results of this study

Supplementary Data 4 Reduced phylogeny of beetle subfamilies and families used in this study as modified from Hunt and colleagues^10^

Supplementary Data 5 Reduced phylogeny of dipteran families used in this study as modified from Wiegmann and colleagues^13^

Supplementary Data 6 Reduced phylogeny of hemipteran families used in this study as modified from Rainford and colleagues^12^

Supplementary Data 7 Reduced phylogeny of hymenopteran families used in this study as modified from Rainford and colleagues^12^

Supplementary Data 8 Reduced phylogeny of orthopteran families used in this study as modified from Rainford and colleagues^12^

## Figures and Tables

**Table 1:** Relationships between diversification and herbivory among insect orders based on PGLS.

**Tree**	**Variables**	***r***^2^	***P***	**AIC**
Misof *et al.*^11^	Diversification rate∼herb.	0.3056	0.0001	229.0834
	Diversification rate∼wings	0.3960	<0.0001	224.7614
	Diversification rate∼holometaboly	0.0888	0.0755	237.5065
	Diversification rate∼herb. +wings	0.5454	<0.0001	217.9511
	Diversification rate∼herb. +wings +holometaboly	0.5764	<0.0001	217.7636
Rainford *et al.*^12^	Diversification rate∼herb.	0.2692	0.0003	207.1512
	Diversification rate∼wings	0.2558	0.0005	207.1406
	Diversification rate∼holometaboly	0.2330	0.0010	208.0750
	Diversification rate∼herb. +holometaboly	0.4840	<0.0001	197.7849
	Diversification rate∼herb. +wings +holometaboly	0.5414	<0.0001	196.1284
This study	Diversification rate∼herb.	0.3177	<0.0001	226.5232
	Diversification rate∼wings	0.1656	0.0079	235.0378
	Diversification rate∼holometaboly	0.3429	<0.0001	227.6331
	Diversification rate∼herb.+wings	0.5157	<0.0001	220.1742
	Diversification rate∼herb.+wings +holometaboly	0.6101	<0.0001	215.4544

AIC, Akaike Information Criterion; Div., diversification; Herb., herbivory; PLGS, phylogenetic generalized least squares regression.

Comparisons of the fit of different models testing the independent variables that predict net diversification rates (dependent variable) among insect orders, based on three phylogenetic trees. Results are shown using a relative extinction fraction of 0.90 to estimate diversification rates, which typically has the best fit to the data.
