# Supplementary material for: Herbivory increases diversification across insect clades
Source: Nat Commun. 2015 Sep 24;6:8370. doi: 10.1038/ncomms9370 (PMC4598556; doi:10.1038/ncomms9370)
Supplement: Supplementary Information — Supplementary Figures 1-2, Supplementary Tables 1-22, Supplementary Methods and Supplementary References [file ncomms9370-s1.pdf]

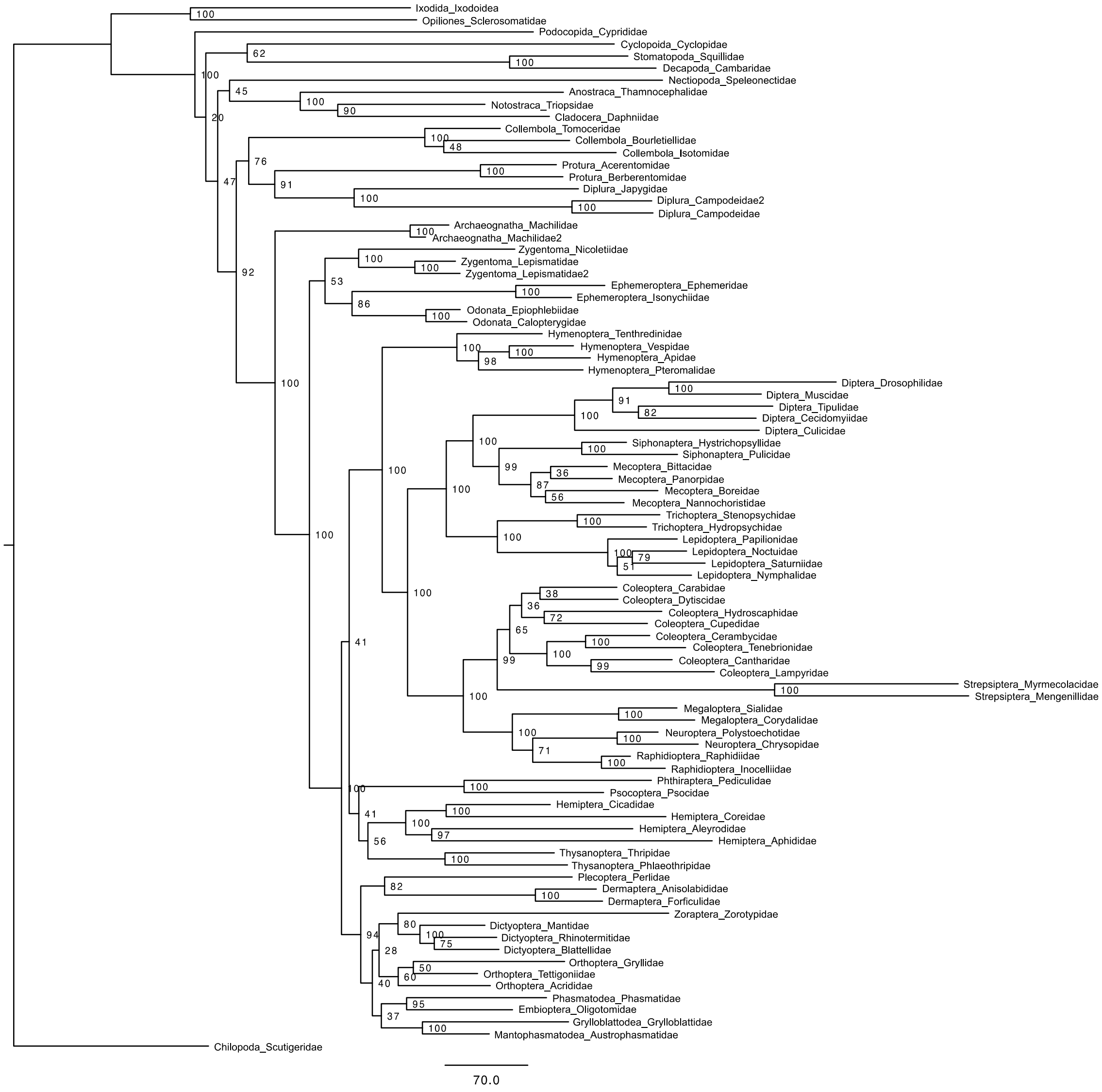

Supplementary Figure 1. Phylogeny of hexapod clades and outgroups based on a concatenated maximum likelihood analysis of 11 genes. Numbers at nodes indicate bootstrap support values.

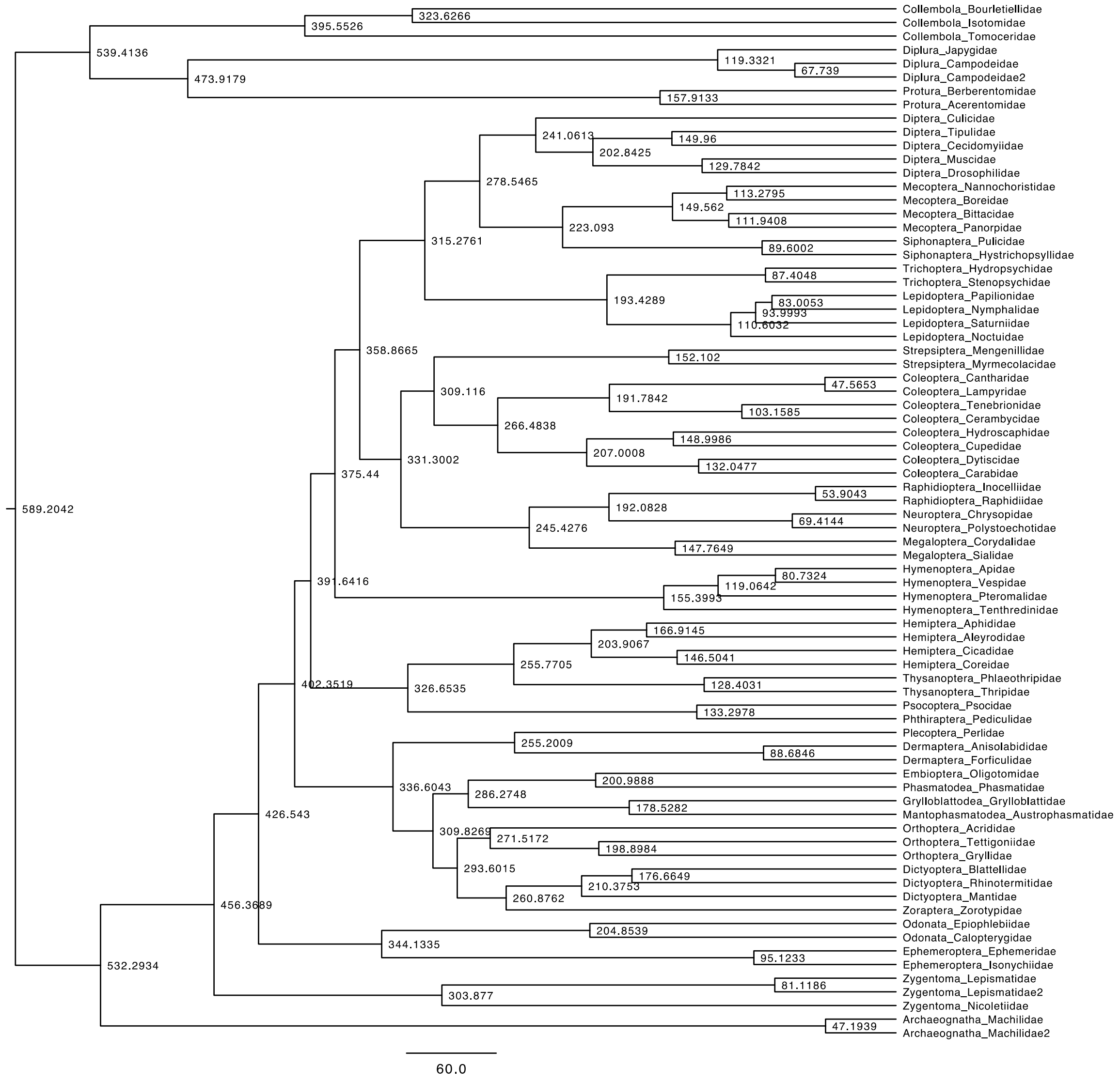

Supplementary Figure 2. Time-calibrated phylogeny of hexapod clades and outgroups based on a concatenated Bayesian analysis of 11 genes. Numbers at nodes indicate mean estimated ages of clades.

Supplementary Table 1. GenBank numbers for phylogenetic analysis.

Taxon name	Species included	*18S*	*28S*
Archaeognatha_Machilidae	*Pedetontus unimaculatus, P. okajimae, P. saltator*	EU368614	EU376055
Archaeognatha_Machilidae2	*Petrobiellus takunagae*		
Coleoptera_Cantharidae	*Chauliognathus opaca*	HM156710	HM156702
Coleoptera_Carabidae	*Bembidion louisella, B. perspicuum, B. transversale*	EF648670	GQ503347
Coleoptera_Cerambycidae	*Strangalia bicolor, S. luteicornis*	HM156709	HM156701
Coleoptera_Cupedidae	*Tenomerga cinerea, Priacma serrata*	EU797417	EU677675
Coleoptera_Dytiscidae	*Laccophilus poecilus, L. pictus, Eretes sticticus*	AJ318714	KF978822
Coleoptera_Hydroscaphidae	*Hydroscapha natans*	HM156708	
Coleoptera_Lampyridae	*Lucidina biplagiata, Lampyrinae sp.*	AB298844	DQ198759
Coleoptera_Tenebrionidae	*Tribolium castaneum*	HM156711	HM156703
Collembola_Bourletiellidae	*Bourletiella hortensis, Pseudobourletiella spinata*	DQ016568	
Collembola_Isotomidae	*Folsomia candida, Isotomurus hadriaticus*	AY555515	EU914252

Collembola_Tomoceridae	*Tomocerus* sp., *Pogonognathellus flavescens*	EU368607	EU376053
Dermaptera_Anisolabididae	*Euborellia plebeja, Euborellia femoralis*	KC413782	AY707393
Dermaptera_Forficulidae	*Forficula hiromasai, Forficula auricularia*	Z97594	EU426876
Dictyoptera_Blattellidae	*Blatella germanica, Blattella nipponica*	AF005243	FJ806519
Dictyoptera_Mantidae	*Tenodera aridifolia, Hierodula membranacea*	FJ806336	FJ806534
Dictyoptera_Rhinotermitidae	*Reticulitermes speratus, Reticulitermes santonensis, Coptotermes formosanus*	EU253792	FJ806531
Diplura_Campodeidae	*Lepidocampa weberi, Eumesocampa frigilis, Campodea tillyardi*	AF173234	EF192436
Diplura_Campodeidae2	*Metriocampa sp, M. kuwayamai*	AY596368	AY596385
Diplura_Japygidae	*Occasjapyx japonicus, Metajapyx subterraneus, Catajapyx sp.*	AF005456	EF192438
Diptera_Cecidomyiidae	*Mayetiola destructor*	KC177284	KC177649
Diptera_Culicidae	*Anopheles gambiae*	KC177276	KC177663
Diptera_Drosophilidae	*Drosophila melanogaster*	KC177303	KC177803

Diptera_Muscidae	*Musca domestica*	GQ465780	AY123358
Diptera_Tipulidae	*Tipula sp, T. abdominalis, Holorusia rubiginosa*	X89496	FJ040553
Embioptera_Oligotomidae	*Aposthonia japonica, Aposthonia gurneyi*	JQ907221	JQ906990
Ephemeroptera_Ephemeridae	*Ephemera japonica, Hexagenia limbata, Hexagenia rigida*	AF461253	AF461222
Ephemeroptera_Isonychiidae	*Isonychia japonica, Isonychia sp*	AY338708	AY338665
Grylloblattodea_Grylloblattidae	*Galloisiana yuasai, Galloisiana sp*	KC142290	KC142473
Hemiptera_Aleyrodidae	*Bemisia tabaci*	Z15052	
Hemiptera_Aphididae	*Acyrthosiphon pisum, Aphis glycines*	U27819	JQ259057
Hemiptera_Cicadidae	*Cryptotympana facialis, Cicadetta calliope, Cicadetta celis, Cicadidae sp.*	AB911206	JQ309936
Hemiptera_Coreidae	*Anacanthocoris striicornis, Aulacosternum nigrorubrum*	AY252258	AY252500
Hymenoptera_Apidae	*Apis mellifera*	AY703484	AY703551
Hymenoptera_Pteromalidae	*Nasonia vitripennis*	GQ410677	GQ374784
Hymenoptera_Tenthredinidae	*Ametastegia equiseti, Athalia rosea, Neocolochelyna itoi*	AB064266	GQ374683

Hymenoptera_Vespidae	*Vespa analis insularis, Vespula pensylvanica, Metapolybia cingulata*	AY859613	EF013011
Lepidoptera_Noctuidae	*Helicoverpa armigera, H. assulta, Heliothis virescens*	EU057177	FJ817830
Lepidoptera_Nymphalidae	*Danaus plexippus, Bicyclus anynana*	AF394668	Genome
Lepidoptera_Papilionidae	*Papilio polytes, P. xuthus, P. troilus, Mimoides branchus*	AF286299	AF423920
Lepidoptera_Saturniidae	*Bombyx mori*	DQ347470	Genome
Mantophasmatodea_Austrophasmatidae	*Karoophasma biedouwensis, Austrophasma gansbaaiensis*		
Mecoptera_Bittacidae	*Australobittacus sp., Apterobittacus apterus*	AF423875	AF423926
Mecoptera_Boreidae	*Boreus brumalis, Boreus sp*	AF423883	AF423936
Mecoptera_Nannochoristidae	*Nannochorista sp., N. neotropica*	AF334799	AF338261
Mecoptera_Panorpidae	*Panorpa sp., P. cognata, Panorpa takenouchii*	AF423897	AF423954
Megaloptera_Corydalidae	*Nigronia sp., N. serricornis, Protohermes grandis, Archichauliodes guttiferus, Acanthacorydalis sp*	EU815263	

Megaloptera_Sialidae	*Sialis hamata, Sialis californica*	HM156713	AY521793
Neuroptera_Chrysopidae	*Chrysoperla nipponensis, Chrysoperla carnea, Italochrysa insignis, Chrysoperla lucasina*	AY620037	KC312268
Neuroptera_Polystoechotidae	*Platystoechotes sp.*	EU815274	
Odonata_Calopterygidae	*Mnais pruinosa pruinosa, Calopteryx splendens*	Y12898	JN615308
Odonata_Epiophlebiidae	*Epiophlebia superstes*	EU055226	FJ009956
Orthoptera_Acrididae	*Anacridium aegyptium, Patanga japonica, Locusta migratoria, Pternoscirta sauteri, Nomadacris japonica*	AY626908	EU414723
Orthoptera_Gryllidae	*Teleogryllus emma, Acheta domesticus*	X95741	AY859544
Orthoptera_Tettigoniidae	*Euconocephalus varius, E. thunbergi*	KF570794	KF570926
Phasmatodea_Phasmatidae	*Phraortes illepidus, Acanthoxyla geisovii*	KC413821	EU543517
Phthiraptera_Pediculidae	*Pediculus humanus corporis*	FJ267399	Genome
Plecoptera_Perlidae	*Oyamia lugubris, Perlinella drymo*	EF622793	EF622949
Protura_Acerentomidae	*Nipponentomon nippon,*	EU368597	EF199976

	Acerentomon franzi		
Protura_Berberentomidae	*Baculentulus morikawai, Baculentulus tianmushanensis*	AY037169	EF192433
Psocoptera_Psocidae	*Metylophorus purus, Metylophorus sp., Cerastipsocus venosus*	EF662272	AY252428
Raphidioptera_Inocelliidae	*Inocellia japonica, I. crassicornis, Parainocellia bicolor*	EU815245	HM543338
Raphidioptera_Raphidiidae	*Mongoloraphidia martrynovae, M. manasiana, M. nomadobia*	EU815252	HM543347
Siphonaptera_Hystrichopsyllidae	*Neotyphloceras crassipina chilensis, Neotyphloceras sp.*	EU336079	EU336187
Siphonaptera_Pulicidae	*Ctenocephalides canis, C. felis*	AF423914	AF423974
Strepsiptera_Mengenillidae	*Mengenilla sp, M. moldrzyki*	HM156715	HM156705
Strepsiptera_Myrmecolacidae	*Caenocholax sp, Stichotrema asahinai*	HM156714	HM156704
Thysanoptera_Phlaeothripidae	*Gynaikothrips ficorum, Gynaikothrips sp*	KC512963	KC513064
Thysanoptera_Thripidae	*Thrips palmi, Frankliniella fusca*	KC413830	AB277569
Trichoptera_Hydropsychidae	*Hydropsyche occidentalis, H. phalerata*	AF286291	AF338267

Trichoptera_Stenopsychidae	*Stenopsyche marmorata, Stenopsychodes mjobergi*	AF436472	AF436359
Zoraptera_Zorotypidae	*Zorotypus weidneri, Zorotypus sp.*	JQ259056	JN192452
Zygentoma_Lepismatidae	*Thermobia domestica, Ctenolepisma lineata*	AF370790	AY338683
Zygentoma_Lepismatidae2	*Isolepisma japonica*		
Zygentoma_Nicoletiidae	*Nipponatelura sp., Nicoletia meinerti, Anelpistina puertoricensis*	DQ280065	DQ280085
Ixodida_Ixodoidea	*Ixodes scapularis, Ixodes hexagonus*		JN018404
Opiliones_Sclerosomatidae	*Psathyropus tenuipes, Protolophus singularis*	JQ437034	EF028096
Anostraca_Thamnocephalidae	*Branchinella kugenumaensis, B. occidentalis*	AY744888	AY744895
Cladocera_Daphniidae	*Daphnia pulex, Daphnia pulicaria*	AF014011	DQ470613
Cyclopoida_Cyclopidae	*Cyclops vicinus, Cyclopidae sp, Acanthocyclops vernalis, A. viridis*	AY626999	AY210813
Decapoda_Cambaridae	*Procambarus clarkii*	AF436001	EU920970
Nectiopoda_Speleonectidae	*Speleonectes tulumensis*	EU370431	EU370446

Notostraca_Triopsidae	*Triops granarius, T. longicaudatus*	AF144219	AY157606
Podocopida_Cyprididae	*Cypridopsis vidua, Cypridopsis uenoi*	AB674998	AB674997
Stomatopoda_Squillidae	*Oratosquilla oratoria, Squilla empusa*	L81946	AY210842
Chilopoda_Scutigeridae	*Thereuonema tuberculata, Scutigera coleoptrata*	DQ222126	DQ222145

Taxon name	*EF-1a*	*CAD*	*AATS*
Archaeognatha_Machilidae		GQ888278	GQ885714
Archaeognatha_Machilidae2			
Coleoptera_Cantharidae	HM156720	EU677536	
Coleoptera_Carabidae	GQ503346	EU677541	
Coleoptera_Cerambycidae	HM156719	GQ265599	GQ265574
Coleoptera_Cupedidae	GQ503344	EU677525	
Coleoptera_Dytiscidae	HM156717	EU677528	
Coleoptera_Hydroscaphidae	HM156718	HM156726	
Coleoptera_Lampyridae			
Coleoptera_Tenebrionidae	HM156722	EU677538	XM_961372
Collembola_Bourletiellidae			
Collembola_Isotomidae	AJ009854		
Collembola_Tomoceridae	U90059	GQ888285	GQ885721

Dermaptera_Anisolabididae			
Dermaptera_Forficulidae	AY305464	EU020442	EU020714
Dictyoptera_Blattellidae		GQ265596	GQ265573
Dictyoptera_Mantidae	EU414699		
Dictyoptera_Rhinotermitidae	KC632472		
Diplura_Campodeidae	AF137388	GQ888248	
Diplura_Campodeidae2			
Diplura_Japygidae	AF137389	GQ888259	
Diptera_Cecidomyiidae	AF085227		KC176874
Diptera_Culicidae	XM_308429	XM_310823	XM_318757
Diptera_Drosophilidae	X06869	X04813	NM_205934
Diptera_Muscidae	GQ465788	GQ265585	GQ265564
Diptera_Tipulidae	AF423809	GQ265584	GQ265563
Embioptera_Oligotomidae			
Ephemeroptera_Ephemeridae	AY305469	GQ888266	GQ885706
Ephemeroptera_Isonychiidae			
Grylloblattodea_Grylloblattidae	KC142994		
Hemiptera_Aleyrodidae			
Hemiptera_Aphididae	FJ982418	XM_001943600	XM_001950396
Hemiptera_Cicadidae	AF313527		
Hemiptera_Coreidae			
Hymenoptera_Apidae	AF015267	XM_393888	XM_395392

Hymenoptera_Pteromalidae	NM_001172756	XM_001606665	XM_003427554
Hymenoptera_Tenthredinidae	AB253792	GQ265586/ GQ265587	GQ265565
Hymenoptera_Vespidae	GQ410714		
Lepidoptera_Noctuidae	U20135	GQ265592	GQ265570
Lepidoptera_Nymphalidae	GU365934	JQ784465	JQ783445
Lepidoptera_Papilionidae	AK402256	JQ784650	AK402820
Lepidoptera_Saturniidae	HM156723	EU032656	M55993
Mantophasmatodea_Austrophasmatidae	DQ531740		
Mecoptera_Bittacidae	AF423817	GQ265602	GQ265577
Mecoptera_Boreidae	AF423828	GQ265601	GQ265576
Mecoptera_Nannochoristidae	AF423848	GQ265593/ GQ265594	GQ265571
Mecoptera_Panorpidae	AF423851	GQ265595	GQ265572
Megaloptera_Corydalidae	AY620199	GQ265598	
Megaloptera_Sialidae	HM156721	EU860153	
Neuroptera_Chrysopidae	JQ519512	EU860117	
Neuroptera_Polystoechotidae		GQ265590	GQ265568
Odonata_Calopterygidae	DQ642970		
Odonata_Epiophlebiidae			
Orthoptera_Acrididae	AB583233		
Orthoptera_Gryllidae	GQ886692	GQ888230	GQ885680

Orthoptera_Tettigoniidae			
Phasmatodea_Phasmatidae	EU493025		
Phthiraptera_Pediculidae	XM_002423581	XM_002422513	XM_002426758
Plecoptera_Perlidae			
Protura_Acerentomidae	AH009876		
Protura_Berberentomidae			
Psocoptera_Psocidae			
Raphidioptera_Inocelliidae	AY620205	EU860116	
Raphidioptera_Raphidiidae	AY620204	GQ265597	
Siphonaptera_Hystrichopsyllidae	EU336293	GQ265607	GQ265579
Siphonaptera_Pulicidae	AF423870	GQ265581	GQ265561
Strepsiptera_Mengenillidae	HM156725	GQ265580	Genome
Strepsiptera_Myrmecolacidae	HM156724		
Thysanoptera_Phlaeothripidae	AF448301		
Thysanoptera_Thripidae		GQ265588	GQ265566
Trichoptera_Hydropsychidae	AF436621	GQ265591	GQ265569
Trichoptera_Stenopsychidae	AF436638		
Zoraptera_Zorotypidae			
Zygentoma_Lepismatidae	AF063405	GQ888240	GQ885686
Zygentoma_Lepismatidae2			
Zygentoma_Nicoletiidae	AY305472	GQ888270	
Ixodida_Ixodoidea	XM_002411102		XM_002433713

Opiliones_Sclerosomatidae	JQ437089		
Anostraca_Thamnocephalidae			
Cladocera_Daphniidae	NCBI_GNO_490103	NCBI_GNO_370084	NCBI_GNO_866034
Cyclopoida_Cyclopidae	AY305458		GQ885677
Decapoda_Cambaridae			
Nectiopoda_Speleonectidae	AF063416		EU020720
Notostraca_Triopsidae	U90058		EU020722
Podocopida_Cyprididae	AF063414	EU020449	
Stomatopoda_Squillidae			
Chilopoda_Scutigeridae	AY305478	GQ888280	GQ885716

Taxon name	*PGD*	*SNF*	*TPI*
Archaeognatha_Machilidae	GQ887107		GQ887963
Archaeognatha_Machilidae2			
Coleoptera_Cantharidae			
Coleoptera_Carabidae			
Coleoptera_Cerambycidae			
Coleoptera_Cupedidae	Genome		
Coleoptera_Dytiscidae			
Coleoptera_Hydroscaphidae			
Coleoptera_Lampyridae			
Coleoptera_Tenebrionidae	XM_966958	XM_963178	XM_970400

Collembola_Bourletiellidae			
Collembola_Isotomidae			
Collembola_Tomoceridae			
Dermaptera_Anisolabididae			
Dermaptera_Forficulidae	EU020886		EU020345
Dictyoptera_Blattellidae	GQ265621	GQ265633	GQ265647
Dictyoptera_Mantidae			
Dictyoptera_Rhinotermitidae		KC740865	KC571986
Diplura_Campodeidae	GQ887076		GQ887937
Diplura_Campodeidae2			
Diplura_Japygidae	GQ887087		GQ887947
Diptera_Cecidomyiidae	KC177492	KC177056	KF647619
Diptera_Culicidae	XM_313091	XM_320869	XM_321467
Diptera_Drosophilidae	M80598	NM_078490	NM_176587
Diptera_Muscidae	GQ265612	GQ265627	GQ265639
Diptera_Tipulidae	GQ265611	GQ265626	
Embioptera_Oligotomidae			
Ephemeroptera_Ephemeridae	GQ887095		GQ887954
Ephemeroptera_Isonychiidae			
Grylloblattodea_Grylloblattidae			
Hemiptera_Aleyrodidae			
Hemiptera_Aphididae	XM_001950219	NM_001162674	NM_001162204

Hemiptera_Cicadidae			
Hemiptera_Coreidae			
Hymenoptera_Apidae	XM_006566923	NM_001168336	NM_001097154
Hymenoptera_Pteromalidae	XM_001600883	XM_001606432	XM_001600069
Hymenoptera_Tenthredinidae	GQ265613	GQ265628	GQ265640
Hymenoptera_Vespidae			
Lepidoptera_Noctuidae	GQ265618		GQ265644
Lepidoptera_Nymphalidae	KC996757	Genome	EU675861
Lepidoptera_Papilionidae	JQ787202		AK403052
Lepidoptera_Saturniidae	NM_001047060	DQ202313	NM_001126258
Mantophasmatodea_Austrophasmatidae			
Mecoptera_Bittacidae			
Mecoptera_Boreidae			GQ265637
Mecoptera_Nannochoristidae	GQ265619	GQ265631	GQ265645
Mecoptera_Panorpidae	GQ265620	GQ265632	GQ265646
Megaloptera_Corydalidae	GQ265623		GQ265648
Megaloptera_Sialidae			
Neuroptera_Chrysopidae			
Neuroptera_Polystoechotidae	GQ265616	GQ265629	GQ265642
Odonata_Calopterygidae			
Odonata_Epiophlebiidae			
Orthoptera_Acrididae			

Orthoptera_Gryllidae	GQ887060		GQ887922
Orthoptera_Tettigoniidae			
Phasmatodea_Phasmatidae			
Phthiraptera_Pediculidae		XM_002432183	XM_002426257
Plecoptera_Perlidae			
Protura_Acerentomidae			
Protura_Berberentomidae			
Psocoptera_Psocidae			
Raphidioptera_Inocelliidae			
Raphidioptera_Raphidiidae	GQ265622		
Siphonaptera_Hystrichopsyllidae			
Siphonaptera_Pulicidae	GQ265609	GQ265625	GQ265636
Strepsiptera_Mengenillidae	Genome	KC177049	Genome
Strepsiptera_Myrmecolacidae			
Thysanoptera_Phlaeothripidae			
Thysanoptera_Thripidae	GQ265614		GQ265641
Trichoptera_Hydropsychidae	GQ265617	GQ265630	GQ265643
Trichoptera_Stenopsychidae			
Zoraptera_Zorotypidae			
Zygentoma_Lepismatidae	GQ887068		GQ887929
Zygentoma_Lepismatidae2			
Zygentoma_Nicoletiidae	GQ887099		GQ887957

Ixodida_Ixodoidea	XM_002410209	XM_002435805	XM_002411260
Opiliones_Sclerosomatidae			
Anostraca_Thamnocephalidae			
Cladocera_Daphniidae	NCBI_GNO_240154	NCBI_GNO_330603	hxNCBI_GNO_9034
Cyclopoida_Cyclopidae	GQ887057		GQ887919
Decapoda_Cambaridae			HQ414580
Nectiopoda_Speleonectidae	EU020895		
Notostraca_Triopsidae	EU020897		EU020353
Podocopida_Cyprididae	EU020893		EU020351
Stomatopoda_Squillidae			
Chilopoda_Scutigeridae	GQ887109		GQ887964

Taxon name	*DPD1*	*RPB1*	*RPB2*
Archaeognatha_Machilidae	AB598694	AB596893	AB597584
Archaeognatha_Machilidae2	AB598695	AB596894	AB597585
Coleoptera_Cantharidae		EU677580	
Coleoptera_Carabidae		EU677589	
Coleoptera_Cerambycidae		GQ265664	
Coleoptera_Cupedidae		EU677579	
Coleoptera_Dytiscidae	AB598730	AB596929	AB597620
Coleoptera_Hydroscaphidae		HM156727	
Coleoptera_Lampyridae	AB598731	AB596930	AB597621

Coleoptera_Tenebrionidae	XM_008201381	XM_008196951	XM_969560
Collembola_Bourletiellidae	AB811988	AB812002	AB812016
Collembola_Isotomidae	AB811990	AB812004	AB812018
Collembola_Tomoceridae	AB811989	AB812003	AB812017
Dermaptera_Anisolabididae	AB598711	AB596910	AB597601
Dermaptera_Forficulidae	AB598712	AB596911	AB597602
Dictyoptera_Blattellidae	AB598715	AB596914	AB597605
Dictyoptera_Mantidae	AB598714	AB596913	AB597604
Dictyoptera_Rhinotermitidae	AB598716	AB596915	AB597606
Diplura_Campodeidae	AB598693	AB596892	AB597583
Diplura_Campodeidae2	AB598692	AB596891	AB597582
Diplura_Japygidae	AB811991	AB812005	AB812019
Diptera_Cecidomyiidae	Genome	Genome	Genome
Diptera_Culicidae	Genome	XM_317690	XM_313416
Diptera_Drosophilidae	X88928	NM_078569	BT003265
Diptera_Muscidae			
Diptera_Tipulidae			
Embioptera_Oligotomidae	AB598704	AB596903	AB597594
Ephemeroptera_Ephemeridae	AB598701	AB596900	AB597591
Ephemeroptera_Isonychiidae	AB598702	AB596901	AB597592
Grylloblattodea_Grylloblattidae	AB598705	AB596904	AB597595
Hemiptera_Aleyrodidae	AB598721	AB596920	AB597611

Hemiptera_Aphididae	XM_001948857	XM_001943019	XM_008185197
Hemiptera_Cicadidae	AB598719	AB596918	AB597609
Hemiptera_Coreidae	AB598720	AB596919	AB597610
Hymenoptera_Apidae	XM_006569984	XM_623278	XM_006558332
Hymenoptera_Pteromalidae	XM_001606307	XM_003425605	XM_008209300
Hymenoptera_Tenthredinidae	AB598724	AB596923	AB597614
Hymenoptera_Vespidae	AB598725	AB596924	AB597615
Lepidoptera_Noctuidae		GQ265660	
Lepidoptera_Nymphalidae	Genome	Genome	Genome
Lepidoptera_Papilionidae	AB598732	AB596931	AB597622
Lepidoptera_Saturniidae	KF878074	XM_004930121	XM_004930876
Mantophasmatodea_Austrophasmatidae	AB598706	AB596905	AB597596
Mecoptera_Bittacidae		GQ265667	
Mecoptera_Boreidae		GQ265666	
Mecoptera_Nannochoristidae		GQ265661	
Mecoptera_Panorpidae	AB598734	AB596933	AB597624
Megaloptera_Corydalidae	AB598726	AB596925	AB597616
Megaloptera_Sialidae			
Neuroptera_Chrysopidae	AB598728	AB596927	AB597618
Neuroptera_Polystoechotidae		GQ265658	
Odonata_Calopterygidae	AB598700	AB596899	AB597590
Odonata_Epiophlebiidae	AB598699	AB596898	AB597589

Orthoptera_Acrididae	AB598707	AB596906	AB597597
Orthoptera_Gryllidae	AB598709	AB596908	AB597599
Orthoptera_Tettigoniidae	AB598708	AB596907	AB597598
Phasmatodea_Phasmatidae	AB598703	AB596902	AB597593
Phthiraptera_Pediculidae	XM_002429812	XM_002426633	XM_002425468
Plecoptera_Perlidae	AB598710	AB596909	AB597600
Protura_Acerentomidae	AB811987	AB812001	AB812015
Protura_Berberentomidae	AB811986	AB812000	AB812014
Psocoptera_Psocidae	AB598723	AB596922	AB597613
Raphidioptera_Inocelliidae	AB598727	AB596926	AB597617
Raphidioptera_Raphidiidae			
Siphonaptera_Hystrichopsyllidae		GQ265669	
Siphonaptera_Pulicidae	AB598735	AB596934	AB597625
Strepsiptera_Mengenillidae	Genome	GQ265651	Genome
Strepsiptera_Myrmecolacidae	AB598729	AB596928	AB597619
Thysanoptera_Phlaeothripidae	AB598718	AB596917	AB597608
Thysanoptera_Thripidae	AB598717	AB596916	AB597607
Trichoptera_Hydropsychidae		GQ265659	
Trichoptera_Stenopsychidae	AB598733	AB596932	AB597623
Zoraptera_Zorotypidae	AB598713	AB596912	AB597603
Zygentoma_Lepismatidae	AB598697	AB596896	AB597587
Zygentoma_Lepismatidae2	AB598698	AB596897	AB597588

Zygentoma_Nicoletiidae	AB598696	AB596895	AB597586
Ixodida_Ixodoidea	XM_002399417	XM_002407458	XM_002416438
Opiliones_Sclerosomatidae	AB811978	AB811992	AB812006
Anostraca_Thamnocephalidae	AB811985	AB811999	AB812013
Cladocera_Daphniidae	AB811983	AB811997	AB812011
Cyclopoida_Cyclopidae	AB811980	AB811994	AB812008
Decapoda_Cambaridae	AB811982	AB811996	AB812010
Nectiopoda_Speleonectidae		AF139008	EU020850
Notostraca_Triopsidae	AB811984	AB811998	AB812012
Podocopida_Cyprididae		AF138997	EU020848
Stomatopoda_Squillidae	AB811981	AB811995	AB812009
Chilopoda_Scutigeridae	AB811979	AB811993	AB812007

Supplementary Table 2. Best-fitting models and partitions identified for the maximum likelihood phylogenetic analysis of nucleotide sequence data using RAXML.

No.	Genes	Best-fitting model	Sites in partition
1	*18S*, *28S*	SYM+I+G	1–1623
2	*EF1a*-codonPos1, *SNFC*-codonPos1	GTR+I+G	1624–2317\3, 3868–4036\3
3	*FLa*-codonPos2	TVMef+I+G	1625–2318\3
4	*EF1a*-codonPos3	TVM+G	1626–2319\3
5	*AATS*-codonPos1, *CAD*-codonPos1, *PGD*-codonPos1, *TPI*-codonPos1	SYM+I+G	2320–2905\3, 2908–3166\3, 3169–3865\3, 4039–4459\3
6	*AATS*-codonPos2, *CAD*-codonPos2, *PGD*-codonPos2, *SNF*-codonPos2, *TPI*-codonPos2	GTR+I+G	2321–2906\3, 2909–3167\3, 3170–3866\3, 3869–4037\3, 4040–4460\3
7	*CAD*-codonPos3, *SNF*-codonPos3, *TPI*-codonPos3	TIM+I+G	2321–2906\3, 2909–3167\3, 3170–3866\3, 3869–4037\3, 4040–4460\3

8	*AATS*-codonPos3	TrN+I+G	2910–3168\3
9	*PGD*-codonPos3	GTR+I+G	3171–3867\3

Supplementary Table 3. Best-fitting models and partitions identified for the BEAST analysis of amino-acid sequence data.

No.	Genes	Best-fitting model	Sites in partition
1	*EF1a*	Dayhoff+I+G	1624–1856
2	*CAD*	CpREV+I+G	1857–2051
3	*AATS, SNF*	WAG+G	2052–2139, 2387–2443
4	*DPD1, PGD, TPI*	WAG+I+G	2140–2386, 2444-2575, 2567–3349
5	*RPB1*	JTT+I+G	3450–4849
6	*RPB2*	JTT+I+G	4850–5975

Supplementary Table 4. Data on extant hexapod clades. Including percentage of herbivorous species (% Herb.) from estimates from this study, Grimaldi and Engel[1] (GE) and Hendrix[2] (H), presence (1) and absence (0) of wings and holometaboly, and ln-transformed species richness.

Order	% Herb. this study	% Herb. GE	% Herb. H	Wings	Holometaboly	Ln-species
Collembola	1	0	2.5	0	0	9.0074
Diplura	0	0	0	0	0	6.8824
Protura	0	0	0	0	0	6.7044
Diptera	28	30	25	1	1	11.9626
Mecoptera	0	0	0	1	1	5.9915
Siphonaptera	0	0	0	0	1	7.6411
Trichoptera	0	0	0	1	1	9.5852
Lepidoptera	100	99	98	1	1	11.9730
Strepsiptera	0	0	0	1	1	6.4184
Coleoptera	26	35	47	1	1	12.8726
Raphidioptera	0	0	0	1	1	5.2149
Neuroptera	0	0	0	1	1	8.6067
Megaloptera	0	0	0	1	1	5.8833
Hymenoptera	7	12	10	1	1	11.9388
Hemiptera	78	90	79	1	0	11.5345
Thysanoptera	68	40	99	1	0	8.6891

Pscocodea	0	0	0	1	0	9.2823
Plecoptera	0	0	0	1	0	8.2196
Dermaptera	0	0	0	1	0	7.5668
Embioptera	0	0	0	1	0	6.1247
Phasmatodea	100	100	100	1	0	8.0216
Grylloblattodea	0	0	0	0	0	3.4657
Mantophasmatodea	0	0	0	0	0	2.8332
Orthoptera	94	95	90	1	0	10.0787
Blattodea	0	0	0	1	0	8.9319
Mantodea	0	0	0	1	0	7.7936
Zoroptera	0	0	0	1	0	3.5835
Odonata	0	0	0	1	0	8.7065
Ephemeroptera	0	0	0	1	0	8.0469
Zygentoma	0	0	0	0	0	6.3172
Archaeognatha	0	0	0	0	0	6.2265

Supplementary Table 5. Data on extant hexapod clades based on the tree of Misof and colleagues. Including species richness, stem age (Ma), and diversification rates (div.; in species per million years) with relative extinction fractions of e=0, 0.5, and 0.9, with stem ages and rates based on the time-calibrated tree estimated by Misof and colleagues[3]. See Supplementary Table 4 for other data on these clades.

	Order	Species	Stem age	div. e=0	div. e=0.5	div. e=0.9
1	Protura	816	430.0653	0.0156	0.0140	0.0102
2	Collembola	8163	430.0653	0.0209	0.0193	0.0156
3	Diplura	975	461.5803	0.0149	0.0134	0.0099
4	Archaeognatha	506	440.3387	0.0141	0.0126	0.0089
5	Zygentoma	554	420.5491	0.0150	0.0134	0.0096
6	Ephemeroptera	3124	362.4544	0.0222	0.0203	0.0158
7	Odonata	6042	362.4544	0.0240	0.0221	0.0177
8	Thysanoptera	5938	339.1276	0.0256	0.0236	0.0188
9	Hemiptera	102183	339.1276	0.0340	0.0320	0.0272
10	Psocodea	10746	361.5322	0.0257	0.0238	0.0193
11	Hymenoptera	153088	344.6798	0.0346	0.0326	0.0280
12	Coleoptera	389487	286.4729	0.0449	0.0425	0.0369
13	Strepsiptera	613	286.4729	0.0224	0.0200	0.0144
14	Neuroptera	5468	259.0341	0.0332	0.0306	0.0243
15	Megaloptera	359	259.0341	0.0227	0.0200	0.0138
16	Raphidioptera	184	276.2575	0.0189	0.0164	0.0106

17	Diptera	156774	242.7428	0.0493	0.0464	0.0398
18	Siphonaptera	2082	167.549	0.0189	0.0164	0.0106
19	Mecoptera	400	167.549	0.0358	0.0316	0.0220
20	Lepidoptera	158423	207.2007	0.0578	0.0544	0.0467
21	Trichoptera	14548	207.2007	0.0463	0.0429	0.0351
22	Dermaptera	1933	168.5234	0.0449	0.0408	0.0312
23	Zoroptera	36	168.5234	0.0213	0.0173	0.0078
24	Plecoptera	3713	269.1151	0.0305	0.0280	0.0220
25	Mantodea	2425	197.3197	0.0395	0.0360	0.0278
26	Blattodea	7570	197.3197	0.0453	0.0418	0.0336
27	Embioptera	457	164.2213	0.0373	0.0331	0.0233
28	Phasmatodea	3046	164.2213	0.0488	0.0446	0.0348
29	Mantophasmatodea	17	152.6693	0.0186	0.0144	0.0039
30	Grylloblattodea	32	152.6693	0.0227	0.0184	0.0078
31	Orthoptera	23830	247.8446	0.0407	0.0379	0.0314

Supplementary Table 6. Data on extant hexapod clades based on the tree of Rainford and colleagues. Including species richness, stem age (Ma), diversification rates (div.; in species per million years) with relative extinction fractions of e=0, 0.5, and 0.9, with stem ages and rates based on the tree of Rainford and colleagues[4].

	Order	Species	Stem age	div. e=0	div. e=0.5	div. e=0.9
1	Diplura	975	440.9346	0.01560875	0.01403908	0.01040753
2	Protura	816	440.9346	0.01520501	0.01363579	0.01000783
3	Collembola	8163	463.9877	0.01941294	0.01791932	0.01445272
4	Archaeognatha	506	462.318	0.01346808	0.01197307	0.00852569
5	Zygentoma	554	449.1971	0.01406324	0.01252417	0.00897311
6	Odonata	6042	373.2606	0.02332550	0.02146894	0.01716065
7	Ephemeroptera	3124	373.2606	0.02155831	0.01970217	0.01539718
8	Dermaptera	1933	356.6554	0.02121608	0.01927406	0.01477305
9	Plecoptera	3713	356.6554	0.02304632	0.02110361	0.01659706
10	Orthoptera	23830	387.3472	0.02601981	0.02423045	0.02007629
11	Grylloblattodea	32	220.4915	0.01571823	0.01271414	0.00639928
12	Mantophasmatodea	17	220.4915	0.01284954	0.00996512	0.00433355
13	Embioptera	457	259.6484	0.02358837	0.02092723	0.01479539
14	Phasmatodea	3046	259.6484	0.03089403	0.02822573	0.02203730
15	Zoraptera	36	340.5617	0.01052238	0.00856752	0.00441646
16	Blattodea	7570	307.2351	0.02907203	0.02681638	0.02158136
17	Mantodea	2425	307.2351	0.02536685	0.02311211	0.01788437

18	Pscocodea	10746	416.6025	0.02228092	0.02061734	0.01675588
19	Thysanoptera	5938	404.6113	0.02147525	0.01976254	0.01578813
20	Hemiptera	102183	404.6113	0.02850766	0.02679456	0.02281702
21	Hymenoptera	153088	389.6906	0.03063653	0.02885784	0.02472793
22	Raphidioptera	184	300.3543	0.01736261	0.01507289	0.00985538
23	Megaloptera	359	284.8324	0.02065538	0.01823162	0.01265831
24	Neuroptera	5468	284.8324	0.03021661	0.02778372	0.02213838
25	Strepsiptera	613	309.3165	0.02075015	0.01851452	0.01335317
26	Coleoptera	389487	309.3165	0.04161623	0.03937534	0.03417220
27	Mecoptera	400	260.7736	0.02297573	0.02032727	0.01423123
28	Siphonaptera	2082	260.7736	0.02930160	0.02664540	0.02048832
29	Diptera	156774	316.8174	0.03775853	0.03557071	0.03049085
30	Trichoptera	14548	302.4121	0.03169585	0.02940402	0.02408383
31	Lepidoptera	158423	302.4121	0.03959175	0.03729971	0.03197787

Supplementary Table 7. Data on extant hexapod clades based on the tree generated in this study. Including species richness, stem age (Ma), and diversification rates (div.; in species per million years) with relative extinction fractions of e=0, 0.5, and 0.9, with ages and rates based on the tree generated in this study.

	Order	Species	Stem age	div. e=0	div. e=0.5	div. e=0.9
1	Collembola	8163	539.4136	0.0167	0.0154	0.0124
2	Diplura	975	473.9179	0.0145	0.0131	0.0097
3	Protura	816	473.9179	0.0141	0.0127	0.0093
4	Diptera	156774	278.5465	0.0429	0.0405	0.0347
5	Mecoptera	400	223.093	0.0269	0.0238	0.0165
6	Siphonaptera	2082	223.093	0.0343	0.0311	0.0239
7	Trichoptera	14548	193.4289	0.0496	0.0460	0.0377
8	Lepidoptera	158423	193.4289	0.0619	0.0583	0.0500
9	Strepsiptera	613	309.116	0.0208	0.0185	0.0133
10	Coleoptera	389487	309.116	0.0416	0.0394	0.0342
11	Raphidioptera	184	192.0828	0.0271	0.0236	0.0152
12	Neuroptera	5468	192.0828	0.0448	0.0412	0.0328
13	Megaloptera	359	245.4276	0.0240	0.0212	0.0146
14	Hymenoptera	153088	375.44	0.0318	0.0300	0.0257
15	Hemiptera	102183	255.7705	0.0451	0.0424	0.0361
16	Thysanoptera	5938	255.7705	0.0340	0.0313	0.0250
17	Pscocodea	10746	326.6535	0.0284	0.0263	0.0214

18	Plecoptera	3713	255.2009	0.0322	0.0295	0.0232
19	Dermaptera	1933	255.2009	0.0297	0.0269	0.0206
20	Embioptera	457	200.9888	0.0305	0.0270	0.0190
21	Phasmatodea	3046	200.9888	0.0399	0.0365	0.0285
22	Grylloblattodea	32	178.5282	0.0194	0.0157	0.0067
23	Mantophasmatodea	17	178.5282	0.0159	0.0123	0.0033
24	Orthoptera	23830	293.6015	0.0343	0.0320	0.0265
25	Blattodea	7570	210.3753	0.0425	0.0392	0.0315
26	Mantodea	2425	210.3753	0.0370	0.0338	0.0261
27	Zoroptera	36	260.8762	0.0137	0.0112	0.0050
28	Odonata	6042	344.1335	0.0253	0.0233	0.0186
29	Ephemeroptera	3124	344.1335	0.0234	0.0214	0.0167
30	Zygentoma	554	456.3689	0.0138	0.0123	0.0088
31	Archaeognatha	506	532.2934	0.0117	0.0104	0.0074

Supplementary Table 8. Full results of PGLS analyses using the tree of hexapod clades generated by Misof and colleagues[4]. Diversification rates were multiplied by 1000 when estimating the AIC of each model to ensure non-negative AIC values.

Variables	r^2	*P*	AIC
Diversification rate (e=0) ~ herbivory	0.2721	0.0003	236.5808
Diversification rate (e=0.5) ~ herbivory	0.2854	0.0002	233.7007
Diversification rate (e=0.9) ~ herbivory	0.3056	0.0001	229.0834
Ln-species ~ herbivory	0.2520	0.0006	140.3157
Diversification rate (e=0) ~ wings	0.3995	<0.0001	230.6167
Diversification rate (e=0.5) ~ wings	0.4031	<0.0001	228.1224
Diversification rate (e=0.9) ~ wings	0.3960	<0.0001	224.7614
Ln-species ~ wings	0.1817	0.0049	142.4793
Diversification rate (e=0) ~ holometaboly	0.0748	0.1139	244.0173
Diversification rate (e=0.5) ~ holometaboly	0.0788	0.1014	241.5743
Diversification rate (e=0.9) ~ holometaboly	0.0888	0.0755	237.5065
Ln-species ~ holometaboly	0.0845	0.0859	145.9590
Ln-species ~ diversification rate (e=0)	0.6196	<0.0001	122.7615
Ln-species ~ diversification rate (e=0.5)	0.6652	<0.0001	118.8005
Ln-species ~ diversification rate (e=0.9)	0.7658	<0.0001	107.7294
Ln-species ~ age	0.0418	0.2974	147.3709
Diversification rate (e=0) ~ herb. + wings	0.5242	<0.0001	225.4016
Diversification rate (e=0.5) ~ herb. + wings	0.5366	<0.0001	222.2741

Diversification rate (e=0.9) ~ herb. + wings	0.5454	<0.0001	217.9511
Div. rate (e=0) ~ herb. + wings + holometaboly	0.5424	<0.0001	226.0930
Div. rate (e=0.5) ~ herb. + wings + holometaboly	0.5601	<0.0001	222.6616
Div. rate (e=0.9) ~ herb. + wings + holometaboly	0.5764	<0.0001	217.7636

Supplementary Table 9. Full results of PGLS analyses using the tree of hexapod clades generated by Rainford and colleagues[4]. The relationship between Ln-species and age is from ordinary least-squares regression (PGLS failed). Diversification rates were multiplied by 1000 when estimating the AIC of each model to ensure non-negative AIC values.

Variables	r^2	*P*	AIC
Diversification rate (e=0) ~ herbivory	0.2560	0.0005	209.2678
Diversification rate (e=0.5) ~ herbivory	0.2606	0.0004	208.7478
Diversification rate (e=0.9) ~ herbivory	0.2692	0.0003	207.1512
Ln-species ~ herbivory	0.2710	0.0003	140.0721
Diversification rate (e=0) ~ wings	0.2673	0.0004	209.1650
Diversification rate (e=0.5) ~ wings	0.2668	0.0004	208.5151
Diversification rate (e=0.9) ~ wings	0.2558	0.0005	207.1406
Ln-species ~ wings	0.1817	0.0049	142.4792
Diversification rate (e=0) ~ holometaboly	0.2872	0.0002	208.3112
Diversification rate (e=0.5) ~ holometaboly	0.2719	0.0004	208.2960
Diversification rate (e=0.9) ~ holometaboly	0.2330	0.0010	208.0750
Ln-species ~ holometaboly	0.0845	0.0859	145.9588
Ln-species ~ diversification rate (e=0)	0.8036	<0.0001	99.5917
Ln-species ~ diversification rate (e=0.5)	0.8352	<0.0001	94.1533
Ln-species ~ diversification rate (e=0.9)	0.8945	<0.0001	80.3290
Ln-species ~ age	0.0691	0.1532	148.4754

Diversification rate (e=0) ~ herb + holometaboly	0.5222	<0.0001	197.9129
Diversification rate (e=0.5) ~ herb. + holometaboly	0.5126	<0.0001	197.8569
Diversification rate (e=0.9) ~ herb. + holometaboly	0.4840	<0.0001	197.7849
Div. rate (e=0) ~ herb. + wings + holometaboly	0.5802	<0.0001	195.9012
Div. rate (e=0.5) ~ herb. + wings + holometaboly	0.5715	<0.0001	195.8600
Div. rate (e=0.9) ~ herb. + wings + holometaboly	0.5414	<0.0001	196.1284

Supplementary Table 10. Full results of PGLS analyses using the tree of hexapod clades generated in this study. Diversification rates were multiplied by 1000 when estimating the AIC of each model to ensure non-negative AIC values.

Variables	r^2	*P*	AIC
Diversification rate (e=0) ~ herbivory	0.2902	0.0002	231.2431
Diversification rate (e=0.5) ~ herbivory	0.3010	0.0001	229.4797
Diversification rate (e=0.9) ~ herbivory	0.3177	<0.0001	226.5232
Ln-species ~ herbivory	0.2495	0.0006	140.1257
Diversification rate (e=0) ~ holometaboly	0.0859	0.0823	239.5718
Diversification rate (e=0.5) ~ holometaboly	0.0942	0.0647	238.1766
Diversification rate (e=0.9) ~ holometaboly	0.1656	0.0079	235.0378
Ln-species ~ holometaboly	0.0845	0.0859	145.9590
Diversification rate (e=0) ~ wings	0.3619	<0.0001	232.8054
Diversification rate (e=0.5) ~ wings	0.3596	<0.0001	230.8036
Diversification rate (e=0.9) ~ wings	0.3429	<0.0001	227.6331
Ln-species ~ wing	0.1817	0.0049	142.4793
Ln-species ~ diversification rate (e=0)	0.6313	<0.0001	118.7261
Ln-species ~ diversification rate (e=0.5)	0.6741	<0.0001	114.9044
Ln-species ~ diversification rate (e=0.9)	0.7708	<0.0001	103.9894
Ln-species ~ age	0.0141	0.6652	148.5308
Diversification rate (e=0) ~ herb. + wings	0.5148	<0.0001	226.3127
Diversification rate (e=0.5) ~ herb. + wings	0.5197	<0.0001	223.8834

Diversification rate (e=0.9) ~ herb. + wings	0.5157	<0.0001	220.1742
Div. rate (e=0) ~ herb. + wings + holometaboly	0.6161	<0.0001	221.0579
Div. rate (e=0.5) ~ herb. + wings + holometaboly	0.6197	<0.0001	218.6464
Div. rate (e=0.9) ~ herb. + wings + holometaboly	0.6101	<0.0001	215.4544

Supplementary Table 11. Data for analyses of Coleoptera. Including percent herbivory (see text for explanation), species richness, stem-group ages (Ma), estimated diversification rates (in species per million years; using three values for relative extinction rates, e=0, 0.5, and 0.9), and ln-transformed species richness. Clade names, presence of herbivory, and estimates of richness follow Hunt and colleagues[5] (see Methods for modifications).

Clade	Herb.	Species	Stem age	div. e=0	div. e=0.5	div. e=0.9	Ln-species
CPCTeAn_Anthicinae	0	2900	130.7132	0.0610	0.0557	0.0434	7.9720
CPCTeMe_Lyttinae	0	1100	64.3884	0.1088	0.0980	0.0731	7.0030
CPCTeMe_Meloinae	0	190	64.3884	0.0815	0.0708	0.0464	5.2470
CPCTeMe_Nemognathinae	0	515	98.9682	0.0631	0.0561	0.0400	6.2440
CPCTeAn_Eurygeniinae	0	125	152.6436	0.0316	0.0271	0.0170	4.8280
CPCTeCi_Ciinae	0	550	169.3525	0.0373	0.0332	0.0238	6.3100
CPCTeTe_Phrenapatinae	0	600	80.0515	0.0799	0.0713	0.0513	6.3970
CPCTeTe_Diaperinae	0	1800	80.0515	0.0936	0.0850	0.0649	7.4960
CPCTeTe_Alleculinae	0	1000	99.8722	0.0692	0.0622	0.0462	6.9080
CPCTeTe_Coelometopinae	0	600	99.8722	0.0641	0.0571	0.0411	6.3970
CPCTeTe_Tenebrioninae	0	10000	146.5915	0.0628	0.0581	0.0471	9.2100
CPCTeZo_Usechinae	0	7	14.2333	0.1367	0.0974	0.0330	1.9460
CPCTeZo_Zopherinae	0	120	14.2333	0.3364	0.2882	0.1797	4.7870
CPCTePy_Agnathinae	0	1	21.8007	0.0000	0.0000	0.0000	0.0000
CPCTe_Monommatidae	0	300	21.8007	0.2616	0.2300	0.1574	5.7040
CPCTeTe_Pimeliinae	0	10000	80.0678	0.1150	0.1064	0.0863	9.2100
CPCTeSa_Prostominiinae	0	10	13.0401	0.1766	0.1307	0.0492	2.3030
CPCTeSa_Trogocryptinae	0	10	13.0401	0.1766	0.1307	0.0492	2.3030

CPCTeMy_Mycetophaginae	0	200	44.3375	0.1195	0.1040	0.0686	5.2980
CPCTeMea_Eustrophinae	0	100	82.389	0.0559	0.0476	0.0290	4.6050
CPCTeSt_Cephaloinae	0	20	82.389	0.0364	0.0285	0.0129	2.9960
CPCTeZo_Colydiinae	0	1000	104.2758	0.0662	0.0596	0.0442	6.9080
CPCTeSa_Othniinae	0	50	104.2758	0.0375	0.0311	0.0170	3.9120
CPCTe_Boridae	0	4	108.2091	0.0128	0.0085	0.0024	1.3860
CPCTeOe_Oedemerinae	33	1500	108.2091	0.0676	0.0612	0.0464	7.3130
CPCTeSc_Anaspidinae	0	300	110.482	0.0516	0.0454	0.0311	5.7040
CPCTeMea_Osphyinae	0	50	110.482	0.0354	0.0293	0.0161	3.9120
CPCTeSc_Scraptiinae	0	100	131.8224	0.0349	0.0298	0.0181	4.6050
CPCTeMea_Melandryinae	0	350	87.9063	0.0666	0.0588	0.0407	5.8580
CPCTeMea_Hypulinae	0	30	87.9063	0.0387	0.0312	0.0155	3.4010
CPCTeTer_Penthinae	0	11	103.0641	0.0233	0.0174	0.0067	2.3980
CPCTe_Trictenotomidae	0	15	126.0709	0.0215	0.0165	0.0069	2.7080
CPCTe_Pythidae	0	20	105.671	0.0283	0.0223	0.0101	2.9960
CPCTePy_Pedilinae	0	50	78.1573	0.0501	0.0414	0.0227	3.9120
CPCTePy_Pyrochroinae	0	150	78.1573	0.0641	0.0553	0.0354	5.0110
CPCTeSa_Inopeplinae	0	80	117.8867	0.0372	0.0314	0.0185	4.3820
CPCTeSa_Aegialitinae	0	20	91.1004	0.0329	0.0258	0.0117	2.9960
CPCTeSa_Salpinginae	0	150	91.1004	0.0550	0.0475	0.0304	5.0110
CPCTeMea_Hallomeninae	0	55	135.4828	0.0296	0.0246	0.0137	4.0070
CPCTeTer_Tetratominae	0	22	107.0433	0.0289	0.0228	0.0106	3.0910
CPCTe_Perimylopidae	0	19	107.0433	0.0275	0.0215	0.0096	2.9440
CPCTeAn_Ischaliinae	0	50	118.2912	0.0331	0.0274	0.0150	3.9120
CPCTe_Aderidae	33	1000	118.2912	0.0584	0.0525	0.0390	6.9080
CPCTeTe_Lagriinae	50	1600	145.8319	0.0506	0.0458	0.0348	7.3780
CPCTeRi_Pelecotominae	0	75	146.7106	0.0294	0.0248	0.0145	4.3170
CPCTeRi_Rhipidiinae	0	100	146.7106	0.0314	0.0267	0.0163	4.6050

CPCTeRi_Rhipiphorinae	0	220	169.6317	0.0318	0.0277	0.0185	5.3940
CPCLyLy_Hylecoetinae	0	5	85.1197	0.0189	0.0129	0.0040	1.6090
CPCLyLy_Melittommatinae	0	25	85.1197	0.0378	0.0301	0.0144	3.2190
CPCTeMo_Mordellinae	33	1500	140.5434	0.0520	0.0471	0.0357	7.3130
CPCLyLy_Lymexylinae	0	24	206.4659	0.0154	0.0122	0.0058	3.1780
CPCCuSpSp_Sphindus	0	60	210.7121	0.0194	0.0162	0.0092	4.0940
CPCClCl_Clerinae	0	1600	77.4835	0.0952	0.0863	0.0656	7.3780
CPCClCl_Hydnocerinae	0	600	77.4835	0.0826	0.0736	0.0530	6.3970
CPCClCl_Korynetinae	0	150	93.3763	0.0537	0.0463	0.0296	5.0110
CPCClCl_Enopliinae	0	650	93.3763	0.0694	0.0620	0.0449	6.4770
CPCClCl_Tillinae	0	600	124.4142	0.0514	0.0459	0.0330	6.3970
CPCClMe_Prionocerinae	0	150	102.7581	0.0488	0.0421	0.0269	5.0110
CPCClMe_Malachiinae	0	4000	48.9459	0.1695	0.1553	0.1225	8.2940
CPCClMe_Dasytinae	0	750	48.9459	0.1353	0.1211	0.0885	6.6200
CPCClMe_Melyrinae	0	300	80.9905	0.0704	0.0619	0.0424	5.7040
CPCClMe_Rhadalinae	0	300	120.3616	0.0474	0.0417	0.0285	5.7040
CPCClTr_Peltinae	0	104	140.4998	0.0331	0.0282	0.0173	4.6440
CPCClTr_Lophocaterinae	0	20	162	0.0185	0.0145	0.0066	2.9960
CPCCu_Byturidae	100	16	54.505	0.0509	0.0393	0.0168	2.7730
CPCCu_Biphyllidae	0	195	54.505	0.0967	0.0841	0.0553	5.2730
CPCClPh_Phloiophilidae	0	1	136.0666	0.0000	0.0000	0.0000	0.0000
CPCClTr_Trogossitinae	0	392	190.3696	0.0314	0.0277	0.0194	5.9710
CPCCuCo_Scymninae	0	3500	81.16	0.1005	0.0920	0.0722	8.1610
CPCCuCo_Chilocorinae	0	200	81.16	0.0653	0.0568	0.0375	5.2980
CPCCuCo_Coccidulinae	0	200	101.0736	0.0524	0.0456	0.0301	5.2980
CPCCuCo_Epilachninae	100	1051	114.009	0.0610	0.0550	0.0409	6.9570
CPCCuCo_Coccinellinae	0	850	131.5029	0.0513	0.0460	0.0339	6.7450
CPCCu_Alexiidae	0	32	158.1722	0.0219	0.0177	0.0089	3.4660

CPCCuEn_Anamorphinae	0	500	158.1722	0.0393	0.0349	0.0248	6.2150
CPCCuEn_Leiestinae	0	100	139.0898	0.0331	0.0282	0.0172	4.6050
CPCCuEn_Holoparamecinae	0	50	120.4964	0.0325	0.0269	0.0147	3.9120
CPCCuCoy_Corylophinae	0	250	66.8381	0.0826	0.0723	0.0487	5.5210
CPCCuCoy_Sericoderinae	0	30	66.8381	0.0509	0.0410	0.0204	3.4010
CPCCuEn_Endomychinae	0	100	58.4501	0.0788	0.0671	0.0409	4.6050
CPCCuEn_Lycoperdininae	0	650	58.4501	0.1108	0.0990	0.0717	6.4770
CPCCuLah_Corticariinae	50	850	110.6391	0.0610	0.0547	0.0402	6.7450
CPCCuLah_Latridiinae	0	200	110.6391	0.0479	0.0417	0.0275	5.2980
CPCCu_Discolomatidae	0	400	98.8068	0.0606	0.0536	0.0376	5.9910
CPCCuCe_Euxestinae	0	60	59.2905	0.0691	0.0576	0.0326	4.0940
CPCCuBo_Teredinae	0	30	59.2905	0.0574	0.0462	0.0230	3.4010
CPCCuBo_Anommatinae	0	50	98.8068	0.0396	0.0328	0.0180	3.9120
CPCCuBo_Xylariophilinae	0	3	140.7629	0.0078	0.0049	0.0013	1.0990
CPCCuCe_Ceryloninae	0	740	157.5262	0.0419	0.0375	0.0274	6.6070
CPCCuPha_Phalacrinae	50	504	107.2632	0.0580	0.0516	0.0367	6.2230
CPCCu_Laemophloeidae	0	400	34.5329	0.1735	0.1535	0.1075	5.9910
CPCCu_Propalticidae	0	35	34.5329	0.1030	0.0837	0.0429	3.5550
CPCCuCr_Cryptophaginae	0	400	161.411	0.0371	0.0328	0.0230	5.9910
CPCCu_Passandridae	0	105	75.2618	0.0618	0.0528	0.0323	4.6540
CPCCu_Cucujidae	0	20	75.2618	0.0398	0.0312	0.0141	2.9960
CPCCuEr_Tritominae	0	1500	68.388	0.1069	0.0968	0.0734	7.3130
CPCCuLag_Xenoscelinae	0	100	40.31222	0.1142	0.0973	0.0593	4.6050
CPCCuLag_Languriinae	100	800	40.31222	0.1658	0.1487	0.1090	6.6850
CPCCuEr_Megalodacninae	0	100	68.388	0.0673	0.0573	0.0349	4.6050
CPCCuEr_Encaustinae	0	100	73.2834	0.0628	0.0535	0.0326	4.6050
CPCCuEr_Dacninae	0	150	73.2834	0.0684	0.0590	0.0377	5.0110
CPCCuLag_Toraminae	0	50	94.185	0.0415	0.0344	0.0188	3.9120

CPCCuLag_Cryptophilinae	0	50	103.2396	0.0379	0.0314	0.0172	3.9120
CPCCuEr_Erotylinae	0	650	132.8852	0.0487	0.0435	0.0315	6.4770
CPCCu_Protocucujidae	0	5	65.8195	0.0245	0.0167	0.0051	1.6090
CPCCuMo_Monotominae	0	200	166.3713	0.0318	0.0277	0.0183	5.2980
CPCCuNi_Carpophilinae	0	597	115.6705	0.0553	0.0493	0.0355	6.3920
CPCCuNi_Cryptarchinae	0	227	24.6564	0.2200	0.1921	0.1282	5.4250
CPCCuNi_Cillaeinae	0	450	24.6564	0.2478	0.2198	0.1552	6.1090
CPCCuNi_Nitidulinae	0	1000	38.0055	0.1818	0.1635	0.1214	6.9080
CPCChCh_Chrysomelinae	100	2000	138.9426	0.0547	0.0497	0.0382	7.6010
CPCChCh_Galerucinae	100	15000	138.9426	0.0692	0.0642	0.0526	9.6160
CPCChCh_Lamprosmatinae	100	190	154.3149	0.0340	0.0295	0.0194	5.2470
CPCChCh_Eumolpinae	100	3200	125.1571	0.0645	0.0590	0.0461	8.0710
CPCChCh_Cryptocephalinae	50	2290	125.1571	0.0618	0.0563	0.0434	7.7360
CPCChCh_Donaciinae	100	165	132.7222	0.0385	0.0333	0.0215	5.1060
CPCChCh_Criocerinae	100	1500	132.7222	0.0551	0.0499	0.0378	7.3130
CPCChCh_Bruchinae	100	1500	159.4597	0.0459	0.0415	0.0315	7.3130
CPCChCh_Sagrinae	100	74	183.7251	0.0234	0.0197	0.0115	4.3040
CPCChCe_Vesperinae	0	50	128.3674	0.0305	0.0252	0.0138	3.9120
CPCChOr_Aulacoscelidinae	100	19	128.3674	0.0229	0.0179	0.0080	2.9440
CPCChMe_Palophaginae	100	4	128.3674	0.0108	0.0071	0.0020	1.3860
CPCChMe_Zeugophorinae	100	55	100.802	0.0398	0.0331	0.0184	4.0070
CPCChCe_Parandrinae	0	30	100.802	0.0337	0.0272	0.0135	3.4010
CPCChCe_Spondylidinae	0	100	100.2655	0.0459	0.0391	0.0238	4.6050
CPCChOr_Orsodacninae	100	10	100.2655	0.0230	0.0170	0.0064	2.3030
CPCChCe_Disteniinae	0	80	137.0928	0.0320	0.0270	0.0159	4.3820
CPCChCe_Necydalinae	0	75	124.1764	0.0348	0.0293	0.0171	4.3170
CPCChCe_Lepturinae	0	2500	124.1764	0.0630	0.0574	0.0445	7.8240
CPCChCe_Lamiinae	0	16000	148.2677	0.0653	0.0606	0.0498	9.6800

CPCChCe_Prioninae	0	1600	110.0542	0.0670	0.0607	0.0462	7.3780
CPCChCe_Cerambycinae	50	1000	111.0542	0.0622	0.0560	0.0415	6.9080
CPCCucNe_Rhinorhynchinae	100	20	53.3514	0.0562	0.0441	0.0200	2.9960
CPCCucAn_Choraginae	33	350	32.8447	0.1784	0.1573	0.1090	5.8580
CPCCucAn_Anthribinae	0	2650	32.8447	0.2400	0.2189	0.1700	7.8820
CPCCucAn_Urodontinae	100	80	53.3514	0.0821	0.0694	0.0410	4.3820
CPCCucAt_Rhynchitinae	100	1080	58.8895	0.1186	0.1069	0.0796	6.9850
CPCCucAt_Attelabinae	100	972	58.8895	0.1168	0.1051	0.0779	6.8790
CPCCucNe_Doydirhynchinae	100	15	73.5234	0.0368	0.0283	0.0119	2.7080
CPCCucBr_Cycladinae	100	38	85.0652	0.0428	0.0349	0.0182	3.6380
CPCCucCu_Scolytinae	0	5800	85.0652	0.1019	0.0937	0.0748	8.6660
CPCCucBr_Brentinae	0	1165	103.6366	0.0681	0.0614	0.0460	7.0600
CPCCucCu_Cossoninae	50	1666	67.5444	0.1098	0.0996	0.0758	7.4180
CPCCucCu_Platypodinae	0	1500	67.5444	0.1083	0.0980	0.0743	7.3130
CPCCucCu_Curculioninae	50	40000	94.9787	0.1116	0.1043	0.0873	10.5970
CPCCucCu_Dryophthorinae	33	1070	109.2307	0.0639	0.0575	0.0429	6.9750
CPCCuc_Caridae	100	5	118.6917	0.0136	0.0093	0.0028	1.6090
CPCCucCu_Brachycerinae	100	385	70.3565	0.0846	0.0748	0.0522	5.9530
CPCCucIt_Ithyceridae	100	1	67.8794	0.0000	0.0000	0.0000	0.0000
CPCCucBr_Nanophyinae	100	266	67.8794	0.0823	0.0721	0.0488	5.5830
CPCCucBr_Apioninae	100	2100	80.3158	0.0952	0.0866	0.0666	7.6500
CPCCucBe_Belinae	100	150	58.7479	0.0853	0.0736	0.0471	5.0110
CPCCucBe_Oxycoryninae	100	27	58.7479	0.0561	0.0449	0.0218	3.2960
CPCChCh_Hispinae	100	3000	125.678	0.0637	0.0582	0.0454	8.0060
CPCChCh_Cassidinae	100	3000	125.678	0.0637	0.0582	0.0454	8.0060
CPCCuSi_Silvaninae	0	300	143.9826	0.0396	0.0348	0.0238	5.7040
CPCCuSi_Brontinae	0	170	143.9826	0.0357	0.0309	0.0200	5.1360
CPEBy_Callirhipidae	0	16	129.3835	0.0214	0.0165	0.0071	2.7730

CPEByPt_Ptilodactylinae	0	500	113.4164	0.0548	0.0487	0.0346	6.2150
CPEByPt_Cladotominae	0	10	113.4164	0.0203	0.0150	0.0057	2.3030
CPEByEl_Larainae	0	130	95.4779	0.0510	0.0438	0.0276	4.8680
CPEBy_Chelonariidae	0	300	64.8082	0.0880	0.0774	0.0529	5.7040
CPEByEl_Elminae	0	1200	64.8082	0.1094	0.0987	0.0740	7.0900
CPEBy_Eulichadidae	0	23	115.34	0.0272	0.0215	0.0101	3.1350
CPEByPs_Eubrianacinae	0	58	115.34	0.0352	0.0293	0.0165	4.0600
CPEByLi_Limnichinae	0	376	137.5248	0.0431	0.0381	0.0265	5.9300
CPEBy_Dryopidae	0	280	137.5248	0.0410	0.0360	0.0245	5.6350
CPEElLa_Luciolinae	0	500	90.6581	0.0685	0.0609	0.0433	6.2150
CPEElLa_Ototretinae	0	300	90.6581	0.0629	0.0553	0.0378	5.7040
CPEElLy_Erotinae	0	400	60.1879	0.0995	0.0881	0.0617	5.9910
CPEElLy_Lycinae	0	1500	60.1879	0.1215	0.1100	0.0833	7.3130
CPEElLy_Calochrominae	0	700	74.4264	0.0880	0.0787	0.0573	6.5510
CPEElLy_Metriorrhynchinae	0	1800	87.1521	0.0860	0.0781	0.0596	7.4960
CPEElLy_Ateliinae	0	50	101.001	0.0387	0.0321	0.0176	3.9120
CPEElLy_Leptolycinae	0	50	123.985	0.0316	0.0261	0.0143	3.9120
CPEElCa_Cantharinae	0	2000	93.8014	0.0810	0.0736	0.0565	7.6010
CPEElCa_Chauliognathinae	0	300	93.8014	0.0608	0.0535	0.0366	5.7040
CPEElCa_Malthininae	0	1000	116.8999	0.0591	0.0532	0.0395	6.9080
CPEElEl_Cardiophorinae	0	800	93.6563	0.0714	0.0640	0.0469	6.6850
CPEEl_Phengodidae	0	280	93.6563	0.0602	0.0528	0.0359	5.6350
CPEElEl_Elaterinae	33	3500	73.0786	0.1117	0.1022	0.0802	8.1610
CPEElEl_Thylacosterninae	0	50	97.9246	0.0399	0.0331	0.0181	3.9120
CPEEl_Rhagophthalmidae	0	150	125.7052	0.0399	0.0344	0.0220	5.0110
CPEElElDe_Denticollis	33	1500	117.373	0.0623	0.0564	0.0427	7.3130
CPEEl_Drilidae	0	220	94.0276	0.0574	0.0500	0.0333	5.3940
CPEElEl_Agrypninae	50	2300	94.0276	0.0823	0.0750	0.0579	7.7410

CPEEl_Omalisidae	0	10	153.6325	0.0150	0.0111	0.0042	2.3030
CPEElEu_Eucneminae	0	1300	142.5245	0.0503	0.0455	0.0342	7.1700
CPEEl_Throscidae	0	152	142.5245	0.0352	0.0304	0.0195	5.0240
CPEDaDa_Dascillinae	100	80	73.1112	0.0599	0.0506	0.0299	4.3820
CPEDa_Rhipiceridae	0	57	73.1112	0.0553	0.0461	0.0258	4.0430
CPEByBy_Byrrhinae	0	280	47.5763	0.1184	0.1039	0.0707	5.6350
CPEByBy_Syncalyptinae	0	120	47.5763	0.1006	0.0862	0.0538	4.7870
CPBBo_Nosodendridae	0	70	168.2899	0.0252	0.0212	0.0123	4.2480
CPEBuBu_Agrilinae	50	3500	93.7277	0.0871	0.0797	0.0625	8.1610
CPEBuBu_Buprestinae	50	5500	63.0512	0.1366	0.1256	0.1001	8.6130
CPEBuBu_Acmaeoderinae	50	1300	63.0512	0.1137	0.1027	0.0773	7.1700
CPEBuBu_Julodinae	50	200	125.8485	0.0421	0.0366	0.0242	5.2980
CPEBuBu_Trachyinae	100	3500	142.5154	0.0573	0.0524	0.0411	8.1610
CPEBy_Heterocerinae	0	349	174.8792	0.0335	0.0295	0.0205	5.8550
CPBBoAn_Dryophilinae	0	84	101.6319	0.0436	0.0369	0.0219	4.4310
CPBBoBo_Bostrichinae	0	400	80.4633	0.0745	0.0659	0.0461	5.9910
CPBBoBo_Dinoderinae	0	50	80.4633	0.0486	0.0403	0.0221	3.9120
CPBBoBo_Lyctinae	0	90	126.4747	0.0356	0.0302	0.0181	4.5000
CPBBoAn_Mesocoelopodinae	0	369	99.3801	0.0595	0.0525	0.0365	5.9110
CPBBoAn_Ptilininae	0	62	99.3801	0.0415	0.0347	0.0197	4.1270
CPBBoAn_Dorcatominae	0	665	97.2009	0.0669	0.0598	0.0433	6.5000
CPBBoAn_Xyletininae	0	375	97.2009	0.0610	0.0539	0.0375	5.9270
CPBBoAn_Anobiinae	0	398	141.728	0.0422	0.0374	0.0262	5.9860
CPBBoAn_Gibbiinae	0	20	113.666	0.0264	0.0207	0.0094	2.9960
CPBBoAn_Ptininae	0	631	113.666	0.0567	0.0506	0.0366	6.4470
CPBBoDe_Dermestinae	0	300	114.8349	0.0497	0.0437	0.0299	5.7040
CPBBoDe_Trinodinae	0	50	114.8349	0.0341	0.0282	0.0155	3.9120
CPBBoDe_Megatominae	0	650	123.0501	0.0526	0.0470	0.0340	6.4770

CPBBoDe_Attageninae	0	220	163.1902	0.0331	0.0288	0.0192	5.3940
CPBBoDe_Orphilinae	0	10	180.7024	0.0127	0.0094	0.0036	2.3030
CPScScSc_Dynastinae	50	1619	92.197	0.0801	0.0726	0.0552	7.3900
CPScScSc_Rutelinae	100	3817	92.197	0.0895	0.0819	0.0645	8.2470
CPScScSc_Orphninae	50	156	111.5253	0.0453	0.0391	0.0251	5.0500
CPScScSc_Melolonthinae	0	10737	111.5253	0.0832	0.0770	0.0626	9.2810
CPScScSc_Cetoniinae	0	4121	140.1229	0.0594	0.0545	0.0430	8.3240
CPScSc_Glaphyridae	0	174	97.6623	0.0528	0.0458	0.0298	5.1590
CPScScOc_Ochodaeinae	0	105	97.6623	0.0477	0.0407	0.0249	4.6540
CPScScSc_Scarabaeinae	0	5317	94.7729	0.0905	0.0832	0.0662	8.5790
CPScSc_Glaresidae	0	50	94.7729	0.0413	0.0342	0.0187	3.9120
CPScSc_Hybosoridae	0	217	130.6903	0.0412	0.0359	0.0239	5.3800
CPScScSc_Aphodiinae	0	3187	156.1482	0.0517	0.0472	0.0369	8.0670
CPScScLu_Dorcinae	0	770	78.9589	0.0842	0.0754	0.0552	6.6460
CPScScLu_Lucaninae	0	281	78.9589	0.0714	0.0627	0.0426	5.6380
CPScScLu_Aesalinae	0	70	89.0387	0.0477	0.0401	0.0232	4.2480
CPScScLu_Nicaginae	0	50	89.0387	0.0439	0.0364	0.0199	3.9120
CPScSc_Ceratocanthidae	0	307	112.1593	0.0511	0.0449	0.0308	5.7270
CPScScGe_Bolboceratinae	0	300	123.0413	0.0464	0.0408	0.0279	5.7040
CPScSc_Trogidae	0	330	121.3588	0.0478	0.0421	0.0290	5.7990
CPScScPa_Aulacocyclinae	0	35	38.0061	0.0935	0.0761	0.0390	3.5550
CPScScPa_Passalinae	0	640	38.0061	0.1700	0.1518	0.1098	6.4610
CPSHyHy_Hydrophilinae	0	1575	169.7393	0.0434	0.0393	0.0298	7.3620
CPSHy_Hydrochidae	0	164	52.1099	0.0979	0.0847	0.0547	5.1000
CPSHy_Spercheidae	0	19	52.1099	0.0565	0.0442	0.0198	2.9440
CPSHy_Epimetopidae	0	27	62.9916	0.0523	0.0419	0.0203	3.2960
CPSHy_Georissidae	0	77	132.4708	0.0328	0.0277	0.0162	4.3440
CPSHyHy_Sphaeridiinae	0	759	123.3365	0.0538	0.0482	0.0352	6.6320

CPSHy_Helophoridae	0	183	123.3365	0.0422	0.0367	0.0240	5.2090
CPSStSt_Staphylininae	0	6642	135.391	0.0650	0.0599	0.0480	8.8010
CPSStSt_Phloeocharinae	0	53	82.7428	0.0480	0.0398	0.0221	3.9700
CPSStSt_Pseudopsinae	0	49	82.7428	0.0470	0.0389	0.0212	3.8920
CPSStSt_Osoriinae	0	2058	101.7458	0.0750	0.0682	0.0524	7.6290
CPSStSt_Oxytelinae	0	2000	101.7458	0.0747	0.0679	0.0521	7.6010
CPSStSt_Paederinae	0	5962	145.6276	0.0597	0.0549	0.0439	8.6930
CPSStSt_Piestinae	0	107	131.9698	0.0354	0.0302	0.0186	4.6730
CPSStSt_Euaesthetinae	0	724	121.6028	0.0541	0.0485	0.0353	6.5850
CPSStSt_Oxyporinae	0	100	67.0615	0.0687	0.0585	0.0356	4.6050
CPSStSt_Steninae	0	2109	67.0615	0.1141	0.1038	0.0799	7.6540
CPSStSt_Tachyporinae	0	1519	161.2964	0.0454	0.0411	0.0312	7.3260
CPSStSi_Silphinae	0	113	161.2964	0.0293	0.0251	0.0155	4.7270
CPSStSt_Habrocerinae	0	22	151.4948	0.0204	0.0161	0.0075	3.0910
CPSStSt_Micropeplinae	0	80	151.4948	0.0289	0.0244	0.0144	4.3820
CPSStSt_Megalopsidiinae	0	164	121.403	0.0420	0.0363	0.0235	5.1000
CPSStSt_Aleocharinae	0	12182	99.3789	0.0947	0.0877	0.0715	9.4080
CPSStSt_Proteininae	0	189	99.3789	0.0527	0.0458	0.0300	5.2420
CPSStSt_Omalinae	0	1431	134.6895	0.0539	0.0488	0.0369	7.2660
CPSStSt_Glypholomatinae	0	8	134.6895	0.0154	0.0112	0.0039	2.0790
CPSStSt_Scaphidiinae	0	1276	214.7902	0.0333	0.0301	0.0226	7.1510
CPSStLe_Leiodinae	0	1423	102.8642	0.0706	0.0639	0.0483	7.2610
CPSStLeCh_Nargus	0	3,213	128.9718	0.0626	0.0572	0.0448	8.0750
CPSStLe_Camiarinae	0	77	128.9718	0.0337	0.0284	0.0167	4.3440
CPSSt_Agyrtidae	0	61	138.9099	0.0296	0.0247	0.0140	4.1110
CPSStPt_Ptilinae	0	410	157.3755	0.0382	0.0338	0.0237	6.0160
CPSStPt_Acrotrichinae	0	190	132.8046	0.0395	0.0343	0.0225	5.2470
CPSStPt_Cephaloplectinae	0	28	132.8046	0.0251	0.0201	0.0099	3.3320

CPSStHyHy_Hydraena	0	1094	225.7276	0.0310	0.0279	0.0208	6.9980
CPSHi_Sphaeritidae	0	4	172.0585	0.0081	0.0053	0.0015	1.3860
CPSHiHi_Saprininae	0	600	106.8738	0.0599	0.0534	0.0384	6.3970
CPSHiHi_Onthophilinae	0	76	76.2296	0.0568	0.0479	0.0281	4.3310
CPSHiHi_Abraeinae	0	422	76.2296	0.0793	0.0702	0.0494	6.0450
CPSHiHi_Dendrophilinae	0	417	118.8858	0.0507	0.0449	0.0316	6.0330
CPSHiHi_Hetaeriinae	0	313	51.9671	0.1106	0.0973	0.0668	5.7460
CPSHiHi_Tribalinae	0	200	51.9671	0.1020	0.0887	0.0585	5.2980
CPSHiHi_Histerinae	0	1800	85.4545	0.0877	0.0796	0.0608	7.4960
CPSHi_Synteliidae	0	9	184.8453	0.0119	0.0087	0.0032	2.1970
CPSStSc_Scydmaeninae	0	4500	162.8551	0.0517	0.0474	0.0375	8.4120
CPSStSc_Mastiginae	0	86	162.8551	0.0274	0.0232	0.0138	4.4540
CPEScCl_Clambinae	0	150	191.4556	0.0262	0.0226	0.0144	5.0110
CPESc_Eucinetidae	0	37	191.4556	0.0189	0.0154	0.0080	3.6110
CPBDeDe_Laricobiinae	0	17	253.3758	0.0112	0.0087	0.0038	2.8330
CPEScSc_Scirtidae	0	1000	130.5056	0.0529	0.0476	0.0354	6.9080
CAC_Elaphrinae	0	49	124.3008	0.0313	0.0259	0.0141	3.8920
CAC_Brachininae	0	655	86.9153	0.0746	0.0667	0.0483	6.4850
CAC_Siagoninae	0	84	86.9153	0.0510	0.0431	0.0257	4.4310
CA_Trachypachidae	0	4	109.0848	0.0127	0.0084	0.0024	1.3860
CAC_Rhysodinae	0	170	133.0375	0.0386	0.0334	0.0217	5.1360
CAC_Loricerinae	0	10	133.0375	0.0173	0.0128	0.0048	2.3030
CAC_Migadopinae	0	30	149.1196	0.0228	0.0184	0.0091	3.4010
CAC_Cicindelinae	0	2100	154.4502	0.0495	0.0450	0.0346	7.6500
CAC_Paussinae	0	724	153.3424	0.0429	0.0384	0.0280	6.5850
CAC_Harpalinae	50	20000	153.3424	0.0646	0.0601	0.0496	9.9030
CAC_Carabinae	0	665	167.4721	0.0388	0.0347	0.0251	6.5000
CAC_Trechinae	0	5317	167.4721	0.0512	0.0471	0.0375	8.5790

CAC_Gehringiinae	0	1	181.1929	0.0000	0.0000	0.0000	0.0000
CAC_Scaritinae	0	1850	157.3076	0.0478	0.0434	0.0332	7.5230
CAC_Omophroninae	0	60	157.3076	0.0260	0.0217	0.0123	4.0940
CAG_Sphanglerogyrinae	0	1	152.8987	0.0000	0.0000	0.0000	0.0000
CAG_Gyrininae	0	1100	152.8987	0.0458	0.0413	0.0308	7.0030
CA_Haliplidae	50	204	189.4683	0.0281	0.0244	0.0161	5.3180
CAN_Notomicrinae	50	9	148.5418	0.0148	0.0108	0.0040	2.1970
CAN_Noterinae	50	235	148.5418	0.0368	0.0321	0.0215	5.4600
CAD_Dytiscinae	0	373	115.6895	0.0512	0.0452	0.0315	5.9220
CAD_Laccophilinae	0	394	115.6895	0.0517	0.0457	0.0320	5.9760
CAD_Copelatinae	0	540	140.5173	0.0448	0.0399	0.0285	6.2920
CA_Amphizoidae	0	5	88.7516	0.0181	0.0124	0.0038	1.6090
CAD_Hydroporinae	0	1956	88.7516	0.0854	0.0776	0.0595	7.5790
CAD_Agabinae	0	363	111.4222	0.0529	0.0467	0.0325	5.8940
CAD_Colymbetinae	0	129	129.4615	0.0375	0.0322	0.0203	4.8600
CAD_Coptotominae	0	5	110.5393	0.0146	0.0099	0.0030	1.6090
CAD_Lancetinae	0	22	110.5393	0.0280	0.0221	0.0102	3.0910
CA_Paelobiidae	0	6	134.079	0.0134	0.0093	0.0030	1.7920
CA_Aspidytidae	0	2	134.079	0.0052	0.0030	0.0007	0.6930
CM_Torridincolidae	0	60	178.393	0.0230	0.0192	0.0108	4.0940
CM_Hydroscaphidae	0	13	178.393	0.0144	0.0109	0.0044	2.5650
CAr_Cupedidae	0	30	173.4713	0.0196	0.0158	0.0078	3.4010
CM_Sphaeriusidae	0	23	173.4713	0.0181	0.0143	0.0067	3.1350

Supplementary Table 12. Full results of PGLS analyses of herbivory and diversification in Coleoptera. Using three values for relative extinction rates (e=0, 0.5, and 0.9). Herbivory is treated either as a percentage (%) or as presence or absence (PA).

Variables	r^2	*P*
Diversification rate (e=0) ~ herbivory (%)	0.0019	0.5389
Diversification rate (e=0.5) ~ herbivory (%)	0.0017	0.5882
Diversification rate (e=0.9) ~ herbivory (%)	0.0007	0.8102
Ln-species ~ herbivory (%)	0.0022	0.4875
Diversification rate (e=0) ~ herbivory (PA)	0.0002	0.9271
Diversification rate (e=0.5) ~ herbivory (PA)	0.0005	0.8440
Diversification rate (e=0.9) ~ herbivory (PA)	0.0024	0.4683
Ln-species ~ herbivory (PA)	0.0207	0.0013
Ln-species ~ diversification rate (e=0)	0.3116	<0.0001
Ln-species ~ diversification rate (e=0.5)	0.3627	<0.0001
Ln-species ~ diversification rate (e=0.9)	0.4845	<0.0001
Ln-species ~ age	0.0001	0.9679

Supplementary Table 13. Data for analyses of herbivory and diversification in Diptera. Including data on species richness, herbivory (0 = absent, 1 = present), stem-group ages (Ma), estimated diversification rates (in species per million years; using three values for relative extinction rates, e=0, 0.5, and 0.9), and ln-transformed species richness for 142 families of Diptera, based on the data of Wiegmann and colleagues[6].

Clade	Species	Herb.	Stem age	Div. rate e=0	Div.rate e=0.5	Div. rate e=0.9	Ln species
Deuterophlebiidae	14	0	240.000	0.010996	0.008395	0.003470	2.639057
Tipulidae	15204	1	230.604	0.041757	0.038751	0.031774	9.629314
Trichoceridae	160	0	230.604	0.022008	0.019029	0.012260	5.075174
Perissomatidae	5	0	214.402	0.007507	0.005124	0.001569	1.609438
Canthyloscelididae	16	0	137.851	0.020113	0.015525	0.006647	2.772589
Scatopsidae	308	0	137.851	0.041567	0.036563	0.025073	5.730100
Axymyiidae	6	0	198.257	0.009038	0.006319	0.002045	1.791759
Manotidae	155	0	132.697	0.038007	0.032832	0.021080	5.043425
Ditomyiidae	93	0	132.697	0.034157	0.029015	0.017501	4.532599
Lygistorrhinidae	30	0	96.233	0.035343	0.028481	0.014143	3.401197
Cecidomyiidae	6059	1	96.233	0.090502	0.083301	0.066591	8.709300
Sciaridae	2223	1	117.367	0.065662	0.059760	0.046078	7.706613
Mycetophilidae	3961	0	124.635	0.066468	0.060909	0.048012	8.284252
Bolitophilidae	59	0	105.929	0.038493	0.032108	0.018096	4.077537
Keroplatidae	909	0	105.929	0.064311	0.057777	0.042667	6.812345

Diadocidiidae	19	0	143.818	0.020473	0.016010	0.007159	2.944439
Bibionidae	751	1	147.394	0.044923	0.040230	0.029382	6.621406
Pachyneuridae	5	0	147.394	0.010919	0.007454	0.002283	1.609438
Anisopodidae	159	0	214.402	0.023642	0.020438	0.013159	5.068904
Apystomyiidae	1	0	167.217	0.000000	0.000000	0.000000	0.000000
Pipunculidae	1831	0	89.978	0.083494	0.075796	0.057958	7.512618
Chyromyidae	107	0	59.279	0.078828	0.067292	0.041347	4.672829
Opomyzidae	61	1	54.224	0.075812	0.063329	0.035886	4.110874
Sepsidae	375	0	46.561	0.127294	0.112464	0.078350	5.926926
Acartophthalmidae	4	0	46.561	0.029774	0.019679	0.005635	1.386294
Richardiidae	174	1	53.502	0.096427	0.083579	0.054333	5.159055
Lonchaeidae	480	1	53.502	0.115394	0.102477	0.072703	6.173786
Ulidiidae	671	1	48.160	0.135150	0.120788	0.087615	6.508769
Ctenostylidae	10	0	33.308	0.069130	0.051181	0.019270	2.302585
Tachiniscidae	3	0	24.900	0.044121	0.027837	0.007322	1.098612
Pyrgotidae	351	0	24.900	0.235375	0.207652	0.143918	5.860786
Tephritidae	4632	1	29.931	0.282007	0.258856	0.205142	8.440744
Platystomatidae	1161	1	38.320	0.184161	0.166095	0.124274	7.057037
Piophilidae	82	0	55.742	0.079055	0.066838	0.039616	4.406719
Milichiidae	276	0	43.074	0.130484	0.114476	0.077772	5.620401
Chloropidae	2863	1	43.074	0.184792	0.168708	0.131407	7.959625
Somatiidae	7	0	57.408	0.033896	0.024148	0.008187	1.945910
Diopsidae	183	1	58.264	0.089412	0.077609	0.050716	5.209486

Marginidae	3	0	54.324	0.020223	0.012760	0.003356	1.098612
Nannodastiidae	5	0	50.968	0.031577	0.021555	0.006602	1.609438
Canacidae	312	0	50.968	0.112679	0.099142	0.068059	5.743003
Inbiomyiidae	10	0	44.957	0.051218	0.037920	0.014277	2.302585
Neminidae	14	0	44.957	0.058702	0.044818	0.018527	2.639057
Aulacigastridae	70	0	57.334	0.074101	0.062259	0.036050	4.248495
Neriidae	111	1	38.002	0.123930	0.105926	0.065390	4.709530
Pseudopomyzidae	34	0	38.002	0.092795	0.075318	0.038383	3.526361
Micropezidae	578	1	52.501	0.121133	0.107964	0.077569	6.359574
Tanypezidae	21	0	48.780	0.062413	0.049157	0.022522	3.044522
Strongylophthalmyiidae	47	0	48.780	0.078929	0.065151	0.035317	3.850148
Teratomyzidae	8	0	54.722	0.038000	0.027486	0.009697	2.079442
Heleomyzidae	811	0	54.722	0.122405	0.109761	0.080529	6.698268
Natalimyzidae	1	0	36.814	0.000000	0.000000	0.000000	0.000000
Heterocheilidae	2	0	36.814	0.018828	0.011014	0.002589	0.693147
Dryomyzidae	25	0	37.452	0.085946	0.068486	0.032676	3.218876
Phaeomyiidae	3	0	32.037	0.034292	0.021636	0.005691	1.098612
Conopidae	779	0	30.007	0.221882	0.198825	0.145530	6.658011
Sciomyzidae	600	0	30.007	0.213181	0.190137	0.136942	6.396930
Coelopidae	35	0	33.506	0.106112	0.086265	0.044220	3.555348
Helosciomyzidae	23	0	33.506	0.093581	0.074164	0.034715	3.135494
Huttoninidae	8	0	33.612	0.061867	0.044749	0.015787	2.079442

Helcomyzidae	12	0	33.612	0.073930	0.055689	0.022074	2.484907
Celyphidae	116	1	28.174	0.168721	0.144424	0.089647	4.753590
Lauxaniidae	1894	1	28.174	0.267850	0.243266	0.186291	7.546446
Chamaemyiidae	349	0	44.275	0.132244	0.116653	0.080812	5.855072
Rhopalomeridae	33	0	53.771	0.065026	0.052690	0.026689	3.496508
Fergusoninidae	29	1	43.657	0.077131	0.062030	0.030579	3.367296
Asteiidae	132	1	43.657	0.111845	0.096140	0.060613	4.882802
Xenasteiidae	13	0	34.564	0.074210	0.056299	0.022812	2.564949
Australimyzidae	9	0	34.564	0.063570	0.046565	0.017006	2.197225
Clusiidae	350	0	51.489	0.113771	0.100364	0.069544	5.857933
Neurochaetidae	20	0	45.522	0.065808	0.051654	0.023389	2.995732
Pallopteridae	66	1	45.522	0.092036	0.077140	0.044262	4.189655
Psilidae	320	1	49.845	0.115726	0.101882	0.070087	5.768321
Syringogastridae	10	0	49.845	0.046195	0.034201	0.012877	2.302585
Megamerinidae	15	0	72.230	0.037492	0.028789	0.012121	2.708050
Odiniidae	62	0	69.852	0.059084	0.049390	0.028061	4.127134
Agromyzidae	2800	1	64.968	0.122173	0.111510	0.086781	7.937375
Sphaeroceridae	1555	0	64.968	0.113120	0.102461	0.077767	7.349231
Periscelididae	32	0	61.197	0.056633	0.045809	0.023057	3.465736
Carnidae	90	0	61.197	0.073530	0.062384	0.037462	4.499810
Ephydridae	1980	1	63.860	0.118867	0.108021	0.082881	7.590852
Camillidae	40	0	51.677	0.071384	0.058448	0.030753	3.688879
Diastatidae	48	0	46.958	0.082440	0.068118	0.037064	3.871201

Curtonotidae	60	0	46.958	0.087192	0.072783	0.041133	4.094345
Drosophilidae	3944	1	51.112	0.161995	0.148438	0.116990	8.279951
Braulidae	7	0	44.285	0.043940	0.031304	0.010613	1.945910
Chryptochaetidae	33	0	44.285	0.078954	0.063976	0.032405	3.496508
Hippoboscidae	785	0	44.423	0.150052	0.134477	0.098475	6.665684
Glossinidae	23	0	44.423	0.070583	0.055938	0.026184	3.135494
Fanniidae	320	0	55.287	0.104334	0.091853	0.063188	5.768321
Muscidae	5155	1	50.663	0.168716	0.155038	0.123302	8.547722
Anthomyiidae	1895	1	34.847	0.216577	0.196701	0.150635	7.546974
Scathophagidae	394	1	34.847	0.171505	0.151686	0.106075	5.976351
Rhinophoridae	167	0	32.853	0.155786	0.134869	0.087295	5.117994
Calliphoridae	1889	0	28.745	0.262442	0.238347	0.182503	7.543803
Tachinidae	9622	0	28.745	0.319079	0.294969	0.239007	9.171807
Oestridae	192	0	31.171	0.168667	0.146596	0.096267	5.257495
Sarcophagidae	3073	0	31.171	0.257625	0.235399	0.183849	8.030410
Syrphidae	5905	1	102.291	0.084890	0.078116	0.062395	8.683555
Lonchopteridae	58	0	131.476	0.030884	0.025742	0.014467	4.060443
Ironomyiidae	3	0	94.361	0.011643	0.007346	0.001932	1.098612
Phoridae	4042	1	94.361	0.088007	0.080664	0.063629	8.304495
Platypezidae	252	0	110.808	0.049901	0.043682	0.029438	5.529429
Opetiidae	5	0	124.331	0.012945	0.008836	0.002706	1.609438
Atelestidae	10	0	157.090	0.014658	0.010852	0.004086	2.302585
Dolichopodidae	7151	1	124.768	0.071132	0.065578	0.052687	8.875007

Hybotidae	1838	0	115.922	0.064840	0.058865	0.045019	7.516433
Empididae	2982	0	115.922	0.069015	0.063038	0.049177	8.000349
Asilidae	7429	0	133.275	0.066878	0.061678	0.049610	8.913147
Mydidae	461	0	118.779	0.051637	0.045820	0.032414	6.133398
Apioceridae	169	0	118.779	0.043189	0.037403	0.024240	5.129899
Scenopinidae	414	0	117.426	0.051316	0.045434	0.031891	6.025866
Therevidae	1123	0	117.426	0.059814	0.053919	0.040273	7.023759
Evocoidae	1	0	122.902	0.000000	0.000000	0.000000	0.000000
Apsilocephalidae	3	0	122.902	0.008939	0.005640	0.001483	1.098612
Bombyliidae	5288	0	141.769	0.060473	0.055585	0.044243	8.573195
Pantophthalmidae	20	1	155.001	0.019327	0.015170	0.006869	2.995732
Xylomyidae	134	0	138.214	0.035437	0.030475	0.019247	4.897840
Stratiomyidae	2660	1	138.214	0.057057	0.052045	0.040422	7.886081
Hilarimorphidae	33	0	161.597	0.021637	0.017533	0.008881	3.496508
Acroceridae	392	0	161.597	0.036952	0.032678	0.022843	5.971262
Nemestrinidae	275	0	164.466	0.034152	0.029959	0.020347	5.616771
Xylophagidae	136	0	164.466	0.029870	0.025700	0.016260	4.912655
Rhagionidae	656	0	153.004	0.042392	0.037872	0.027432	6.486161
Vermileonidae	59	0	153.004	0.026650	0.022229	0.012529	4.077537
Pelecorhynchidae	50	0	154.753	0.025279	0.020928	0.011470	3.912023
Oreoleptidae	1	0	144.123	0.000000	0.000000	0.000000	0.000000
Tabanidae	4387	0	130.002	0.064510	0.059180	0.046814	8.386401
Athericidae	134	0	130.002	0.037675	0.032401	0.020463	4.897840

Dixidae	185	0	213.854	0.024411	0.021195	0.013866	5.220356
Corethrellidae	66	0	143.571	0.029182	0.024459	0.014034	4.189655
Chaoboridae	55	0	143.571	0.027912	0.023209	0.012930	4.007333
Culicidae	3610	0	181.736	0.045073	0.041261	0.032417	8.191463
Ceratopogonidae	5622	1	210.001	0.041116	0.037816	0.030159	8.634443
Chironomidae	6951	1	210.001	0.042127	0.038827	0.031168	8.846641
Thaumaleidae	173	0	163.056	0.031604	0.027389	0.017794	5.153292
Simuliidae	2079	0	163.056	0.046853	0.042605	0.032758	7.639642
Blephariceridae	318	0	221.363	0.026030	0.022913	0.015754	5.762051
Tanyderidae	38	0	180.001	0.020209	0.016502	0.008598	3.637586
Psychodidae	2886	0	180.001	0.044264	0.040415	0.031490	7.967627
Ptychopteridae	74	0	231.097	0.018624	0.015683	0.009157	4.304065
Nymphomyiidae	7	0	236.468	0.008229	0.005863	0.001988	1.945910

Supplementary Table 14. Full results of PGLS analyses of herbivory and diversification in Diptera. Using three values for relative extinction rates (e=0, 0.5, and 0.9).

Variables	r^2	*P*
Diversification rate (e=0) ~ herbivory	0.1234	<0.0001
Diversification rate (e=0.5) ~ herbivory	0.1307	<0.0001
Diversification rate (e=0.9) ~ herbivory	0.1438	<0.0001
Ln-species ~ herbivory	0.2374	<0.0001
Ln-species ~ diversification rate (e=0)	0.6845	<0.0001
Ln-species ~ diversification rate (e=0.5)	0.7004	<0.0001
Ln-species ~ diversification rate (e=0.9)	0.7167	<0.0001
Ln-species ~ age	0.0018	0.7793

Supplementary Table 15. Data for analyses of herbivory and diversification in Hemiptera. Including species richness, herbivory (0 = absent, 1 = present), stem-group ages (Ma), estimated diversification rates (in species per million years, using three values for relative extinction rates, e=0, 0.5, and 0.9), and ln-transformed species richness for 93 families of Hemiptera (including clade abbreviations used in the phylogeny; Supplementary Data 6). Clade ages and species richness estimates are from Rainford and colleagues[4].

Clade abbrev.	Clade full name	Species	Herb.	Stem age	Ln species
He_Psylloi	Psylloidea	2500	1	321.3975	7.824046
He_Aleyrod	Aleyrodoidea	1560	1	321.3975	7.352441
He_Coccoid	Coccoidea	8000	1	286.4427	8.987197
He_Phyllox	Phylloxeroidea	75	1	87.1386	4.317488
He_Aphidoi	Aphidoidea	4300	1	87.1386	8.366370
He_Myerslo	Myerslopiidae	20	1	287.1723	2.995732
He_Cicadel	Cicadellidae	20000	1	170.6715	9.903488
He_Membrac	Membracidae	3450	1	127.2759	8.146130
He_Aetalio	Aetalionidae	42	1	127.2759	3.737670
He_Cicadid	Cicadidae	1300	1	224.8581	7.170120
He_Machaer	Machaerotidae	110	1	162.3959	4.700480
He_Cercopi	Cercopidae	1400	1	128.2813	7.244228
He_Aphroph	Aphrophoridae	820	1	101.9798	6.709304
He_Clastop	Clastopteridae	80	1	69.1448	4.382027

He_Epipygi	Epipygidae	27	1	69.1448	3.295837
He_Derbida	Derbidae	1700	1	304.7509	7.438384
He_Meenopl	Meenoplidae	158	1	182.2549	5.062595
He_Flatida	Flatidae	1446	1	182.2549	7.276556
He_Delphac	Delphacidae	2029	1	190.6426	7.615298
He_Cixiida	Cixiidae	2223	1	190.6426	7.706613
He_Achilid	Achilidae	503	1	176.3839	6.220590
He_Tropidu	Tropiduchidae	575	1	176.3839	6.354370
He_Fulgori	Fulgoridae	687	1	154.2369	6.532334
He_Tettigo	Tettigometridae	73	1	96.1775	4.290459
He_Dictyop	Dictyopharidae	731	1	96.1775	6.594413
He_Issidae	Issidae	924	1	88.1318	6.828712
He_Nogodin	Nogodinidae	286	1	88.1318	5.655992
He_Eurybra	Eurybrachyidae	189	1	157.4174	5.241747
He_Ricanni	Ricaniidae	417	1	141.7222	6.033086
He_Lophopi	Lophopidae	138	1	108.6603	4.927254
He_Achilix	Achilixiidae	24	1	81.1577	3.178054
He_Calisce	Caliscelidae	202	1	81.1577	5.308268
He_Pelorid	Peloridiidae	12	0	336.1534	2.484907
He_Naucori	Naucoridae	500	0	192.3887	6.214608
He_Notonec	Notonectidae	350	0	129.4200	5.857933
He_Apheloc	Aphelocheiridae	400	0	129.4200	5.991465
He_Ochteri	Ochteridae	50	0	179.2143	3.912023

He_Gelasto	Gelastocoridae	100	0	179.2143	4.605170
He_Belosto	Belostomatidae	150	0	239.4364	5.010635
He_Nepidae	Nepidae	225	0	239.4364	5.416100
He_Corixid	Corixidae	600	0	263.6770	6.396930
He_Enicoce	Enicocephalidae	400	0	250.2628	5.991465
He_Schizop	Schizopteridae	120	0	160.0610	4.787492
He_Dipsoco	Dipsocoridae	30	0	160.0610	3.401197
He_Hydrome	Hydrometridae	110	0	159.8218	4.700480
He_Veliida	Veliidae	720	0	118.0079	6.579251
He_Gerrida	Gerridae	620	0	118.0079	6.429719
He_Hermato	Hermatobatidae	8	0	122.3117	2.079442
He_Mesovel	Mesoveliidae	35	0	122.3117	3.555348
He_Macrove	Macroveliidae	3	0	135.2463	1.098612
He_Paraphy	Paraphrynoveliidae	2	0	101.3662	0.693147
He_Hebrida	Hebridae	150	0	101.3662	5.010635
He_Pleidae	Pleidae	40	0	253.9733	3.688879
He_Saldida	Saldidae	265	0	172.7331	5.579730
He_Leptopo	Leptopodidae	40	0	172.7331	3.688879
He_Miridae	Miridae	10000	1	140.7011	9.210340
He_Tingida	Tingidae	2000	1	140.7011	7.600902
He_Thaumas	Thaumastocoridae	19	1	204.6460	2.944439
He_Plokiop	Plokiophilidae	6	0	177.0310	1.791759
He_Lycetoc	Lyctocoridae	27	0	147.9229	3.295837

He_Cimicid	Cimicidae	100	0	125.2985	4.605170
He_Anthoco	Anthocoridae	600	0	125.2985	6.396930
He_Joppeic	Joppeicidae	1	0	231.8878	0.000000
He_Microph	Microphysidae	30	0	212.2648	3.401197
He_Phymati	Phymatidae	280	0	178.6019	5.634790
He_Nabidae	Nabidae	400	0	103.2647	5.991465
He_Velocip	Velocipedidae	31	0	103.2647	3.433987
He_Reduvii	Reduviidae	6420	0	95.2461	8.767173
He_Berytid	Berytidae	100	1	95.2461	4.605170
He_Aradida	Aradidae	2000	1	272.6348	7.600902
He_Canopid	Canopidae	8	0	131.8250	2.079442
He_Cydnida	Cydnidae	617	1	131.8250	6.424869
He_Dinidor	Dinidoridae	90	1	123.5365	4.499810
He_Tessara	Tessaratomidae	250	1	123.5365	5.521461
He_Platasp	Plataspidae	500	1	124.1235	6.214608
He_Pentato	Pentatomidae	4500	1	124.1235	8.411833
He_Phloeid	Phloeidae	3	1	141.8908	1.098612
He_Lestoni	Lestoniidae	2	1	118.2776	0.693147
He_Acantho	Acanthosomatidae	200	1	72.5306	5.298317
He_Scutell	Scutelleridae	500	1	72.5306	6.214608
He_Idiosto	Idiostolidae	4	1	178.6456	1.386294
He_Hyoceph	Hyocephalidae	3	1	178.6456	1.098612
He_Alydida	Alydidae	250	1	222.7754	5.521461

He_Coreida	Coreidae	1900	1	213.9783	7.549609
He_Rhopali	Rhopalidae	200	1	213.9783	5.298317
He_Stenoce	Stenocephalidae	30	1	150.5309	3.401197
He_Largida	Largidae	120	1	104.7187	4.787492
He_Pyrrhoc	Pyrrhocoridae	225	1	104.7187	5.416100
He_Piesmat	Piesmatidae	40	1	185.3944	3.688879
He_Lygaeio	Lygaeidae	4400	1	157.5471	8.389360
He_Malcida	Malcidae	20	1	134.1621	2.995732
He_Termita	Termitaphididae	9	0	94.7508	2.197225
He_Colobat	Colobathristidae	90	1	94.7508	4.499810

Clade abbrev.	Clade full name	Div. rate e=0	Div.rate e=0.5	Div. rate e=0.9
He_Psylloi	Psylloidea	0.02434383	0.02218841	0.01719072
He_Aleyrod	Aleyrodoidea	0.02287647	0.02072180	0.01573008
He_Coccoid	Coccoidea	0.03137520	0.02895579	0.02334057
He_Phyllox	Phylloxeroidea	0.04954737	0.04174483	0.02442352
He_Aphidoi	Aphidoidea	0.09601222	0.08806035	0.06961181
He_Myerslo	Myerslopiidae	0.01043183	0.00818803	0.00370757
He_Cicadel	Cicadellidae	0.05802660	0.05396560	0.04453791
He_Membrac	Membracidae	0.06400371	0.05855996	0.04593289
He_Aetalio	Aetalionidae	0.02936667	0.02410553	0.01280086
He_Cicadid	Cicadidae	0.03188731	0.02880813	0.02167782

He_Machaer	Machaerotidae	0.02894458	0.02473205	0.01525001
He_Cercopi	Cercopidae	0.05647142	0.05107365	0.03857188
He_Aphroph	Aphrophoridae	0.06579052	0.05900557	0.04331873
He_Clastop	Clastopteridae	0.06337464	0.05352972	0.03161556
He_Epipygi	Epipygidae	0.04766572	0.03816711	0.01852538
He_Derbida	Derbidae	0.02440808	0.02213554	0.01686977
He_Meenopl	Meenoplidae	0.02777755	0.02400900	0.01544764
He_Flatida	Flatidae	0.03992516	0.03612578	0.02732533
He_Delphac	Delphacidae	0.03994542	0.03631216	0.02789061
He_Cixiida	Cixiidae	0.04042440	0.03679091	0.02836757
He_Achilid	Achilidae	0.03526734	0.03134883	0.02231349
He_Tropidu	Tropiduchidae	0.03602579	0.03210588	0.02305945
He_Fulgori	Fulgoridae	0.04235260	0.03786799	0.02750810
He_Tettigo	Tettigometridae	0.04460980	0.03754431	0.02187761
He_Dictyop	Dictyopharidae	0.06856503	0.06137229	0.04475127
He_Issidae	Issidae	0.07748295	0.06963033	0.05146633
He_Nogodin	Nogodinidae	0.06417652	0.05635123	0.03840147
He_Eurybra	Eurybrachyidae	0.03329840	0.02892868	0.01896666
He_Ricanni	Ricaniidae	0.04256980	0.03769582	0.02647330
He_Lophopi	Lophopidae	0.04534548	0.03903290	0.02473624
He_Achilix	Achilixiidae	0.03915899	0.03112124	0.01471114
He_Calisce	Caliscelidae	0.06540683	0.05692693	0.03757220
He_Pelorid	Peloridiidae	0.00739218	0.00556830	0.00220714

He_Naucori	Naucoridae	0.03230236	0.02870989	0.02042668
He_Notonec	Notonectidae	0.04526297	0.03992922	0.02766757
He_Apheloc	Aphelocheiridae	0.04629473	0.04095823	0.02867509
He_Ochteri	Ochteridae	0.02182874	0.01807154	0.00990408
He_Gelasto	Gelastocoridae	0.02569644	0.02188427	0.01332909
He_Belosto	Belostomatidae	0.02092679	0.01805963	0.01155346
He_Nepidae	Nepidae	0.02262020	0.01974381	0.01316732
He_Corixid	Corixidae	0.02426048	0.02163802	0.01558434
He_Enicoce	Enicocephalidae	0.02394069	0.02118099	0.01482893
He_Schizop	Schizopteridae	0.02991042	0.02563175	0.01597658
He_Dipsoco	Dipsocoridae	0.02124938	0.01712372	0.00850286
He_Hydrome	Hydrometridae	0.02941076	0.02513038	0.01549562
He_Veliida	Veliidae	0.05575263	0.04989066	0.03634578
He_Gerrida	Gerridae	0.05448550	0.04862542	0.03509550
He_Hermato	Hermatobatidae	0.01700117	0.01229709	0.00433833
He_Mesovel	Mesoveliidae	0.02906793	0.02363120	0.01211335
He_Macrove	Macroveliidae	0.00812305	0.00512507	0.00134807
He_Paraphy	Paraphrynoveliidae	0.00683805	0.00400000	0.00094026
He_Hebrida	Hebridae	0.04943103	0.04265853	0.02729035
He_Pleidae	Pleidae	0.01452467	0.01189269	0.00625749
He_Saldida	Saldidae	0.03230261	0.02831159	0.01916566
He_Leptopo	Leptopodidae	0.02135595	0.01748608	0.00920053
He_Miridae	Miridae	0.06546033	0.06053466	0.04910164

He_Tingida	Tingidae	0.05402163	0.04909880	0.03768846
He_Thaumas	Thaumastocoridae	0.01438796	0.01125155	0.00503122
He_Plokiop	Plokiophilidae	0.01012116	0.00707652	0.00229036
He_Lycetoc	Lyctocoridae	0.02228078	0.01784076	0.00865947
He_Cimicid	Cimicidae	0.03675359	0.03130104	0.01906458
He_Anthoco	Anthocoridae	0.05105352	0.04553484	0.03279555
He_Joppeic	Joppeicidae	0.00000000	0.00000000	0.00000000
He_Microph	Microphysidae	0.01602337	0.01291236	0.00641169
He_Phymati	Phymatidae	0.03154944	0.02768844	0.01883430
He_Nabidae	Nabidae	0.05802045	0.05133230	0.03593803
He_Velocip	Velocipedidae	0.03325422	0.02684934	0.01342467
He_Reduvii	Reduviidae	0.09204758	0.08477179	0.06788718
He_Berytid	Berytidae	0.04835022	0.04117726	0.02507990
He_Aradida	Aradidae	0.02787943	0.02533886	0.01945022
He_Canopid	Canopidae	0.01577426	0.01140965	0.00402525
He_Cydnida	Cydnidae	0.04873786	0.04349206	0.03138073
He_Dinidor	Dinidoridae	0.03642494	0.03090352	0.01855755
He_Tessara	Tessaratomidae	0.04469498	0.03911642	0.02634236
He_Platasp	Plataspidae	0.05006794	0.04449970	0.03166091
He_Pentato	Pentatomidae	0.06776986	0.06218732	0.04923520
He_Phloeid	Phloeidae	0.00774266	0.00488507	0.00128494
He_Lestoni	Lestoniidae	0.00586034	0.00342808	0.00080582
He_Acantho	Acanthosomatidae	0.07304941	0.06356156	0.04190989

He_Scutell	Scutelleridae	0.08568257	0.07615350	0.05418214
He_Idiosto	Idiostolidae	0.00776003	0.00512910	0.00146863
He_Hyoceph	Hyocephalidae	0.00614967	0.00388001	0.00102058
He_Alydida	Alydidae	0.02478488	0.02169138	0.01460773
He_Coreida	Coreidae	0.03528213	0.03204525	0.02454338
He_Rhopali	Rhopalidae	0.02476100	0.02154498	0.01420588
He_Stenoce	Stenocephalidae	0.02259468	0.01820782	0.00904118
He_Largida	Largidae	0.04571764	0.03917775	0.02441997
He_Pyrrhoc	Pyrrhocoridae	0.05172047	0.04514368	0.03010671
He_Piesmat	Piesmatidae	0.01989747	0.01629189	0.00857219
He_Lygaeio	Lygaeidae	0.05324985	0.04885168	0.03864761
He_Malcida	Malcidae	0.02232920	0.01752637	0.00793600
He_Termita	Termitaphididae	0.02318951	0.01698601	0.00620350
He_Colobat	Colobathristidae	0.04749099	0.04029214	0.02419541

Supplementary Table 16. Full results of PGLS analyses of herbivory and diversification in Hemiptera. Using three values for relative extinction rates (e=0, 0.5, and 0.9).

Variables	r^2	*P*
Diversification rate (e=0) ~ herbivory	0.0907	0.0002
Diversification rate (e=0.5) ~ herbivory	0.0922	0.0002
Diversification rate (e=0.9) ~ herbivory	0.0926	0.0002
Ln-species ~ herbivory	0.0925	0.0002
Ln-species ~ diversification rate (e=0)	0.6257	<0.0001
Ln-species ~ diversification rate (e=0.5)	0.6685	<0.0001
Ln-species ~ diversification rate (e=0.9)	0.7452	<0.0001
Ln-species ~ age	0.0074	0.5113

Supplementary Table 17. Data for analyses of herbivory and diversification in Hymenoptera. Including species richness, herbivory (0 = absent, 1 = present), stem-group ages (Ma), estimated diversification rates (in species per million years), ln-transformed species richness, and estimated diversification rates (with relative extinction fractions of e=0, 0.5, 0.9) for 77 families of Hymenoptera (including clade abbreviations used in the phylogeny; Supplementary Data 7). Clade ages and species richness estimates are from Rainford and colleagues[4].

Clade abbrev.	Clade full name	Species	Herb.	Stem age	Ln species
Hy_Xyelida	Xyelidae	50	1	257.2901	3.912023
Hy_Blastic	Blasticotomidae	10	1	187.4192	2.302585
Hy_Pergida	Pergidae	500	1	148.593	6.214608
Hy_Tenthre	Tenthredinidae	4000	1	76.6877	8.294050
Hy_Argidae	Argidae	800	1	76.6877	6.684612
Hy_Cimbici	Cimbicidae	130	1	70.7022	4.867534
Hy_Diprion	Diprionidae	90	1	70.7022	4.499810
Hy_Pamphil	Pamphiliidae	250	1	186.4881	5.521461
Hy_Megalod	Megalodontesidae	40	1	186.4881	3.688879
Hy_Xiphydr	Xiphydriidae	100	0	239.9397	4.605170
Hy_Cephida	Cephidae	80	1	235.9364	4.382027
Hy_Siricid	Siricidae	95	0	136.7405	4.553877
Hy_Anaxeli	Anaxyelidae	1	0	136.7405	0.000000
Hy_Orussid	Orussidae	75	0	147.1097	4.317488

Hy_Stephan	Stephanidae	200	0	147.1097	5.298317
Hy_Megalyr	Megalyridae	50	0	177.379	3.912023
Hy_Scelion	Scelionidae	3000	0	110.3335	8.006368
Hy_Platyga	Platygastridae	1100	0	110.3335	7.003065
Hy_Helorid	Heloridae	7	0	176.2315	1.945910
Hy_Maaming	Maamingidae	2	0	121.9639	0.693147
Hy_Mymaron	Mymarommatidae	9	0	121.9639	2.197225
Hy_Diaprii	Diapriidae	2300	0	103.8914	7.740664
Hy_Monomac	Monomachidae	20	0	103.8914	2.995732
Hy_Proctot	Proctotrupidae	310	0	107.669	5.736572
Hy_Vanhorn	Vanhorniidae	5	0	107.669	1.609438
Hy_Pelecin	Pelecinidae	3	0	158.8355	1.098612
Hy_Roprion	Roproniidae	18	0	158.8355	2.890372
Hy_Figitid	Figitidae	1500	0	103.4603	7.313220
Hy_Cynipid	Cynipidae	1000	1	103.4603	6.907755
Hy_Liopter	Liopteridae	50	0	79.2072	3.912023
Hy_Ibaliid	Ibaliidae	50	0	79.2072	3.912023
Hy_Euchari	Eucharitidae	423	0	143.7984	6.047372
Hy_Encyrti	Encyrtidae	3735	0	123.1046	8.225503
Hy_Agaonid	Agaonidae	757	1	89.3513	6.629363
Hy_Mymarid	Mymaridae	1424	0	89.3513	7.261225
Hy_Eulophi	Eulophidae	4472	0	48.5766	8.405591
Hy_Pteroma	Pteromalidae	3506	0	48.5766	8.162231

Hy_Torymid	Torymidae	986	1	96.6394	6.893656
Hy_Perilam	Perilampidae	277	0	66.3105	5.624018
Hy_Aphelin	Aphelinidae	1168	0	66.3105	7.063048
Hy_Tetraca	Tetracampidae	50	0	83.4155	3.912023
Hy_Rotoiti	Rotoitidae	2	0	83.4155	0.693147
Hy_Trichog	Trichogrammatidae	839	0	97.5553	6.732211
Hy_Eupelmi	Eupelmidae	907	0	83.0424	6.810142
Hy_Eurytom	Eurytomidae	1424	1	45.7652	7.261225
Hy_Chalcid	Chalcididae	1464	0	45.7652	7.288928
Hy_Trigona	Trigonalidae	100	0	131.1363	4.605170
Hy_Braconi	Braconidae	20000	0	131.1363	9.903488
Hy_Ichneum	Ichneumonidae	22000	0	136.9222	9.998798
Hy_Ceraphr	Ceraphronidae	350	0	75.9177	5.857933
Hy_Megaspi	Megaspilidae	450	0	75.9177	6.109248
Hy_Gasteru	Gasteruptiidae	420	0	149.6838	6.040255
Hy_Evaniid	Evaniidae	500	0	110.2558	6.214608
Hy_Aulacid	Aulacidae	200	0	110.2558	5.298317
Hy_Sierolo	Sierolomorphidae	10	0	94.9912	2.302585
Hy_Mutilid	Mutillidae	5000	0	94.9912	8.517193
Hy_Sapygid	Sapygidae	80	0	149.283	4.382027
Hy_Scoliid	Scoliidae	300	0	122.8755	5.703782
Hy_Pompili	Pompilidae	4000	0	122.8755	8.294050
Hy_Ampulic	Ampulicidae	200	0	145.6401	5.298317

Hy_Bradyno	Bradynobaenidae	200	0	108.6693	5.298317
Hy_Formici	Formicidae	10000	0	108.6693	9.210340
Hy_Chrysid	Chrysididae	3000	0	163.1939	8.006368
Hy_Scoleby	Scolebythidae	3	0	140.9128	1.098612
Hy_Plumari	Plumariidae	20	0	123.6116	2.995732
Hy_Bethyli	Bethylidae	2000	0	123.6116	7.600902
Hy_Crabron	Crabronidae	8774	0	158.4218	9.079548
Hy_Tiphiid	Tiphiidae	1500	0	116.3308	7.313220
Hy_Vespida	Vespidae	4000	0	116.3308	8.294050
Hy_Sphecid	Sphecidae	724	0	154.1938	6.584791
Hy_Andreni	Andrenidae	2938	0	130.6676	7.985484
Hy_Halicti	Halictidae	4338	0	112.7858	8.375169
Hy_Stenotr	Stenotritidae	21	0	52.1914	3.044522
Hy_Colleti	Colletidae	2545	0	52.1914	7.841886
Hy_Melitti	Melittidae	191	0	69.5157	5.252273
Hy_Apidae	Apidae	5751	0	37.5614	8.657129
Hy_Megachi	Megachilidae	4120	0	37.5614	8.323608

Clade abbrev.	div. e=0	div. e=0.5	div. e=0.9
Hy_Xyelida	0.01520472	0.01258765	0.00689864
Hy_Blastic	0.01228575	0.00909591	0.0034247

Hy_Pergida	0.04182302	0.03717173	0.02644716
Hy_Tenthre	0.10815358	0.09911827	0.07815741
Hy_Argidae	0.08716667	0.07814439	0.05728707
Hy_Cimbici	0.06884559	0.05915021	0.03722499
Hy_Diprion	0.06364455	0.05399708	0.03242523
Hy_Pamphil	0.02960758	0.02591214	0.01745014
Hy_Megalod	0.01978078	0.01619634	0.00852191
Hy_Xiphydr	0.01919303	0.01634566	0.00995568
Hy_Cephida	0.01857291	0.01568771	0.00926543
Hy_Siricid	0.03330306	0.02831057	0.01712591
Hy_Anaxeli	0	0	0
Hy_Orussid	0.02934877	0.02472703	0.01446697
Hy_Stephan	0.0360161	0.03133823	0.02066315
Hy_Megalyr	0.0220546	0.01825852	0.01000655
Hy_Scelion	0.07256516	0.06628588	0.05172299
Hy_Platyga	0.0634718	0.05719774	0.04267633
Hy_Helorid	0.01104178	0.00786633	0.00266697
Hy_Maaming	0.00568322	0.00332447	0.00078146
Hy_Mymaron	0.01801537	0.01319602	0.00481935
Hy_Diaprii	0.07450727	0.06783961	0.05238147
Hy_Monomac	0.02883523	0.02263301	0.0102483
Hy_Proctot	0.0532797	0.04687185	0.03215973
Hy_Vanhorn	0.01494802	0.01020361	0.00312506

Hy_Pelecin	0.00691667	0.00436393	0.00114786
Hy_Roprion	0.01819727	0.01417373	0.00625334
Hy_Figitid	0.07068625	0.06399305	0.04848833
Hy_Cynipid	0.06676721	0.06007722	0.04459807
Hy_Liopter	0.04938974	0.04088869	0.02240898
Hy_Ibaliid	0.04938974	0.04088869	0.02240898
Hy_Euchari	0.04205452	0.03725067	0.02618833
Hy_Encyrti	0.06681719	0.06118881	0.04813244
Hy_Agaonid	0.07419437	0.06645159	0.04855662
Hy_Mymarid	0.08126603	0.07351633	0.05556651
Hy_Eulophi	0.17303786	0.15877331	0.12567813
Hy_Pteroma	0.16802804	0.15376476	0.1206797
Hy_Torymid	0.07133381	0.06417179	0.04760126
Hy_Perilam	0.08481338	0.07441467	0.05057128
Hy_Aphelin	0.10651478	0.09607463	0.07190624
Hy_Tetraca	0.04689803	0.03882586	0.02127845
Hy_Rotoiti	0.00830957	0.00486079	0.0011426
Hy_Trichog	0.06900917	0.06191621	0.04551568
Hy_Eupelmi	0.08200802	0.07367438	0.05439909
Hy_Eurytom	0.15866259	0.1435322	0.10848724
Hy_Chalcid	0.15926791	0.1441371	0.10908881
Hy_Trigona	0.03511743	0.02990761	0.01821588
Hy_Braconi	0.07552057	0.07023525	0.05796528

Hy_Ichneum	0.07302539	0.06796338	0.05621164
Hy_Ceraphr	0.07716163	0.06806896	0.04716604
Hy_Megaspi	0.08047198	0.07137097	0.05040281
Hy_Gasteru	0.04035343	0.03573857	0.02511208
Hy_Evaniid	0.05636536	0.05009677	0.03564314
Hy_Aulacid	0.04805477	0.04181329	0.02756997
Hy_Sierolo	0.02423998	0.01794638	0.00675698
Hy_Mutilid	0.08966297	0.08236811	0.06544192
Hy_Sapygid	0.02935382	0.02479386	0.01464367
Hy_Scoliid	0.0464192	0.04080523	0.02792059
Hy_Pompili	0.06749962	0.0618606	0.04877874
Hy_Ampulic	0.03637952	0.03165445	0.02087165
Hy_Bradyno	0.04875634	0.04242374	0.02797247
Hy_Formici	0.08475568	0.0783781	0.06357504
Hy_Chrysid	0.04906046	0.04481512	0.03496931
Hy_Scoleby	0.0077964	0.00491898	0.00129386
Hy_Plumari	0.02423504	0.01902229	0.00861336
Hy_Bethyli	0.0614902	0.05588679	0.04289895
Hy_Crabron	0.05731249	0.05293788	0.04278444
Hy_Tiphiid	0.06286573	0.05691304	0.04312372
Hy_Vespida	0.07129711	0.06534084	0.051523
Hy_Sphecid	0.04270464	0.0382183	0.02785171
Hy_Andreni	0.06111296	0.05581091	0.04351467

Hy_Halicti	0.0742573	0.06811365	0.05386011
Hy_Stenotr	0.0583338	0.04594426	0.02104968
Hy_Colleti	0.15025245	0.13697911	0.106202
Hy_Melitti	0.07555521	0.06565924	0.04309433
Hy_Apidae	0.2304794	0.21203032	0.16921914
Hy_Megachi	0.22160006	0.20315281	0.16035625

Supplementary Table 18. Full results of PGLS analyses of herbivory and diversification in Hymenoptera. Using three values for relative extinction rates (e=0, 0.5, and 0.9).

Variables	r^2	*P*
Diversification rate (e=0) ~ herbivory	0.0014	0.9027
Diversification rate (e=0.5) ~ herbivory	0.0013	0.9054
Diversification rate (e=0.9) ~ herbivory	0.0010	0.9301
Ln-species ~ herbivory	0.0016	0.8883
Ln-species ~ diversification rate (e=0)	0.4897	<0.0001
Ln-species ~ diversification rate (e=0.5)	0.5121	<0.0001
Ln-species ~ diversification rate (e=0.9)	0.5514	<0.0001
Ln-species ~ age	0.0839	0.0018

Supplementary Table 19. Data for analyses of herbivory and diversification in Orthoptera. Including data on species richness, herbivory (0 = absent, 1 = present), stem-group ages (Ma), estimated diversification rates (in species per million years), ln-transformed species richness, and estimated diversification rates (with relative extinction fractions of e=0, 0.5, 0.9) for 26 families of Orthoptera. Clade ages and species richness estimates are from Rainford and colleagues[4].

Clade abbrev.	Clade full name	Species	Herb.	Stem age	Ln species
Or_E_Grylli	Gryllidae	4664	1	195.1914	8.447629
Or_E_Myrme	Myrmecophilidae	8	0	129.7351	2.079442
Or_E_Gryllot	Gryllotalpidae	100	1	129.7351	4.605170
Or_E_Steno	Stenopelmatidae	28	0	129.6371	3.332205
Or_E_Gryllac	Gryllacrididae	675	0	88.9118	6.514713
Or_E_Anost	Anostostomatidae	206	1	88.9118	5.327876
Or_E_Rhaph	Rhaphidophoridae	497	0	118.8689	6.208590
Or_E_Tetti	Tettigoniidae	6827	1	77.8240	8.828641
Or_E_Proph	Prophalangopsidae	71	0	77.8240	4.262680
Or_C_Cylin	Cylindrachetidae	16	1	211.5551	2.772589
Or_C_Trida	Tridactylidae	132	0	186.0031	4.882802
Or_C_Theri	Thericleidae	220	1	117.4837	5.393628
Or_C_Tetri	Tetrigidae	1246	1	117.4837	7.127694
Or_C_Eusch	Euschmidtiidae	191	1	128.4138	5.252273
Or_C_Eumas	Eumastacidae	230	1	94.6346	5.438079

Or_C_Episa	Episactidae	64	1	63.3270	4.158883
Or_C_Choro	Chorotypidae	160	1	63.3270	5.075174
Or_C_Prosc	Proscopiidae	214	1	137.6494	5.365976
Or_C_Tanao	Tanaoceridae	3	1	71.8094	1.098612
Or_C_Pneum	Pneumoridae	17	1	71.8094	2.833213
Or_C_Trigo	Trigonopterygidae	16	1	67.0928	2.772589
Or_C_Pyrgo	Pyrgomorphidae	455	1	94.4352	6.120297
Or_C_Pamph	Pamphagidae	448	1	76.3679	6.104793
Or_C_Lentu	Lentulidae	35	1	56.8375	3.555348
Or_C_Acrid	Acrididae	6016	1	43.1799	8.702178
Or_C_Romal	Romaleidae	465	1	43.1799	6.142037

Clade abbrev.	div. e=0	div. e=0.5	div. e=0.9
Or_E_Grylli	0.04327869	0.03972868	0.03149202
Or_E_Myrme	0.01602837	0.01159345	0.00409009
Or_E_Gryllot	0.03549672	0.03023063	0.01841262
Or_E_Steno	0.02570410	0.02062796	0.01009227
Or_E_Gryllac	0.07327163	0.06549239	0.04752320
Or_E_Anost	0.05992316	0.05218173	0.03450670
Or_E_Rhaph	0.05223057	0.04641629	0.03301075
Or_E_Tetti	0.11344368	0.10453896	0.08387352
Or_E_Proph	0.05477333	0.04604645	0.02671980

Or_C_Cylin	0.01310575	0.01011588	0.00433122
Or_C_Trida	0.02625119	0.02256523	0.01422651
Or_C_Theri	0.04590958	0.04004824	0.02665167
Or_C_Tetri	0.06066964	0.05477652	0.04113171
Or_C_Eusch	0.04090116	0.03554406	0.02332874
Or_C_Eumas	0.05746396	0.05018535	0.03353825
Or_C_Episa	0.06567314	0.05497245	0.03139063
Or_C_Choro	0.08014234	0.06929520	0.04464626
Or_C_Prosc	0.03898292	0.03398119	0.02255431
Or_C_Tanao	0.01529900	0.00965260	0.00253897
Or_C_Pneum	0.03945463	0.03059801	0.01330622
Or_C_Trigo	0.04132468	0.03189711	0.01365707
Or_C_Pyrgo	0.06480949	0.05749282	0.04063421
Or_C_Pamph	0.07993926	0.07089203	0.05004849
Or_C_Lentu	0.06255286	0.05085325	0.02606738
Or_C_Acrid	0.20153307	0.18548438	0.14824230
Or_C_Romal	0.14224297	0.12624018	0.08936154

Supplementary Table 20. Full results of PGLS analyses of herbivory and diversification in Orthoptera. Using three values for relative extinction rates (e=0, 0.5, and 0.9).

Variables	r^2	*P*
Diversification rate (e=0) ~ herbivory	0.0620	0.2251
Diversification rate (e=0.5) ~ herbivory	0.0586	0.2443
Diversification rate (e=0.9) ~ herbivory	0.0506	0.2965
Ln-species ~ herbivory	0.0238	0.5642
Ln-species ~ diversification rate (e=0)	0.6146	<0.0001
Ln-species ~ diversification rate (e=0.5)	0.6434	<0.0001
Ln-species ~ diversification rate (e=0.9)	0.6902	<0.0001
Ln-species ~ age	0.0008	0.9811

Supplementary Table 21. Comparison of mean diversification rates between herbivorous and non-herbivorous clades. Including comparisons both among insect orders (using three phylogenies) and within two insect orders that show significant relationships between herbivory and diversification. Epsilon (e) is the assumed ratio of speciation to extinction. Rates are in species per million years.

Tree	Epsilon	Clade type	Mean rates	Ratio
Insect orders	e=0	Non-herbivorous	0.028	1.50
Misof tree		Herbivorous	0.042	
	e=0.5	Non-herbivorous	0.025	1.56
		Herbivorous	0.039	
	e=0.9	Non-herbivorous	0.018	2.20
		Herbivorous	0.039	
Insect orders	e=0	Non-herbivorous	0.021	1.5
Rainford tree		Herbivorous	0.032	
	e=0.5	Non-herbivorous	0.019	1.6
		Herbivorous	0.030	
	e=0.9	Non-herbivorous	0.014	1.8
		Herbivorous	0.025	
Insect orders	e=0	Non-herbivorous	0.027	1.5
This study		Herbivorous	0.041	

	e=0.5	Non-herbivorous	0.024	1.6
		Herbivorous	0.039	
	e=0.9	Non-herbivorous	0.018	1.8
		Herbivorous	0.032	
Diptera	e=0	Non-herbivorous	0.064	1.8
		Herbivorous	0.117	
	e=0.5	Non-herbivorous	0.055	1.8
		Herbivorous	0.101	
	e=0.9	Non-herbivorous	0.035	2.1
		Herbivorous	0.074	
Hemiptera	e=0	Non-herbivorous	0.029	1.4
		Herbivorous	0.042	
	e=0.5	Non-herbivorous	0.025	1.5
		Herbivorous	0.037	
	e=0.9	Non-herbivorous	0.016	1.6
		Herbivorous	0.026	

Supplementary Table 22. Data used in sister-clade comparisons. Including comparisons between orders and within orders. (H) = herbivorous clade (or clade with a higher proportion of herbivorous species). (N) = non-herbivorous clade (or clade with a smaller proportion of non-herbivorous species).

Clades	Richness of herbivorous clade	Richness of non-herbivorous clade
**Between-order comparisons**		
Phasmatodea (H) vs. Embioptera (N)	3046	457
Lepidoptera (H) vs. Trichoptera (N)	158423	14548
Diptera (H) vs. Siphonaptera (N)	156774	2082
Hemiptera (H) vs. Thysanoptera (N)	102183	5938
Coleoptera (H) vs. Strepsiptera	389487	613
**Coleoptera**		
Oedemerinae (H) vs. Boridae (N)	1500	4
Aderidae (H) vs. Ischaliinae (N)	1000	50
Mordellinae (H) vs. Melittommatinae (N) + Hylecoetinae (N)	1500	30
Byturidae (H) vs. Biphyllidae (N)	16	195
Epilachninae (H) vs. Scymninae (N) + Chilocorinae (N) + Coccidulinae (N)	1051	3900

Corticariinae (H) vs. Latridiinae (N)	850	200
Phalacrinae (H) vs. Laemophloeidae (N) + Propalticidae (N)	504	435
Languriinae (H) vs. Xenoscelinae (N)	800	100
Zeugophorinae (H) vs. Parandrinae (N	55	30
Orsodacninae (H) vs. Spondylidinae (N)	10	100
Cerambycinae (H) vs. Prioninae (N)	1000	1600
Choraginae (H) vs. Anthribinae (N)	350	2650
Cycladinae (H) vs. Scotylinae (N)	38	5800
Cossoninae (H) vs. Platypodinae (N)	1666	1500
Elaterinae (H) vs. Thylacosterninae (N)	3500	50
Agrypninae (H) vs. Drilidae (N)	2300	20
Dascillinae (H) vs. Rhipiceridae (N)	80	57
Trachyinae (H) + Julodinae (H)	14000	349
Orphninae (H) vs. Melolonthinae (N)	156	10737
Harpalinae (H) vs. Paussinae (N)	20000	724
Haliplidae (H) + Notomicrinae (H) + Noterinae (H) vs. Sphanglerogyrinae (N) + Gyrininae (N)	448	1101
**Diptera**		
Tipulidae (H) vs. Trichoceridae (N)	15204	160
Cecidomyiidae (H) + Lygistorrhynidae (N)	6059	30
Bibionidae (H) + Pachyneuridae (N)	751	5

Opomyzidae (H) vs. Sepsidae (N) + Acartophthalmidae (N)	61	379
Tephritidae (H) vs. Tachiniscidae (N) + Pyrgotidae (N)	4632	354
Chloropidae (H) vs. Milchiidae (N)	2863	276
Diopsidae (H) vs. Marginidae (N) + Nanodastiidae (N) + Canacidae (N)	183	320
Neriidae (H) vs. Pseudopomyzidae (N)	111	34
Celyphidae (H) + Lauxaniidae (H) vs. Chamaemyiidae (N)	2010	349
Fergusoninidae (H) + Asteiidae (H) vs. Xenasteiidae (N) + Australimyzidae (N)	161	22
Pallopoteridae (H) vs. Neurochatidae (N)	66	20
Psillidae (H) vs. Syringogastridae (N)	320	10
Agromyzidae (H) vs. Sphaeroceridae (N)	2800	1555
Drosophiliidae (H) vs. Braulidae (N) + Chryptochaetidae (N)	3944	40
Anthomyiidae (H) + Scathophagidae (H) vs. Rhinophoridae (N) + Calliphoridae (N) + Tachinidae (N) + Oestridae (N) + Sarcophagidae (N)	2289	14934
Phoridae (H) vs. Ironomyiidae (N)	4042	3
Dolichopodidae (H) vs. Hybotidae (N) + Empididae (N)	7151	4820

Stratiomyidae (H) vs. Xylomyidae (N)	2660	134
Ceratopogonidae (H) + Chironomidae (H) vs. Thaumalidae (N) + Simuliidae (N)	12573	2252
**Hemiptera**		
Thaumastocoridae (H) vs. Plokiophilidae (N) + Lycetocoridae (N) + Anthocoridae (N) + Cimicidae (N)	19	733
Berytidae (H) vs. Reduviidae (N)	100	6420
Cydnidae (H) vs. Canopidae (N)	617	8
Colobathristidae (H) vs. Termitaphididae (N)	90	1
**Hymenoptera**		
Cynipidae (H) vs. Figitidae (N)	1000	1500
Agaonidae (H) vs. Mymaridae (N)	757	1424
Torymidae (H) vs. Aphelinidae (N) + Perilampidae (N)	986	1445
Eurytomidae (H) vs. Chalicididae (N)	1424	1464
**Orthoptera**		
Gryllotalpidae (H) vs. Myrmecophilidae (N)	100	8
Anostostomatidae (H) vs. Gryllacrididae (N)	206	675
Tettigoniidae (H) vs. Prophalangopsidae (N)	6827	71

Supplementary Methods

Phylogeny reconstruction

We used three time-calibrated phylogenies among insect orders for this study. The use of these trees helped to address the robustness of the results to reasonable variation in topology and branch lengths. First, we used the recently published tree of Misof and colleagues[3] (their Fig. 1), which is based on sampling of 1,478 nuclear genes and 103 insect species. Second, we used the recently published tree of Rainford and colleagues[4] (their Fig. 1), which is based on only eight genes but extensive taxon sampling (874 hexapod species). Third, we generated our own time-calibrated tree for this purpose (prior to the publication of the other two studies), using 11 genes and 77 ingroup taxa. In the two sections that follow, we describe how we generated this tree.

Several recent studies have addressed higher-level insect phylogeny, but most have lacked one or more orders[7–11] or were comprehensive but not time-calibrated[12–13]. Therefore, we generated a time-calibrated phylogeny that included representatives of all insect orders, using published sequence data.

We initially focused on the dataset of Sasaki and colleagues[43], to which we added genes and taxa from the dataset of McKenna and Farrell[8]. The dataset of Sasaki and colleagues[13] includes the data of Ishiwata and colleagues[12] and consists of three very long nuclear genes: those encoding the catalytic subunit of DNA polymerase delta (*DPD1*) and the two largest subunits of RNA polymerase II (*RPB1*, *RPB2*). The analysis of McKenna and Farrell[8] included data from seven single-copy nuclear protein-coding genes and two nuclear ribosomal genes (*18S, 28S*) and included much of the data used in previous studies of holometabolous insect phylogeny (e.g. Wiegmann and colleagues[7]).

The protein coding genes were elongation factor-1a (*EF-1a*), alanyl-tRNA synthetase (*AATS*), carbamoylphosphate synthase domain (*CAD*), 6-phosphogluconate dehydrogenase (*PGD*), sans fille (*SNF*), triosephosphate isomerase (*TPI*), and RNA polymerase II (*RPB2*). These genes are widely used in insect phylogenetics in general. The two datasets overlapped for one gene. Overall, a total of 11 genes were included: *18S, 28S, AATS, DPD1, CAD, EF-1a, PGD, RPB1, RPB2, SNF,* and *TPI.*

We started with the dataset of Sasaki and colleagues[13], which included 64 arthropod taxa, and then added genes and taxa, primarily from the matrix of McKenna and Farrell[8]. We included a total of 77 ingroup taxa (Hexapoda) and 11 outgroup taxa. Outgroup taxa consisted of non-hexapod arthropods, including Chelicerata (Ixodida, Opiliones), Crustacea (sensu lato; including Anostraca, Cladocera, Cyclopoida, Decapoda, Nectiopoda, Notostraca, Podocopida, Stomatopoda), and Myriopoda (Chilopoda). Note that we consider Entognatha (Collembola, Diplura, Protura) as part of our ingroup (Hexapoda, although we often refer to this clade as "insects", following standard practice). We then searched for additional sequences for each family and each gene. In some cases, we combined data from congeners and confamilials in order to make each taxon as complete as possible. Additional sequences were identified through BLASTn or tBLASTn. Data were downloaded from GenBank, genomic databases (Flybase, Fleabase), or publicly available genomes. On average, each taxon in the final matrix had data for 66.8% of the 11 genes. GenBank numbers and the genes and species sampled for each taxon are given in Supplementary Table 1.

The final matrix included some missing data (e.g. a given species lacked data for a given gene). However, many studies suggest that the mere presence of missing data

need not prevent genes and taxa from increasing phylogenetic accuracy through their inclusion[14–16]. Recent analyses suggest that molecular dating analyses can also be highly robust to missing data, specifically those using BEAST[17].

RNA sequences were aligned using MUSCLE[18] in Geneious[19] 6.1.7. Coding sequences of protein coding genes were aligned via the translation align option in Geneious 6.1.7. Alignments were cleaned by removing sites that were divergent or ambiguously aligned in gblocks[20] 0.91b. Gblocks was run with default settings, except that the maximum number of contiguous nonconserved positions was set to four and the positions with >50% gaps were ignored. Nucleotide alignments of the protein coding genes were cleaned in codon format, RNA sequences were cleaned in DNA format, and translated alignments were cleaned in protein format.

Phylogenetic analyses were conducted on three concatenated datasets: (1) a nucleotide dataset with all codon positions included, (2) a nucleotide dataset with only first and second codon positions included, and (3) a dataset of amino-acid sequences. In all three datasets, the nucleotide sequences of the genes *DPD1*, *RPB1* and *RPB2* were translated into amino-acid sequences, since this was found to be the optimal format for these three loci for inferring the phylogeny of Hexapoda in previous studies[12,13]. Gene matrices were concatenated and initially partitioned by gene and codon position. The final optimal partitioning strategy and model of evolution for both nucleotide and protein datasets were identified via PartitionFinder[21] v1.1.1 under default settings (e.g. Bayesian information criterion, greedy algorithm). The overall best-fitting partitions and models are described in Supplementary Table 2. Since *DPD1*, *RPB1*, and *RPB2* were always treated as amino acid sequences, they could not be included with the concatenated

nucleotide sequences in PartitionFinder. The partitions and models for these genes were determined in two ways: for datasets one and two these genes were analyzed in PartitionFinder Protein[21] as their own matrix, and for dataset three these genes were analyzed in PartitionFinder Protein as part of the entire concatenated dataset of amino acid sequences. Both analyses identified the same partitions (i.e. each locus with a separate partition) and substitution models for *DPD1*, *RPB1,* and *RPB2*, which are shown in Supplementary Table 2.

The initial topology for each dataset was inferred using maximum likelihood, using RAxML[22] HPC v8.0.9. The optimal partitioning strategy and models (identified from PartitionFinder) were applied to each dataset. The GTRGAMMA model of substitution was applied to nucleotide partitions. We used the "f a" option to simultaneously search for the best-fitting tree and assess bootstrap support. For each analysis, we ran 1000 bootstrap replicates combined with multiple searches for the optimal tree.

Overall, we found that the tree based on all nucleotide data (dataset 1) was most congruent with prior molecular and morphological analyses, as summarized by Trautwein and colleagues[23]. Indeed, the tree estimated from this analysis (Supplementary Figure 1) was almost identical to that summarized in Figure 1 of that paper. An important exception was that our placement of Zygentoma in this tree differed from other recent and traditional estimates. Specifically, our estimates placed this taxon as sister to Ephemeroptera + Odonota, whereas other studies (including that by Misof and colleagues[3]) consistently place Ephemeroptera as sister to Pterygota (Paleoptera [Ephemeroptera + Odonota]) and Neoptera). In other words, the weakly supported

placement of Zygentoma was inconsistent with monophyly of Pterygota. Therefore, we constrained Zygentoma to be the sister group to Pterygota in our dating analyses (see below). The estimated tree is shown in Supplementary Figure 1.

There were also some differences between this topology and that estimated by Misof and colleagues[3]. These included the following: (1) placement of Diplura and Protura as sister taxa in our tree (and sister to Collembola; see also Trautwein and colleagues[23]), whereas Misof and colleagues[3] place Protura and Collembola as sister taxa, with Diplura as sister to Insecta, (2) placement of Dermaptera and Plecoptera as sister taxa, whereas Misof and colleagues[3] place Dermaptera with Zoraptera and place Plecoptera as sister to other Polyneoptera (exclusive of Dermaptera and Zoraptera), (3) placement of Orthoptera as sister to Blattodea, Mantodea, and Zoraptera in our tree, whereas Misof and colleagues[3] place Orthoptera as sister to the clade including Blattodea, Mantodea, Embioptera, Phasmatodea, Mantophasmatodea, and Grylloblattodea, (4) placement of Psocodea in our tree as sister to Thysanoptera + Hemiptera (see also Trautwein and colleagues[23]), whereas Misof and colleagues[11] place Psocodea as sister to Holometabola, and (5) placement of Megaloptera as sister to Neuroptera + Raphidioptera, whereas Misof and colleagues[3] place Raphidioptera as sister to Neuroptera + Megaloptera.

The tree of Rainford and colleagues[4] is similar to our tree and to other recent hypotheses, and is almost identical to the consensus topology shown by Trautwein and colleagues[23]. One notable difference is that Rainford and colleagues[4], like Misof and colleagues[3], place Raphidioptera as sister to Neuroptera + Megaloptera, whereas our tree places Megaloptera as sister to Neuroptera + Raphidioptera and Trautwein and

colleagues[23] place Neuroptera as sister to Megaloptera + Raphidioptera. Furthermore, there are some differences in relationships among the orders of Polyneoptera. Specifically, Rainford and colleagues[4] place Orthoptera as the sister group to all other Polyneoptera exclusive of Dermaptera and Plecoptera, whereas our tree places Orthoptera as sister to the clade of Zoraptera, Blattodea, and Mantodea.

Time-calibration

We estimated a time-calibrated phylogeny using the uncorrelated lognormal approach in BEAST version 2.1.3 (Bouckaert and colleagues[24]). We used amino-acid sequences (instead of nucleotide sequences) to reduce both rates of change and variation in rates among sites. Preliminary analyses using nucleotide data gave problematic results (e.g. species in different orders sharing extremely recent divergent dates). Therefore, we included the nine nuclear protein-coding genes and excluded the two ribosomal genes (note that BEAST did not allow mixing amino-acid and nucleotide sequence data). We used the best-fitting models and partitions selected by PartitionFinder for these genes, in a separate analysis dedicated to this restricted set of genes. The best-fitting model had six partitions (Supplementary Table 3). We performed analyses with the tree and clock models linked across partitions, but with site models unlinked. We used the default of four rate categories for the gamma parameter for rate variation among sites. For each partition, we used estimated substitution rates, gamma shape parameter, and proportion of invariant sites (initially set to 0.5). We used the standard relaxed clock lognormal model for the clock model. To make estimation of divergence dates more straightforward, we constrained all aspects of the topology following our preferred estimate (maximum

likelihood estimate from the mixed nucleotide-AA sequence data). However, as noted above, we constrained Zygentoma to be the sister of Pterygota. The overall topology was therefore highly consistent with previous molecular and morphological estimates of insect phylogeny (reviewed by Trautwein and colleagues[23]).

We used a total of 15 fossil calibration points (see below). These were treated as minimum ages, with a lognormal distribution on the prior, and the fossil age treated as the offset. We used a mean of 5 Myr (Million years) and a standard deviation of 1 Myr. This combination give a prior distribution that had its median at 3 Myr older than the offset, and a 95% prior distribution extending from 1 to 15 Myr older than the offset age. The choice of these specific numbers is somewhat arbitrary, but it should nevertheless reflect the idea that clades can be older than their fossil calibration points, but may be more likely to be only somewhat older. Note also that the prior distribution does not directly set the final clade ages.

We ran the dataset five times, for ~15,000,000 generations each. This number of generations corresponded to the maximum number of generations that could be run in the time limits allowed by our computer cluster. We used Tracer[25] (version 1.6) to assess stationarity of each replicate. Among the five replicate analyses, we included three, and excluded two. One of the excluded replicates had highly divergent date estimates relative to the others, whereas both excluded replicates had posterior estimates that differed substantially from the other three replicates. Therefore, we chose to exclude these two replicates. We combined the remaining three runs using LogCombiner, after removing the first five million generations of each run as burnin. Burnin for each run was determined by inspecting the plot of likelihoods over time in Tracer v1.6. After

combining the three runs, we found that ESS for the likelihood was >200. Although ESS for the dates for some clades was less than 200, date estimates for these clades were highly congruent across the three replicates, strongly suggesting that these date estimates were not artifacts of stochastic sampling. BEAST inference was parallelized via the BEAGLE library[26]. The estimated tree was the maximum credible tree with mean branch lengths (Supplementary Figure 2).

The estimated dates were generally similar to those estimated in recent studies of insect divergence times, but for some clades were substantially older. For example, dates estimated here are sometimes older but generally similar to those estimated by Wiegmann and colleagues[7], including Holometabola (~350 Ma versus ~375 Ma here), Coleoptera + Strepsiptera (~275 Ma vs. 309 Ma), Lepidoptera + Trichoptera (~230 Ma vs. 193 Ma), Diptera + Mecoptera + Siphanoptera (~280 Ma vs. ~250 Ma), and Neuroptera + Raphidioptera + Megaloptera (~250 Ma vs. ~245 Ma). Relative to those of Rota-Stabelli and colleagues[9] (their Fig. 1) our age estimate for Hexapoda is considerably older (~490 Ma vs. 590 Ma here) but very similar for Pterygota (~425 Ma for both). Relative to those of Thomas and colleagues[10], our age estimate for Palaeoptera is similar (~350 Ma vs. ~345 Ma here) but somewhat older for Neoptera (~360 Ma vs. 402 Ma here) and Pterygota (~380 Ma vs. ~426 Ma here). Relative to those of Wheat and Wahlberg[11], our estimated dates are again somewhat older. For example, they estimate Pterygota at ~380 Ma (426 Ma here), Palaeoptera at ~325 Ma (~345 Ma here), Neoptera at ~350 Ma (~402 Ma here), and Holometabola at ~310 Ma (375 Ma here). Relative to those of Misof and colleagues[3], our estimates for the deepest clades are older, but are more similar for shallower clades. For example they estimate Hexapoda at 479 Ma (589 Ma here),

Pterygota at 406 Ma (426 Ma here), Palaeoptera at 362 Ma (344 Ma here), Neoptera at 387 Ma (402 Ma here), and Holometabola at 345 Ma (375 Ma here). Relative to those of Rainford and colleagues[4] our estimates are again older for the deepest clades but are younger for shallower clades. For example they estimate Hexapoda at 478 Ma (589 Ma here), Pterygota at 441 Ma (426 Ma here), Palaeoptera at 373 Ma (344 Ma here), Neoptera at 430 Ma (402 Ma here), and Holometabola at 390 Ma (375 Ma here). The estimates of Rainford and colleagues[4] are generally similar to those of Misof and colleagues[3], but the former tend to be somewhat older than the latter.

In summary our age estimates here are broadly similar to those in earlier studies for more recent clades (but somewhat older for deeper clades), and age estimates vary considerably among these studies as well. We also note that some of the age estimates in previous studies are younger than the minimum fossil ages used here (suggesting that these younger age estimates may be incorrect).

Fossil calibration points

To find fossil calibration points, we reviewed divergence times for insect and hexapod orders provided by Rota-Stabelli and colleagues[9] and Thomas and colleagues[10], as well as Benton and colleagues[27]. From these sources, we took the oldest fossil that could be confidently assigned to each clade, and used the minimum age of the stratum (based on Gradstein and colleagues[28]) in which it appears as the minimum age of the clade. We focused on crown-group clades whenever possible, based on our phylogenetic sampling.

We used the following 15 calibration points, listed alphabetically by clade name.

1) Blattodea

Rota-Stabelli and colleagues[9] used the age of *Gondwablatta* (from Vršansk[29]) to yield a minimum age of 124 Ma for Blatttaria (referred to as Blattodea here), and a maximum of 308 Ma. However, in a more recent paper, Vršansk and Ansorge[30] described a somewhat older blattodean fossil from the Lower Toarcian, which they date fom (175.6—183.0 Ma). Gradstein and colleagues[28] date the Toarcian from 174–183 Ma. We therefore use 174 Ma for the minimum age of the blattodean stem group. In our tree, the stem of Blattodea corresponds to clade of Blattidae + Rhinotermitidae within Blattodea.

Crown group Blattidae + Rhinotermitidae minimum age = 174 Ma.

2) Coleoptera

Rota-Stabelli and colleagues[9] used 307 Ma as the date for the earliest coleopteran, based on an undescribed specimen from from the middle Carboniferous Mazon Creek fauna of Illinois, USA[27]. Benton and colleagues[27] justified dating this fauna at a minimum of 307 Ma. Benton and colleagues[27] also state that the oldest described coleopteran is *Pseudomerope gallei* from the Asselian (minimum age = 296 Ma). In our tree, stem Coleoptera corresponds to the clade of Coleoptera + Strepsiptera. We note that it is possible that Strepsiptera is nested inside of Coleoptera (but see[3,4]). In this case, it might be that the stem group we use underestimates the age of this clade, but this clade is already among the oldest ones used here.

Crown group Coleoptera + Strepsiptera minimum age = 307 Ma.

3) Collembola

Both Rota-Stabelli and colleagues[9] and Thomas and colleagues[10] used the same fossil to date the crown-group age of Collembolla. Specifically, the Devonian fossil *Rhyniella* is thought to belong to Isotomidae, an extant collembolan family[1]. Given that our tree includes this family, we can treat this as a crown-group age for Collembola. Grimaldi and Engel[1] noted that this fossil is from the Devonian, Pragian–Emsian, with an age of 412.3–391.9 Ma. However, Gradstein and colleagues[28] give 393 Ma as the minimum age of the Emsian. In our tree, relationships among the three families of collembolans incuded are uncertain. Therefore, we simply use this date for the overall crown group of Collembola.

Crown group Collembola (Bourletiellidae, Isotomidae, Tomoceridae) minimum age = 393 Ma.

4) Diplura

Rota-Stabelli and colleagues[9] used a minimum age of 111 Ma for Diplura, based on the earliest Japygidae, *Ferrojapyx vivax*, known from the Lower Cretaceous Aptian[30]. However, using the results of Gradstein and colleagues[28], the minimum age for this period should be 113 Ma. According to Wilson and Martill[31], an older dipluran is known from the Late Carboniferous but its assignment to Diplura is questionable. Our sampling of diplurans includes Japygidae and Campodeidae. Therefore, we use this fossil to date the crown group of Diplura.

Crown group Diplura minimum age = 113 Ma.

5) Diptera

Benton and colleagues[27] give the minimum date for crown Diptera as 238.5 Ma, corresponding to the split of Culicidae (mosquitoes) and Drosophilidae (vinegar flies). We use 238.5 Ma here. In our tree, Culicidae, a nemotoceran lineage, is sister to other sampled dipteran taxa. Therefore, this date corresponds to the crown group of Diptera in our tree.

Crown group Diptera minimum age = 238.5 Ma.

6) Hemiptera (crown)

Rota-Stabelli and colleagues[9] used the earliest age for Hempitera from Benton and colleagues[27]. Benton and colleagues[27] gave the minimum age for crown-group Hemiptera (spanning reduviids and aphids) based on the fossil taxon *Lufengnacta* from the Yipinglang Coal Series (Late Triassic: Rhaetian-Norian). Based on Gradstein and colleagues[28], the end of the Rhaetian is 201 Ma. Given our sample of hemipteran taxa, the clade spanning reduviids and aphids corresponds to crown group Hemiptera in our tree.

Crown group Hemiptera minimum age = 201 Ma.

7) Hemiptera (stem)

Thomas and colleagues[10] gave the minimum age of Hemiptera as 251 Ma, based on the fossil taxon *Paraknightia magnifica* (Heteroptera) from Newcastle Coal Measure, Australia from the Late Permian, 260–251 Ma. Using the ages from Gradstein and colleagues[28], we date this to 252 Ma. Given that this taxon may not fall confidently within a living hemipteran clade, we use it to date the stem age of Hemiptera, the age of

the split between Hemiptera and its putative sister group, Thysanoptera (although this relationship is not strongly supported).

Hemiptera + Thysanoptera minimum age = 252 Ma.

8) Hymenoptera

Benton and colleagues[27] gave the minimum age for the split between *Apis* (Apidae) and *Nasonia* (Pteromalidae) as 152 Ma (crown-group Apocrita). We use 152 Ma for the crown-group age of Hymenoptera, given that in our tree the ancestor of *Apis* (Apidae) and *Nasonia* (Pteromalidae) corresponds to the crown group of Hymenoptera. We do not use older fossils for Hymenoptera because their placement within the order is uncertain, and they give stem group ages (e.g. 215 Ma) that are too young relative to the ages of other orders in this clade (e.g. Coleoptera at 307 Mya).

Crown group Hymenoptera minimum age = 152 Ma.

9) Lepidoptera

Rota-Stabelli and colleagues[9] gave a minimum age of 99 Ma for Lepidoptera, based on leaf mines from Gracillariidae[32]. Grimaldi and Engel[1] and Zhang and colleagues[33] suggested that the oldest unequivocal fossil lepidopteran was *Archaeolepis mane*[34] from the Early Jurassic, about 190 million years ago (Ma). However, it is not clear that this fossil belongs to the crown group of the lepidopteran species that we have sampled. Therefore, we used this fossil to date the stem group. In our tree, Lepidoptera is strongly supported as the sister group to Trichoptera. Therefore, we use this calibration point for the minimum age of the Lepidoptera+Trichoptera split as 190 Ma.

Crown group Lepidoptera + Trichoptera minimum age = 190 Ma.

10) Mecoptera

Wiegmann and colleagues[6] listed 220 Ma as the minimum age of Mecoptera, based on the fossil mecopteran *Thaumatomerope neuropteroides*[35]. However, it is unclear if this fossil is within the crown group of living clades. In our tree, Mecoptera is strongly supported as the sister group to Siphonaptera. Therefore, the age of this clade is at least 220 Ma. This is largely consistent with Fig. 12.1 of Grimaldi and Engel[1].

Crown group Mecoptera + Siphonaptera minimum age = 220 Ma.

11) Odonata

Rota-Stabelli and colleagues[9] and Thomas and colleagues[10] agreed that the earliest Odonata fossil is in Suborder Zygoptera, family Triassolestidae, from the Triassic (251–201.6), with a minimum age of 201.6 Ma. Here, we use 202 Ma for the crown group of Odonota, given that our sampling includes both Zygoptera (Calopterygidae) and Epiproctra (Epiophleberoptera; Epiophlebiidae).

Crown group Odonata minimum age = 202 Ma.

12) Orthoptera

Rota-Stabelli and colleagues[9] report the earliest orthopteran to be the ensiferan *Iasvia* (Bethoux and colleagues[36]), with a minimum age of 267 Ma. In contrast, Thomas and colleagues[10] report the oldest Ensifera to be *Raphogla rubra* (oldest representative of modern Ensifera), Lodeve Basin, France, Late Permian[1], where the Late Permian spans

260–252 Ma. We therefore use *Iasvia*, which Bethoux and colleagues[36] date to the Roadian period. This period spans from 269–272 Ma according to Gradstein and colleagues[28]. Our sampling of orthopterans includes both Ensifera (Gryllidae, Tettigoniidae) and Caelifera (Acridoidea, Acrididae). It is unclear if *Iasvia* is within the crown group of Ensifera. Therefore, we use this fossil to date the ensiferan stem group. Given our sampling and tree, this corresponds to the crown group of Orthoptera.

Crown group Orthoptera minimum age = 269 Ma.

13) Phthiraptera (part of Psocodea)

Phthiraptera has a minimum age of 100 Ma based on *Cretoscelis burmitica*, from the Upper Albian-Cenomonian[37]. Further, *Saurodectes vrsanskyi* from the Zaza Formation of Baissa, Siberia (Early Cretaceous, ca. 130 Ma) is a “putative louse”, according to Grimaldi and Engel[37]. In our tree, Pthiraptera and Psocoptera are sister taxa. Therefore, we use this older calibration to date the minimum age of the split between these clades. Although Pthiraptera is most likely nested inside of Psocoptera (see above), this should not adversely affect this calibration, since we are merely dating the age of the split between our samples of these two groups.

Phthiraptera + Psocoptera minimum age = 130 Ma.

14) Plecoptera

Thomas and colleagues[10] identified the oldest plecopterans as belonging to the families Lemmatophoridae, Liomopteridae, and Probnidae from the Permian[1], corresponding to 299–252 Ma using the time scale of Gradstein and colleagues[28]. It is not clear if these

families are within the crown-group clade, and in our analysis, Plecoptera is represented by a single terminal taxon. Therefore, we use this fossil to date the stem group of Plecoptera. In our tree, Plecoptera and Dermaptera are supported as sister taxa. Therefore, we use 252 Ma as minimum age for this clade.

Plecoptera + Dermaptera minimum age = 252 Ma.

15) Pterygota

Benton and colleagues[27] identified the oldest insect fossil as *Rhyniognatha hirtsi* from the Early Devonian Rhynie Chert of northeast Scotland[38], which are dated to the base of Pragian period (the Pragian–Emsian border; see above for Collembola). Gradstein and colleagues[28] gave 393 Ma as the minimum age of the Emsian. According to Engel and Grimaldi[38] and Grimaldi and Engel[1], this taxon belongs to Pterygota, including Ephemeroptera, Odonota, and all other insects (excluding Archeognatha and Zygentoma). We therefore use 393 Ma as the minimum age for this clade. Note that Thomas and colleagues[10] calibrated this clade to a minimum of 299 Ma, based on presence of all groups by the Late Carboniferous, 318–299 Ma, as reported by Grimaldi and Engel[1]. However, this date is younger than our calibration point for Coleoptera + Strepsiptera, which belongs to Pterygota.

Crown group Pterygota minimum age = 393 Ma.

Excluded calibration points

We also excluded several calibration points that were used by previous authors. We give our justification for not using these points below, again going alphabetically by names of orders and other clades.

1) Archaeognatha

Rota-Stabelli and colleagues[9] gave a minimum age of 383 Ma for this clade, whereas Thomas and colleagues[10] listed the oldest archeognathan as *Triassomachilis uralensis*, from the Triassic of Russa (251–201.6 Ma), based on Grimaldi and Engel[1]. However, both dates are problematic for our purposes, given that we have only a single family of Archaeognatha represented in our tree (Machilidae), and can therefore only calibrate the stem group. Moreover, given the relatively basal placement of Archaeognatha in our tree, even the older date may be too young, given the age of other, more derived insect clades for which we have calibrations (e.g. Coleoptera, Pterygota).

2) Dictyoptera (Blattaria, Mantodea, Isoptera)

Thomas and colleagues[10] used *Baissatermes lapideus* (Isoptera) and *Baissomantis* sp. (Mantodea) from the Zaza formation of Baissa, Siberia, Russia, which is in the Early Cretaceous, 145–99.6 Ma, based on Engel and colleagues[39]. Thus, they used a minimum age of 99.6 Ma for this clade. However, use of this fossil would be problematic for our study, given that we already have older fossil calibrations for Blattaria and Mantodea.

3) Ephemeroptera

Earliest fossils agreed on by Rota-Stabelli and colleagues[9] and Thomas and colleagues[10]

suggest that the earliest Ephemeroptera are *Fuyous gregarius* and *Shantous lacustris* from the Daohugou formation of China[40]. The age of the Daohugou is 153–165 Ma, according to Zhang and colleagues[41]. Therefore, 153 Ma could be used for the stem group age of Ephemeroptera. However, this stem age of Ephemeroptera is younger than crown-age of its sister group (Odonata: 202 Ma). Therefore, we do not use this calibration point.

4) Isoptera

Rota-Stabelli and colleagues[9] reported that the earliet isopteran is *Meiatermes*[42], with a minimum age of 124 Ma. However, since Blatteria and Isoptera are sister taxa, and since the stem age for Blatteria is 174 Mya (see above), this calibration point is redundant for our purposes here.

5) Paranoptera

Rota-Stabelli and colleagues[9] gave a minimum age of 284 Ma for this clade (including Hemiptera, Pscocoptera, Pthiraptera, and Thysanoptera), based on an undescribed archescytinid from the Bacov Beds[27]. However, the phylogenetic position of Archescytinidae is unclear[27]. Moreover, this date is similar to our calibration point for the Hemiptera + Thysanoptera clade (252 Ma). Therefore, we did not use this calibration point.

Estimating herbivory in clades

We initially considered three estimates of the proportion of herbivorous species in each insect order (Supplementary Table 4). Two were published estimates, and the third was

developed from our own survey of the literature. First, we used the proportion of phytophagous species for each order listed in Table 14.1 in Grimaldi and Engel[1]. The estimates of Grimaldi and Engel[1] were based on considering species as herbivorous if they feed on leaves, flowers, roots, stems, wood, seeds and fruits (p. 622). However, they do not include some taxa that feed on dead wood (xylophagous), such as termites (isopterans). Also, they did not include pollen-feeding taxa (e.g. bees) as herbivorous. Overall, the definition used by Grimaldi and Engel[1] followed that used by Mitter and colleagues[43], who restricted their definition to those species that feed on the living tissues of higher plants (Tracheophyta). However, Grimaldi and Engel[1] did not give references to support their estimates in each order.

We also considered estimates of the proportion of herbivorous species in each insect order from Table 1 of Hendrix[2]. These estimates were updated from those first proposed by Frost[44]. In applying these proportions to all currently recognized orders, we made a few minor modifications. First, although Hendrix[2] listed Isoptera as herbivorous we considered them xylophagous instead, following Grimaldi and Engel[1]. Since we included Isoptera in Blattodea, we considered Blattodea as having no herbivorous species. Second, Homoptera and Hemiptera were listed as separate orders by Hendrix[2]. Here, they are combined into the order Hemiptera and the proportion of herbivorous species reported in Hendrix[2] for each order were combined into one estimate for Hemiptera. Third, some orders were not listed in Hendrix[2]. We coded these missing orders following Grimaldi and Engel[1]. Most orders had highly similar proportions of herbivorous species between the two studies, with the exceptions of Isoptera and Thysanoptera.

We also developed our own estimates of the proportion of herbivorous speces in each order. We considered this necessary because the estimates of Grimaldi and Engel[1] are not supported by specific references or a description of how they were generated. Similarly, the estimates of Hendrix[2] are based on Frost[44], but the estimates of Frost[44] also lack specific documentation. Our major focus was on the nine orders in which some (but not all) species are known to be herbivorous. For our estimates, species were considered herbivorous if they were reported to feed primarily on living plant tissues during one or more life stages, or if previous authors considered them to be herbivorous without specifying a particular life stage for this dietary pattern

For orders with some herbivorous species, our general approach was to estimate whether each family within that order contained herbivorous species, and then use estimates of species richness in each family across the order to estimate the proportion of herbivorous species in that order. In general, we used the species richness estimates for each family listed in Table S1 of Rainford and colleagues[4]. However, in the case of Coleoptera and Diptera, we used species richness estimates from Hunt and colleagues[5] and Wiegmann and colleagues[6], respectively, since they accompanied the estimates of herbivory in these same clades by those authors (see below). For Thysanoptera, there were four families not listed by Rainford and colleagues[4], and these were included to obtain an overall estimate for the proportion of herbivorous species in this order (see below).

An important issue to note is that Rainford and colleagues[4] did not include all insect families (~80% included) and so this might potentially distort our estimates in some orders somewhat. Nevertheless, we reiterate that our estimates are very similar to

others. Furthermore, relatively few orders show signficant variation for herbivory. Most importantly, by comparing the total numbers of species included in our estimations to the total numbers in each of the five most variable orders (Supplementary Table 5), we can be more precise about our completeness. Specifically, for Coleoptera we included 345,428 of 389,487 species (88.7%), for Diptera 152,577 of 156,774 (97.3%), for Hemiptera 96,894 of 102,183 (94.8%), for Hymenoptera 141,014 of 153,088 (92.1%), and for Orthoptera 23,004 of 23,830 species (96.5%). Note that for Coleoptera, the higher taxa included by Hunt and colleagues[5] were estimated to encompass >95% of beetle species, and the lower proportion here may simply reflect the increase in species numbers since 2007 (i.e. >95% may be more accurate, given that most higher taxa were included).

For Coleoptera, Hunt and colleagues[5] included data on the species richness and diets of almost all families and subfamilies (their Table S8). We followed Hunt and colleagues[5] in only considering taxa feeding on living tracheophyte tissues to be herbivorous (i.e. xylophagy was considered distinct from herbivory). For some taxa, they listed multiple feeding types as present within that clade, without quantifying specific proportions. In these cases, we arbitrarily assumed that different feeding types were at equal frequencies within each family (e.g. if both herbivorous and predaceous habits are listed, we consider this clade to be 50% herbivorous). For six higher taxa, Hunt and colleagues[5] listed the feeding habits as unknown, and these taxa were not included in these analyses. Hunt and colleagues[5] noted that some higher taxa were not monophyletic, but their non-monophyly should have little impact on the overall estimates of herbivory across Coleoptera, and so we treated these taxa the same as the monophyletic higher taxa

for estimating overall herbivory proportions. The estimates used here are summarized in Supplementary Table 11.

For Collembola, we relied on species richness estimates for each family provided in Table S1 of Rainford and colleagues[4]. Fifty species are listed as herbivorous by Hendrix[2] and we subtracted this from the total number of species in the order (7806) to obtain an estimate of 7756 non-herbivorous members. Thus, rounded to the nearest whole number, 1% of collembolan species are herbivorous [1 – (7756/7806)].

For Diptera we used the data on species richness, and diets for dipteran families provided by Wiegmann and colleagues[6]. However, some modifications were necessary. First, we removed two taxa that were present in the tree but not in Fig. S3 of Wiegmann and colleagues[6]: Bolbomyiidae and Mythiocomidae. Second, the tree has "Pseudopomyzidae" where Cypselosomatidae is in Fig. S3. We assumed that Cypselosomatidae is the correct name for this taxon. This left a total of 142 families. Furthermore, Fig. S3 lacked estimates of species numbers for four families: Asteiidae, Chironomidae, Hilarimorphidae, and Xylophagidae. We used additional sources to estimate richness in these families (Asteiidae, Chironomidae, Xylophagidae; Courtney and colleagues[45]; Hilarimorphidae; Webb[46]). We followed Wiegmann and colleagues[6] and coded families as herbivorous if they were listed as herbivorous (phytophagous) in their Figure 1. However, we caution that they did not provide detailed information on how these taxa were coded, and it appears that they assumed that any occurrence of herbivory in a clade was sufficient for herbivory to be coded as present. The estimates used here are summarized in Supplementary Table 13.

For Hemiptera, we used species richness estimates for each family from Rainford and colleagues[4]. We coded all families within Auchenorrhyncha and Sternorrhyncha as 100% herbivorous, following Strong and colleagues[47]. Excluding these two taxa, there are 26 hemipteran families. For 21 of these families, the only diet noted in the review by Schuh and Slater[48] is herbivory (stated explicitly as being fixed across species in these taxa or assumed here to be fixed if no other diet is mentioned). These 21 families were coded as 100% herbivorous in our analyses. We dealt with the remaining five families as follows. (1) Aradidae was listed as herbivorous and mycophagous by Schuh and Slater[48] but with unknown frequencies of each diet. This family was arbitrarily considered to be 50% herbivorous (1000/2000). (2) Berytidae was listed as herbivorous by Schuh and Slater[48], with the exception of three genera known to contain predaceous species (*Jalysus*, *Neides* and *Berytinus*). We assumed that there are 5 described species of *Jalysus*, 1 of *Neides*, and 2 of *Berytinus*[49]. Assuming that all species in these genera are predaceous, there are eight non-herbivorous species in the family, and 92 species that are herbivorous, yielding 92% herbivorous species (92/100). (3) Within Pentatomidae, all species but those in Asophinae are herbivorous[48]. All species of Asophinae are thought to be predaceous[48], and there are estimated to be 357 species in this subfamily[48]. Subtracting the number of species of Asophinae from the overall number of species in Pentatomidae (from Rainford and colleagues[4]) yields an estimate of 92% herbivorous species (4143/4500). (4) Within Lygaedidae, most species are herbivorous[48], but some are considered predaceous (1 species[50]) or blood-feeding (54 species[51]). Assuming that these 55 species are non-herbivorous, we estimated that 99% of the 4400 species in Lygaedidae are herbivorous (4345/4400). (5) Within Miridae, herbivorous, predaceous, and

mycophagous species are listed as present but their relative frequencies are uncertain[48]. Therefore, we arbitrarily assumed that these diet types were equally divided among the ~10000 species in the family, yielding 33% herbivorous species (3333/10000). In summary, to estimate the total number of herbivorous species, for the 53 herbivorous families we coded as 100% herbivorous, we multiplied the species richness estimates for each herbivorous family by 100%. To this, we then added the estimates of herbivorous species for the five families that are variable for the presence of herbivory. In summary, our estimate of the total proportion of herbivorous species in Hemiptera is 78% (75965/96894). This estimate is very similar to that from Hendrix[2], who estimated 79% herbivorous species, and roughly similar to that from Grimaldi and Engel[1], who estimated 90%.

For Hymenoptera, we coded all families within the suborder Symphyta as herbivorous except for the xylophagous familes Sciricidae and Xyphydriidae (following Strong and colleages[47] and Mitter and colleagues[43]). For most other families, if any taxa were listed as herbivorous by Goulet and Huber[52] the family was coded as herbivorous. However, two of the largest hymenopteran families, Braconidae and Ichneumonidae each contain at least 20,000 species but each have only a few species that are herbivorous[52], and presumably less than 1% overall. Specifically, Braconidae contains two known herbivorous genera (*Allorhogas, Monitoriella*[53–55]) and Ichneumonidae contains only one (*Poecilocrypus*; Goulet and Huber[52]). Yet, in nearly all other families in which herbivory is present in Hymenoptera, 100% of known species are thought to be herbivorous[52]. Thus, for all familes with herbivorous species (except Braconidae and Ichneumonidae), we multiplied the species richness estimates for each family from Rainford and colleagues[4]

by 100% to estimate the overall number of herbivorous hymenopteran species. Our overall estimate is that 7% of hymenopteran species are herbivorous (10117/141014). This estimate is close to those from Hendrix[2] and Grimaldi and Engel[1], who estimated 10% and 12%, respectively.

Lepidoptera are known to have a high proportion of herbivorous species[1,2]. Pierce[56] listed the known non-herbivorous species of Lepidoptera. Fourteen families contain non-herbivorous species, totaling 293 non-herbivorous species, and all other lepidopteran species were considered herbivorous. Using the species numbers for each family from Rainford and colleagues[4], yields an estimate of 100% herbivory for Lepidoptera (155723/156016), after rounding. This is very similar to the values of 98 and 99% estimated by Hendrix[2] and Grimaldi and Engel[1].

For Orthoptera, we could not find any information on the feeding habits of Rhipipterygidae or Xyronotidae and so these species were excluded from the analyses. If any species was reported as being herbivorous in the remaining families, the family was coded as 100% herbivorous, following Preston-Mafham[57] and Capinera[58], and given the lack of more detailed data within each family. Using this strategy (and species richness values for each family from Rainford and colleagues[4]), the estimated proportion of herbivorous orthopteran species is 94% (21593/23004). This number is similar to proportions estimated by Hendrix[2] and Grimaldi and Engel[1] of 90% and 95%.

For Phasmatodea, all known species are herbivorous[59] and therefore we scored Phasmatodea as being 100% herbivorous.

For Thysanoptera, Hendrix[2] and Grimaldi and Engel[1] estimated very different proportions of herbivorous species (99% vs. 40%). Here, we used the most up-to-date

information provided by the definitive treatment of the systematics and ecology of Thysanoptera[60] to make our estimates. However, it is important to note that little information is available on the biology of most species and some of our estimates rest largely on the opinions of thysanopteran experts. We also augmented these rough estimates with more specific information when this was available. In addition to the four families included by Rainford and colleagues[4], we included Fauriellidae (little is known of their biology and there are five extant species), Melanthripidae (all 67 extant species originally placed in Aeolothripidae are herbivorous), Stenurothripidae (all six extant species are herbivorous) and Uzelothripidae (one extant species that feeds on fungi). These 74 (73 herbivorous) species were added to the 5875 species listed by Rainford and colleagues[4], yielding 5949 species. Aleotrhipidae is listed as being composed of approximately 50% herbivorous and 50% predaceous genera[60]. We therefore coded this family as 50% herbivorous. All but three species within Heterothripidae are listed as herbivorous (the three non-herbivorous species are ectoparasitic on Homoptera), and the proportion of herbivorous species within this family was estimated to be 96% (73/76 species from Rainford and colleagues[4]). According to ThripsWiki[60], 50% of species in Phlaeothripidae are saprophagous and mycophagous, whereas the remaining 50% are herbivorous species, with the exception of eight species (*Aleurodothrips fasciapennis*, *Haplothrips faurei*, *Karnyothrips flavipes*, *K. melaleucus*, *Leptothrips cassiae*, *L. macroocellatus*, *L. pini* and *L. mali)* that are predators of other arthropods[61,62]. Thus, among the 3532 species of Phlaeothripidae listed by Rainford and colleagues[4], 1766 (50%) are coded as non-herbivorous and 1758 are coded as herbivorous (1766 herbivorous species minus the eight predaceous species above) and the proportion of

herbivorous species within this family is estimated to be 49.77%. Thripidae are nearly all herbivorous, except for the 16 species of *Scolotrhips*, which are predaceous[60]. Thus, the total proportion herbivorous in Thripidae is estimated to be 99% (2050/2066). Totaling the estimated numbers of herbivorous species across these families yields an estimated proportion of 68% herbivorous species in Thysanoptera (4055/5949).

Apart from Thysanoptera, the estimates from our survey and those of Hendrix[2] and Grimaldi and Engel[1] are very similar (Supplementary Table 4). For brevity, we only present results from our analyses using the estimated proportions of herbivorous species from own survey, since these are the only ones based on explicit methodology and references. However, relationships between our estimates and the two others are extremely tight, and so any deviations should have minimal impact on our results (least-squares regression of our estimates vs. Grimaldi and Engel[1]: r^2=0.97, n=31; our estimates vs. Hendrix[2]: r^2=0.96, n = 31).

Impact of the evolutionary history of herbivory within orders

A potential concern in our analysis of herbivory and diversification rates across orders is that our estimates of herbivory within orders ignore the evolutionary history of herbivory within these orders. We argue that the number of origins herbivory within a clade is not relevant for understanding whether herbivory drives large-scale diversification patterns at the between-clade level, and that our methods are (and should be) agnostic on this topic. For example, in a species rich clade with 90% herbivorous species, this proportion could be achieved through a single origin of herbivory or through dozens of separate origins. Alternately, a clade with only 10% herbivorous species might also have this proportion

through a single origin or herbivory or through dozens. Interestingly, many of these extreme hypothetical possibilities (with regards to origins of herbivory and proportions of herbivorous species) are exemplified with empirical data for insect orders, including: (a) a single origin of herbivory and a large proportion of herbivorous species (e.g. Lepidoptera), (b) many origins of herbivory and a small proportion of herbivorous species (e.g. Coleoptera[5], Diptera[6]), (c) a seemingly limited number of origins of herbivory and small proportion of herbivorous species (e.g. Hymenoptera), and (d) a seemingly limited number of origins of herbivory and large proportion of herbivorous species (e.g. Hemiptera, Orthoptera).

For our purposes here, the most important question is whether clades with higher proportions of herbivorous species are diversifying faster than those with smaller proportions (indicating that herbivory is driving the rapid diversification). It seems unlikely that herbivory is driving rapid diversification across an entire clade if the proportion of herbivorous species in that clade is very low (i.e. a trait present in <10% of the species cannot be driving >50% of the speciation events). On the other hand, if two sister clades consist mostly of non-herbivorous species, and only one of them has one (or more) small subclades of rapidly diversifying herbivorous species, this should lead to a slightly higher proportion of herbivous species in that clade overall and a somewhat higher rate of net diversification (regardless of exactly how many origins of herbivory there were). We also note that the same issues and reasoning apply to our analyses of families and other subclades within insect orders (e.g. many beetle subclades have both herbivorous and non-herbivorous species: it should have no impact on our analyses if there was a single origin of herbivory within each subclade or if there were dozens).

Analyses of herbivory and diversification rates within orders

We also tested for a relationship between herbivory and diversification within most of the insect orders that are variable for the presence or absence of herbivory: Coleoptera, Diptera, Hemiptera, Hymenoptera, and Orthoptera. Note that Lepidoptera and Collembola are effectively invariant for the presence of herbivory, and we did not include Thysanoptera because the most recent phylogeny[4] included too few families (n = 4) to allow for a rigorous comparative analysis. Methods for estimating diversification rates and conducting PGLS regression generally followed those across orders, as described above.

For Coleoptera, we used the time-calibrated phylogeny of beetle families and subfamilies from Hunt and colleagues[5], from their Fig. 3. Hunt and colleagues[5] also included data on the species richness of these higher taxa, and data on feeding habits for almost all higher taxa in this tree (their Table S8). Again, we followed Hunt and colleagues[5] in only considering taxa that feed on living tracheophyte tissues to be herbivorous (excluding xylophagous species). For some taxa, they listed multiple feeding types as present within the clade, without quantifying specific proportions. In these cases, we arbitrarily assumed that different feeding types were at equal frequencies within each family (see above). However, we also performed an analysis in which clades were simply coded as herbivorous (state 1) or not (state 0), and any occurrence of herbivory in the clade was coded as herbivory present, even if other diets were also present. This approach also provided a direct parallel to the methodology used for Diptera, Hemiptera, Hymenoptera, and Orthoptera (see below). For six higher taxa, Hunt and colleagues[5]

listed the feeding habits as unknown. These taxa were excluded from the comparative analyses. Furthermore, Hunt and colleagues[5] included a few subfamilies that their tree suggested were not monophyletic. In these cases, they took the estimated number of described species in each subfamily and assigned equal numbers of species to each taxon. Here, we took a slightly different approach. Rather than using non-monophyletic subfamilies with uncertain ages and species numbers, we simply used the entire family as a terminal unit in these cases (combining the monophyletic and non-monophyletic subfamilies to estimate species numbers). We did this for Cryptophagidae (represented in our tree by Cryptophagiinae), Monotomidae (represented by Monotominae), Sphindidae (represented by *Sphindus*), Hydraenidae (represented by *Hydraena*). For Elateridae, we combined the two representatives of Denticollinae into one subfamily (represented by *Denticollis*). For Leiodidae, we combined the subfamilies Cholevinae and Leiodinae into one clade (represented by *Nargus*). The final analysis included 321 higher taxa of beetles. We then estimated diversification rates for each clade given the species numbers for each clade they provided and the stem ages from their tree, using the method-of-moment estimator from Magallón and Sanderson[63]. We tested for a relationship between herbivory and diversification rates using PGLS. The reduced tree used in these analyses is shown in nexus format in Supplementary Data 4 and the matching data on species richness, stem ages, diversification rates, and herbivory are shown in Supplementary Table 11. The results are shown in Supplementary Table 12.

In theory, we could have carried out the analyses of Coleoptera using the tree of Rainford and colleagues[4]. However, this would have required amalgamating higher taxa (Hunt and colleagues[5] generally used subfamilies, whereas Rainford and colleagues[4] used

families), and therefore dramatically reduced sample sizes and statistical power (n = 321 taxa using the tree of Hunt and colleagues[5]; n = 141 using the tree of Rainford and colleagues[4]). Furthermore, this amalgamation of subfamilies would have led to even less precise estimates of the proportion of herbivory in each taxon.

For Diptera we used the data on phylogeny, divergence times, species richness, and diets for dipteran families provided by Wiegmann and colleagues[6]. However, as mentioned above, some modifications were necessary (removing Bolbomyiidae and Mythiocomidae, using Cypselosomatidae, missing species numbers for Asteiidae, Chironomidae, Hilarimorphidae, and Xylophagidae). We followed Wiegmann and colleagues[6] and coded families as herbivorous if they were listed as herbivorous (phytophagous) in their Figure 1. We estimated diversification rates for each family using the method-of-moment estimator for stem ages[63], and then tested for a relationship between herbivory and diversification rates using PGLS. The tree used in these analyses is shown in nexus format in Supplementary Data 5 and the matching data on species richness, herbivory, stem ages, and diversification rates are shown in Supplementary Table 13. The results are shown in Supplementary Table 14.

Again, these analyses could have been repeated using the dipteran tree of Rainford and colleagues[4]. However, this would have reduced the included taxa from 142 to 118 (making it difficult to tell whether any differences in results were due to different trees or to reduced sample sizes instead).

For Hemiptera, we used the data on phylogeny, clade ages, and species richness for the 93 hemipteran families from Rainford and colleagues[4]. Since all but 5 of these families have either 0% or 100% herbivorous species (see above), we treated the presence

of herbivory in a family as a binary variable. The tree used in these analyses is shown in nexus format in Supplementary Data 6 and the matching data on species richness, herbivory, stem ages, and diversification rates are shown in Supplementary Table 15. The results are shown in Supplementary Table 16.

For Hymenoptera, we used the data on phylogeny, clade ages, and species richness for 77 families from Rainford and colleagues[4]. As described above, families were treated as herbivorous or not (binary variable), except for two families known to have an extremely low proportion of herbivorous species. The tree used in these analyses is shown in nexus format in Supplementary Data 7 and the matching data on species richness, herbivory, stem ages, and diversification rates are shown in Supplementary Table 17. The results are shown in Supplementary Table 18.

For Orthoptera, we used the phylogeny, clade ages, and species richness from Rainford and colleagues[4]. However, we excluded two families (Rhipipterygidae and Xyronotidae, see above) for which data on diet were unavailable and which were excluded entirely. A total of 26 families were included. As described above, families were treated as herbivorous or not (binary variable). The tree used in these analyses is shown in nexus format in Supplementary Data 8 and the matching data on species richness, herbivory, stem ages, and diversification rates are shown in Supplementary Table 19. The results are shown in Supplementary Table 20.

Supplementary References

1. Grimaldi, D. & Engel, M. S. *Evolution of the Insects*. (Cambridge University Press, 2005).
2. Hendrix, S. D. An evolutionary and ecological perspective of the insect fauna of ferns. *Am. Nat.* **115,** 171–196 (1980).
3. Misof, B. S. *et al.* Phylogenomics resolves the timing and pattern of insect evolution. *Science* **346,** 763–767 (2014).
4. Rainford, J. L., Hofreiter, M., Nicholson, D. B. & Mayhew, P. J. Phylogenetic distribution of extant richness suggests metamorphosis is a key innovation driving diversification in insects. *PLOS ONE* **9,** e109085 (2014).
5. Hunt, T. *et al.* A comprehensive phylogeny of beetles reveals the evolutionary origins of a superradiation. *Science*, **318,** 1913–1916 (2007).
6. Wiegmann, B. M. *et al.* Episodic radiations in the fly tree of life. *Proc. Natl. Acad. Sci. U.S.A.* **108,** 5690–5695 (2011).
7. Wiegmann B. M. *et al.* Single-copy nuclear genes resolve the phylogeny of the holometabolous insects. *BMC Biol* **7,** 34 (2009).
8. McKenna, D. D. & Farrell, B. D. 9-Genes reinforce the phylogeny of Holometabola and yield alternate views on the phylogenetic placement of Strepsiptera. *PLoS ONE* **5,** e11887 (2010).
9. Rota-Stabelli, O., Daley, A. C. & Pisani, D. Molecular timetrees reveal a Cambrian colonization of land and a new scenario for ecdysozoan evolution. *Curr. Biol.* **23,** 392–398 (2013).
10. Thomas, J. A., Trueman, J. W. H., Rambaut, A. & Welch, J. J. Relaxed

phylogenetics and the Palaeoptera problem: resolving deep ancestral splits in the insect phylogeny. *Syst. Biol.* **62,** 285–297 (2013).

11. Wheat, C. W. & Wahlberg, N. Phylogenomic insights into the Cambrian Explosion, the colonization of land and the evolution of flight in Arthropoda. *Syst. Biol*. **62,** 93–109 (2013).
12. Ishiwata, K. Sasaki, G., Ogawa, J., Miyata, T. & Su Z.-H. Phylogenetic relationships among insect orders based on three nuclear protein-coding gene sequences. *Mol. Phylogenet. Evol.* **58,** 169–180 (2011).
13. Sasaki, G., Ishiwata, K., Machida, R., Miyata, T. & Su, Z.-H. Molecular phylogenetic analyses support the monophyly of Hexapoda and suggest the paraphyly of Entognatha. *BMC Evol. Biol.* **13,** 236 (2013).
14. Wiens, J. J. & Morrill, M. C. Missing data in phylogenetic analysis: reconciling results from simulations and empirical data. *Syst. Biol.* **60,** 719–731 (2011).
15. Wiens, J. J. & Tiu, J. Highly incomplete taxa can rescue phylogenetic analyses from the negative impacts of limited taxon sampling. *PLoS ONE* **7,** e42925 (2012).
16. Jiang, W., Chen, S.-Y., Wang, H. Li , D.-Z. & Wiens, J. J. Should genes with missing data be excluded from phylogenetic analyses? *Mol. Phylogenet. Evol.* **80,** 308–318 (2014).
17. Zheng, Y. & Wiens, J. J. Do missing data influence the accuracy of divergence-time estimation with BEAST? *Mol. Phylogenet. Evol.* **85,** 41–49 (2015).
18. Edgar, R. C. MUSCLE: multiple sequence alignment with high accuracy and high throughput. *Nucleic Acids Res*. **32,** 1792–1797 (2004).

19. Drummond, A. *et al.* Geneious v6.1.7. Available online at: http://www.geneious (2012).

20. Talavera, G., Castresana, J. Improvement of phylogenies after removing divergent and ambiguously aligned blocks from protein sequence alignments. *Syst. Biol.* **56,** 564–577 (2007).

21. Lanfear, R., Calcott, B., Kainer, D., Mayer, C. & Stamatakis, A. Selecting optimal partitioning schemes for phylogenomic datasets. *BMC Evol. Biol.* **14,** 82 (2014).

22. Stamatakis, A. RAxML Version 8: A tool for phylogenetic analysis and post-analysis of large phylogenies. *Bioinformatics* **30,** 1312–1313 (2014).

23. Trautwein, M. D., Wiegmann, B., Beutel, R., Kjer, K. &. Yeates, D. K. Advances in insect phylogeny at the dawn of the postgenomic era. *Ann. Rev. Entomol.* **57,** 449–468 (2012).

24. Bouckaert, R. *et al.* BEAST 2: A software platform for Bayesian evolutionary analysis. *PLoS Comput. Biol.* **10,** e1003537 (2014).

25. Rambaut, A., Suchard, M. A., Xie, D. & Drummond, A. J. Tracer v1.6, 2014. URL http://beast.bio.ed.ac.uk/Tracer (2014).

26. Ayres, D. L. *et al.* BEAGLE: an application programming interface and high-performance computing library for statistical phylogenetics. *Syst. Biol.* **61,** 170–173 (2012).

27. Benton, M., Donoghue, P. C. J. & Asher, R. J. In *The Timetree of Life*, S.B. Hedges, Kumar, S. 35–86. Oxford University Press (2009).

28. Gradstein, F. M., Ogg, J. G., Schmitz, M. D. & Ogg, G. M. *The Geologic Time Scale 2012*. (Elsevier, 2012).

29. Vršansk., P. Cretaceous Gondwanian cockroaches (Insecta: Blattaria). *Entomol. Prob.* **34,** 49–54 (2004).

30. Vršansk, P. & Ansorge, J. Lower Jurassic cockroaches (Insecta: Blattaria) from Germany and England. *African Invert.* **48**, 103–126 (2007).

31. Wilson, H. M. & Martill, D. M. A new japygid dipluran from the Lower Cretaceous of Brazil. *Palaeontol.* **44,** 1025–1031 (2001).

32. Labandeira, C. C., Dilcher, D. L., Davis, D. R. & Wagner, D. L. Ninety-seven million years of angiosperm-insect association: paleobiological insights into the meaning of coevolution. *Proc. Natl. Acad. Sci. U.S.A.* **91,** 12278–12282 (1994).

33. Zhang, W. *et al.* New fossil Lepidoptera (Insecta: Amphiesmenoptera) from the Middle Jurassic Jiulongshan Formation of Northeastern China. *PLoS ONE* **8,** e79500 (2013).

34. Whalley, P. E. The systematics and palaeogeography of the Lower Jurassic insects of Dorset, England. *Bull. Brit. Mus. (Nat. Hist.) Geol. Ser.* **39,** 107–189 (1985).

35. Ponomarenko, A. G. Historical development of Archostematan beetles. *Tr. Paleontol. Inst. Akad. Nauk.* 125, 1–240 (1969).

36. Béthoux, O., Nel, A., Gand, G., Lapeyrie, J. & Galtier, J. Discovery of the genus *Iasvia* Zalessky, 1934 in the Upper Permian of France (Lodève basin) (Orthoptera, Ensifera, Oedischiidae). *Geobios* **35,** 293–302 (2002).

37. Grimaldi, D. & Engel, M. S. Fossil Liposcelididae and the lice ages (Insecta: Psocodea). *Proc. R. Soc. Lond. B* **273,** 625–633 (2006).

38. Engel, M. S. & Grimaldi, D. A. New light shed on the oldest insect. *Nature* **427,** 627–630 (2004).

39. Engel, M. S., Grimaldi, D. A. & Krishna, K. Primitive termites from the early Cretaceous of Asia. *Stuttgarter Beiträge zur Naturkunde Serie B (Geologie und Paläontologie)* **371,** 32 (2007).

40. Zhang, J.-F. & Kluge, N. J. Jurassic larvae of mayflies (Ephemeroptera) from the Daohugou Formation in Inner Mongolia, China. *Orient. Insects* **41,** 351–366 (2007).

41. Zhang, H., Wang, M. & Liu, X. Constraints on the upper boundary age of the Tiaojishan Formation volcanic rocks in West Liaoning-North Hebei by LA-ICP-MS dating. *Chinese Science Bulletin* **53**, 3574–3584 (2008).

42. Lacasa-Ruiz, A. & Martínez-Delclòs, X. *Meiaterme*s: nuevo género fósil de insecto isóptero (Hodotermitidae) de las calizas Necomienses del Montsec (Provincia de Lérida, España). *Lleida Institut d'Estudis Ilerdencs*, 65 pp. (1986).

43. Mitter, C., Farrell, B. & Wiegmann, B. The phylogenetic study of adaptive zones: Has phytophagy promoted insect diversification? *Am. Nat.***132,** 107–28 (1988).

44. Frost, S. W. The numerical relationships between phytophagous insects and their hosts. *Sci. Mon.* **79,** 10–12 (1954).

45. Courtney, G. W., Pape, T., Skevington, J. H. & Sinclair, B. J. In *Insect Biodiversity: Science and Society* (R. G. Foottit R. G., Adler, P. H. 185–222. John Wiley and Sons (2009).

46. Webb, D. W. Hilarimorphidae. *Manual of Nearctic Diptera* **1,** 603–605 (1981).

47. Strong, D. R., Lawton, J. H. & Southwood, T. R. E. *Insects on Plants: Community Patterns and Mechanisms*. (Harvard University Press, 1984).

48. Schuh, R. T. & Slater, J. A. *True Bugs of the World (Hemiptera: Heteroptera): Classification and Natural History.* (Cornell University Press, 1995).

49. Myers, P. *et al.* The Animal Diversity Web (online). Accessed at http://animaldiversity.org (2015).

50. Ashlock, P. D. & Gagné, W.C. A remarkable new micropterous *Nysius* species from the aeolian zone of Mauna Kea, Hawai‘i island (Hemiptera: Heteroptera: Lygaeidae). *Int. J. Ent.* **25,** 47–55 (1983).

51. Malipatil, M. B. Revision of world Cleradini (Heteroptera: Lygaeidae), with a cladistic analysis of relationships within the tribe. *Aust. J. Zool*. **31,** 205–225 (1983).

52. Goulet, H. & Huber, J. (eds.). *Hymenoptera of the World: An Identification Guide to Families*. Research Branch Agriculture Canada Publication l894/E (1993).

53. de Macêdo, M. V., & Monteiro, R. T. Seed predation by a braconid wasp, *Allorhogas* sp. (Hymenoptera). *J. New York Ent. Soc.* **97,** 359–362 (1989).

54. Marsh, P. M. Description of a phytophagous doryctinae braconid from Brazil (Hymenoptera: Braconidae). *Proc. Ent. Soc. Wash*. **93,** 92–95 (1991).

55. Infante, F., Hanson, P. & Wharton, R. Phytophagy in the genus *Monitoriella* (Hymenoptera: Braconidae) with description of new species. *Ann. Ent. Soc. Am.* **88,** 406–415 (1995).

56. Pierce, N. E. Predatory and parasitic Lepidoptera: carnivores living on plants. *J. Lep. Soc.* 49, 412–453 (1995).

57. Preston-Mafham, K. *Grasshoppers and Mantids of the World*. (Blanford, 1991).

58. Capinera, J. L. In *Encyclopedia of Entomology, Second Edition* Capinera, J. L. Springer (2008).

59. Bedford, G. O. Biology and ecology of the Phasmatodea. *Ann. Rev. Entomol.* **23,** 125–149 (1978).

60. ThripsWiki. *ThripsWiki - providing information on the World's thrips*. <http://thrips.info/wiki/Main_Page> [November 30, 2014]

61. Watson, D. M. *et al.* The effect of two prey species, *Chrysomphalus aonidum* and *Corcyra cephalonica*, on the quality of the predatory thrips, *Aleurodothrips fasciapennis*, reared in the laboratory. *BioControl* **45,** 45–61 (2000).

62. Childers, C. C. & Nakahara, S. Thysanoptera (Thrips) within citrus orchards in Florida: species distribution, relative and seasonal abundance within trees, and species on vines and ground cover plants. *J. Insect Sci.* **6,** 1–19 (2006).

63. Magallón, S. & Sanderson, M. J. Absolute diversification rates in angiosperm clades. *Evolution* **55,** 1762–1780 (2001).
