# Supplementary material for: Herbivory increases diversification across insect clades
Source: Nat Commun. 2015 Sep 24;6:8370. doi: 10.1038/ncomms9370 (PMC4598556; doi:10.1038/ncomms9370)
Supplement: Supplementary Data 1 — Phylogeny of insect orders based on figure 1 of Misof and colleagues11 [file ncomms9370-s2.docx]

**Supplementary Data 1. Phylogeny of insect orders based on figure 1 of Misof and colleagues^11^**

#NEXUS

begin taxa;

dimensions ntax=31;

taxlabels

Protura

Collembola

Diplura

Archaeognatha

Zygentoma

Ephemeroptera

Odonata

Thysanoptera

Hemiptera

Psocodea

Hymenoptera

Coleoptera

Strepsiptera

Neuroptera

Megaloptera

Raphidioptera

Diptera

Siphonaptera

Mecoptera

Lepidoptera

Trichoptera

Dermaptera

Zoroptera

Plecoptera

Mantodea

Blattodea

Embioptera

Phasmatodea

Mantophasmatodea

Grylloblattodea

Orthoptera

;

end;

begin trees;

tree PAUP_1 = [&R] ((Protura:430.06533,Collembola:430.06533):49.059373,(Diplura:461.580256,(Archaeognatha:440.33867,(Zygentoma:420.54906,((Ephemeroptera:362.454433,Odonata:362.454433):43.996795,(((Thysanoptera:339.127642,Hemiptera:339.127642):34.15908,(Psocodea:361.532244,(Hymenoptera:344.679769,(((Coleoptera:286.472908,Strepsiptera:286.472908):21.235664,((Neuroptera:259.034097,Megaloptera:259.034097):17.223379,Raphidioptera:276.257476):31.451096):18.983028,(((Diptera:143.790571,Siphonaptera:143.790571):98.952262,Mecoptera:242.742833):46.906675,(Lepidoptera:207.200741,Trichoptera:207.200741):82.448767):37.042092):17.988169):16.852475):11.754479):13.602412,((Dermaptera:168.523376,Zoroptera:168.523376):133.52205,(Plecoptera:269.115057,(((Mantodea:197.319693,Blattodea:197.319693):33.951518,((Embioptera:164.221258,Phasmatodea:164.221258):39.514701,(Mantophasmatodea:152.669282,Grylloblattodea:152.669282):51.066677):27.535252):16.573367,Orthoptera:247.844578):21.270479):32.930368):84.843708):19.562094):14.097832):19.78961):21.241586):17.544447);

end;
