# Supplementary material for: Herbivory increases diversification across insect clades
Source: Nat Commun. 2015 Sep 24;6:8370. doi: 10.1038/ncomms9370 (PMC4598556; doi:10.1038/ncomms9370)
Supplement: Supplementary Data 2 — Phylogeny of insect orders based on the study of Rainford and colleagues12 [file ncomms9370-s3.docx]

**Supplementary Data 2. Phylogeny of insect orders based on the study of Rainford and colleagues^12^**

#NEXUS

begin taxa;

dimensions ntax=31;

taxlabels

Dipl_Campo [Diplura]

Pro_Fujien [Protura]

Coll_Neeli [Collembola]

Archaeogna [Archaeognatha]

Zy_Lepidot [Zygentoma]

Od_A_Petal [Odonata]

Ep_Leptoph [Ephemeroptera]

Der_Apachy [Dermaptera]

Pl_Perlida [Plecoptera]

Or_E_Grylli [Orthoptera]

Grylloblat [Grylloblattodea]

Mantophasm [Mantophasmatodea]

Emb_Terate [Embioptera]

Pha_Timema [Phasmatodea]

Zoraptera [Zoraptera]

Bla_Blatti [Blattodea]

Man_Acanth [Mantodea]

Ps_Pachytr [Psocodea]

Thy_Hetero [Thysanoptera]

He_Psylloi [Hemiptera]

Hy_Xyelida [Hymenoptera]

Rap_Raphid [Rhaphidioptera]

Meg_Sialid [Megaloptera]

Neu_Osmyli [Neuroptera]

Strepsipte [Strepsiptera]

Co_Ar_Cupe [Coleoptera]

Mec_Nannoc [Mecoptera]

Si_Ctenoph [Siphanoptera]

Di_Deutero [Diptera]

Tr_Hydropt [Trichoptera]

L_Agathiph [Lepidoptera]

;

end;

begin trees;

tree PAUP_1 = [&R] (((Dipl_Campo:440.9346,Pro_Fujien:440.93451):23.05306,Coll_Neeli:463.9876):14.10081,(Archaeogna:462.3179,(Zy_Lepidot:449.197,((Od_A_Petal:373.26056,Ep_Leptoph:373.26053):68.34376,(((Der_Apachy:356.6553,Pl_Perlida:356.6552):44.57514,(Or_E_Grylli:387.34721,(((Grylloblat:220.4914,Mantophasm:220.4914):86.67753,(Emb_Terate:259.64834,Pha_Timema:259.64836):47.52058):55.64524,(Zoraptera:340.5617,(Bla_Blatti:307.23499,Man_Acanth:307.23498):33.32666):22.25249):24.53302):13.88326):29.61081,((Ps_Pachytr:416.60237,(Thy_Hetero:404.61123,He_Psylloi:404.61117):11.99119):10.19092,(Hy_Xyelida:389.6906,(((Rap_Raphid:300.3542,(Meg_Sialid:284.83226,Neu_Osmyli:284.83235):15.52195):33.81282,(Strepsipte:309.3164,Co_Ar_Cupe:309.316449):24.85062):27.47581,(((Mec_Nannoc:260.77357,Si_Ctenoph:260.77356):56.04382,Di_Deutero:316.81734):4.03331,(Tr_Hydropt:302.41197,L_Agathiph:302.41198):18.43866):40.79222):28.04764):37.10277):4.047947):10.76307):7.59272):13.12091):15.77048);

end;
