# Supplementary material for: Herbivory increases diversification across insect clades
Source: Nat Commun. 2015 Sep 24;6:8370. doi: 10.1038/ncomms9370 (PMC4598556; doi:10.1038/ncomms9370)
Supplement: Supplementary Data 3 — Time-calibrated phylogeny of insect orders based on the results of this study [file ncomms9370-s4.docx]

**Supplementary Data 3. Time-calibrated phylogeny of insect orders based on the results of this study**

#NEXUS

begin taxa;

dimensions ntax=31;

taxlabels

Collembola

Diplura

Protura

Diptera

Mecoptera

Siphonaptera

Trichoptera

Lepidoptera

Strepsiptera

Coleoptera

Raphidioptera

Neuroptera

Megaloptera

Hymenoptera

Hemiptera

Thysanoptera

Psocodea

Plecoptera

Dermaptera

Embioptera

Phasmatodea

Grylloblattodea

Mantophasmatodea

Orthoptera

Blattodea

Mantodea

Zoroptera

Odonata

Ephemeroptera

Zygentoma

Archaeognatha

;

end;

begin trees;

tree PAUP_1 = [&R] ((Collembola:539.413568,(Diplura:473.917943,Protura:473.917943):65.495625):49.790582,(((((((((Diptera:278.546497,(Mecoptera:223.092964,Siphonaptera:223.092964):55.453532):36.72959,(Trichoptera:193.428947,Lepidoptera:193.428947):121.84714):43.590449,((Strepsiptera:309.115984,Coleoptera:309.115985):22.18426,((Raphidioptera:192.082755,Neuroptera:192.082755):53.344835,Megaloptera:245.42759):85.872655):27.566291):16.573495,Hymenoptera:375.440031):16.20158,((Hemiptera:255.770482,Thysanoptera:255.770482):70.882969,Psocodea:326.653451):64.988159):10.710275,((Plecoptera:255.200853,Dermaptera:255.200852):81.403466,(((Embioptera:200.988774,Phasmatodea:200.988774):85.286025,(Grylloblattodea:178.528192,Mantophasmatodea:178.528192):107.746607):23.552052,(Orthoptera:293.601549,((Blattodea:210.375344,Mantodea:210.375344):50.500813,Zoroptera:260.876157):32.725392):16.225303):26.777468):65.747567):24.191151,(Odonata:344.133492,Ephemeroptera:344.133492):82.409545):29.82589,Zygentoma:456.368927):75.9245,Archaeognatha:532.293427):56.910723);

end;
