# Supplementary material for: Herbivory increases diversification across insect clades
Source: Nat Commun. 2015 Sep 24;6:8370. doi: 10.1038/ncomms9370 (PMC4598556; doi:10.1038/ncomms9370)
Supplement: Supplementary Data 4 — Reduced phylogeny of beetle subfamilies and families used in this study as modified from Hunt and colleagues10 [file ncomms9370-s5.docx]

**Supplementary Data 4. Reduced phylogeny of beetle subfamilies and families used in this study as modified from Hunt and colleagues^10^**

#NEXUS

begin taxa;

dimensions ntax=321;

taxlabels

CPCTeAn_Anthicinae

CPCTeMe_Lyttinae

CPCTeMe_Meloinae

CPCTeMe_Nemognathinae

CPCTeAn_Eurygeniinae

CPCTeCi_Ciinae

CPCTeTe_Phrenapatinae

CPCTeTe_Diaperinae

CPCTeTe_Alleculinae

CPCTeTe_Coelometopinae

CPCTeTe_Tenebrioninae

CPCTeZo_Usechinae

CPCTeZo_Zopherinae

CPCTePy_Agnathinae

CPCTe_Monommatidae

CPCTeTe_Pimeliinae

CPCTeSa_Prostominiinae

CPCTeSa_Trogocryptinae

CPCTeMy_Mycetophaginae

CPCTeMea_Eustrophinae

CPCTeSt_Cephaloinae

CPCTeZo_Colydiinae

CPCTeSa_Othniinae

CPCTe_Boridae

CPCTeOe_Oedemerinae

CPCTeSc_Anaspidinae

CPCTeMea_Osphyinae

CPCTeSc_Scraptiinae

CPCTeMea_Melandryinae

CPCTeMea_Hypulinae

CPCTeTer_Penthinae

CPCTe_Trictenotomidae

CPCTe_Pythidae

CPCTePy_Pedilinae

CPCTePy_Pyrochroinae

CPCTeSa_Inopeplinae

CPCTeSa_Aegialitinae

CPCTeSa_Salpinginae

CPCTeMea_Hallomeninae

CPCTeTer_Tetratominae

CPCTe_Perimylopidae

CPCTeAn_Ischaliinae

CPCTe_Aderidae

CPCTeTe_Lagriinae

CPCTeRi_Pelecotominae

CPCTeRi_Rhipidiinae

CPCTeRi_Rhipiphorinae

CPCLyLy_Hylecoetinae

CPCLyLy_Melittommatinae

CPCTeMo_Mordellinae

CPCLyLy_Lymexylinae

CPCCuSpSp_Sphindus

CPCClCl_Clerinae

CPCClCl_Hydnocerinae

CPCClCl_Korynetinae

CPCClCl_Enopliinae

CPCClCl_Tillinae

CPCClMe_Prionocerinae

CPCClMe_Malachiinae

CPCClMe_Dasytinae

CPCClMe_Melyrinae

CPCClMe_Rhadalinae

CPCClTr_Peltinae

CPCClTr_Lophocaterinae

CPCCu_Byturidae

CPCCu_Biphyllidae

CPCClPh_Phloiophilidae

CPCClTr_Trogossitinae

CPCCuCo_Scymninae

CPCCuCo_Chilocorinae

CPCCuCo_Coccidulinae

CPCCuCo_Epilachninae

CPCCuCo_Coccinellinae

CPCCu_Alexiidae

CPCCuEn_Anamorphinae

CPCCuEn_Leiestinae

CPCCuEn_Holoparamecinae

CPCCuCoy_Corylophinae

CPCCuCoy_Sericoderinae

CPCCuEn_Endomychinae

CPCCuEn_Lycoperdininae

CPCCuLah_Corticariinae

CPCCuLah_Latridiinae

CPCCu_Discolomatidae

CPCCuCe_Euxestinae

CPCCuBo_Teredinae

CPCCuBo_Anommatinae

CPCCuBo_Xylariophilinae

CPCCuCe_Ceryloninae

CPCCuPha_Phalacrinae

CPCCu_Laemophloeidae

CPCCu_Propalticidae

CPCCuCr_Cryptophaginae

CPCCu_Passandridae

CPCCu_Cucujidae

CPCCuEr_Tritominae

CPCCuLag_Xenoscelinae

CPCCuLag_Languriinae

CPCCuEr_Megalodacninae

CPCCuEr_Encaustinae

CPCCuEr_Dacninae

CPCCuLag_Toraminae

CPCCuLag_Cryptophilinae

CPCCuEr_Erotylinae

CPCCu_Protocucujidae

CPCCuMo_Monotominae

CPCCuNi_Carpophilinae

CPCCuNi_Cryptarchinae

CPCCuNi_Cillaeinae

CPCCuNi_Nitidulinae

CPCChCh_Chrysomelinae

CPCChCh_Galerucinae

CPCChCh_Lamprosmatinae

CPCChCh_Eumolpinae

CPCChCh_Cryptocephalinae

CPCChCh_Donaciinae

CPCChCh_Criocerinae

CPCChCh_Bruchinae

CPCChCh_Sagrinae

CPCChCe_Vesperinae

CPCChOr_Aulacoscelidinae

CPCChMe_Palophaginae

CPCChMe_Zeugophorinae

CPCChCe_Parandrinae

CPCChCe_Spondylidinae

CPCChOr_Orsodacninae

CPCChCe_Disteniinae

CPCChCe_Necydalinae

CPCChCe_Lepturinae

CPCChCe_Lamiinae

CPCChCe_Prioninae

CPCChCe_Cerambycinae

CPCCucNe_Rhinorhynchinae

CPCCucAn_Choraginae

CPCCucAn_Anthribinae

CPCCucAn_Urodontinae

CPCCucAt_Rhynchitinae

CPCCucAt_Attelabinae

CPCCucNe_Doydirhynchinae

CPCCucBr_Cycladinae

CPCCucCu_Scolytinae

CPCCucBr_Brentinae

CPCCucCu_Cossoninae

CPCCucCu_Platypodinae

CPCCucCu_Curculioninae

CPCCucCu_Dryophthorinae

CPCCuc_Caridae

CPCCucCu_Brachycerinae

CPCCucIt_Ithyceridae

CPCCucBr_Nanophyinae

CPCCucBr_Apioninae

CPCCucBe_Belinae

CPCCucBe_Oxycoryninae

CPCChCh_Hispinae

CPCChCh_Cassidinae

CPCCuSi_Silvaninae

CPCCuSi_Brontinae

CPEBy_Callirhipidae

CPEByPt_Ptilodactylinae

CPEByPt_Cladotominae

CPEByEl_Larainae

CPEBy_Chelonariidae

CPEByEl_Elminae

CPEBy_Eulichadidae

CPEByPs_Eubrianacinae

CPEByLi_Limnichinae

CPEBy_Dryopidae

CPEElLa_Luciolinae

CPEElLa_Ototretinae

CPEElLy_Erotinae

CPEElLy_Lycinae

CPEElLy_Calochrominae

CPEElLy_Metriorrhynchinae

CPEElLy_Ateliinae

CPEElLy_Leptolycinae

CPEElCa_Cantharinae

CPEElCa_Chauliognathinae

CPEElCa_Malthininae

CPEElEl_Cardiophorinae

CPEEl_Phengodidae

CPEElEl_Elaterinae

CPEElEl_Thylacosterninae

CPEEl_Rhagophthalmidae

CPEElElDe_Denticollis

CPEEl_Drilidae

CPEElEl_Agrypninae

CPEEl_Omalisidae

CPEElEu_Eucneminae

CPEEl_Throscidae

CPEDaDa_Dascillinae

CPEDa_Rhipiceridae

CPEByBy_Byrrhinae

CPEByBy_Syncalyptinae

CPBBo_Nosodendridae

CPEBuBu_Agrilinae

CPEBuBu_Buprestinae

CPEBuBu_Acmaeoderinae

CPEBuBu_Julodinae

CPEBuBu_Trachyinae

CPEBy_Heterocerinae

CPBBoAn_Dryophilinae

CPBBoBo_Bostrichinae

CPBBoBo_Dinoderinae

CPBBoBo_Lyctinae

CPBBoAn_Mesocoelopodinae

CPBBoAn_Ptilininae

CPBBoAn_Dorcatominae

CPBBoAn_Xyletininae

CPBBoAn_Anobiinae

CPBBoAn_Gibbiinae

CPBBoAn_Ptininae

CPBBoDe_Dermestinae

CPBBoDe_Trinodinae

CPBBoDe_Megatominae

CPBBoDe_Attageninae

CPBBoDe_Orphilinae

CPScScSc_Dynastinae

CPScScSc_Rutelinae

CPScScSc_Orphninae

CPScScSc_Melolonthinae

CPScScSc_Cetoniinae

CPScSc_Glaphyridae

CPScScOc_Ochodaeinae

CPScScSc_Scarabaeinae

CPScSc_Glaresidae

CPScSc_Hybosoridae

CPScScSc_Aphodiinae

CPScScLu_Dorcinae

CPScScLu_Lucaninae

CPScScLu_Aesalinae

CPScScLu_Nicaginae

CPScSc_Ceratocanthidae

CPScScGe_Bolboceratinae

CPScSc_Trogidae

CPScScPa_Aulacocyclinae

CPScScPa_Passalinae

CPSHyHy_Hydrophilinae

CPSHy_Hydrochidae

CPSHy_Spercheidae

CPSHy_Epimetopidae

CPSHy_Georissidae

CPSHyHy_Sphaeridiinae

CPSHy_Helophoridae

CPSStSt_Staphylininae

CPSStSt_Phloeocharinae

CPSStSt_Pseudopsinae

CPSStSt_Osoriinae

CPSStSt_Oxytelinae

CPSStSt_Paederinae

CPSStSt_Piestinae

CPSStSt_Euaesthetinae

CPSStSt_Oxyporinae

CPSStSt_Steninae

CPSStSt_Tachyporinae

CPSStSi_Silphinae

CPSStSt_Habrocerinae

CPSStSt_Micropeplinae

CPSStSt_Megalopsidiinae

CPSStSt_Aleocharinae

CPSStSt_Proteininae

CPSStSt_Omalinae

CPSStSt_Glypholomatinae

CPSStSt_Scaphidiinae

CPSStLe_Leiodinae

CPSStLeCh_Nargus

CPSStLe_Camiarinae

CPSSt_Agyrtidae

CPSStPt_Ptilinae

CPSStPt_Acrotrichinae

CPSStPt_Cephaloplectinae

CPSStHyHy_Hydraena

CPSHi_Sphaeritidae

CPSHiHi_Saprininae

CPSHiHi_Onthophilinae

CPSHiHi_Abraeinae

CPSHiHi_Dendrophilinae

CPSHiHi_Hetaeriinae

CPSHiHi_Tribalinae

CPSHiHi_Histerinae

CPSHi_Synteliidae

CPSStSc_Scydmaeninae

CPSStSc_Mastiginae

CPEScCl_Clambinae

CPESc_Eucinetidae

CPBDeDe_Laricobiinae

CPEScSc_Scirtidae

CAC_Elaphrinae

CAC_Brachininae

CAC_Siagoninae

CA_Trachypachidae

CAC_Rhysodinae

CAC_Loricerinae

CAC_Migadopinae

CAC_Cicindelinae

CAC_Paussinae

CAC_Harpalinae

CAC_Carabinae

CAC_Trechinae

CAC_Gehringiinae

CAC_Scaritinae

CAC_Omophroninae

CAG_Sphanglerogyrinae

CAG_Gyrininae

CA_Haliplidae

CAN_Notomicrinae

CAN_Noterinae

CAD_Dytiscinae

CAD_Laccophilinae

CAD_Copelatinae

CA_Amphizoidae

CAD_Hydroporinae

CAD_Agabinae

CAD_Colymbetinae

CAD_Coptotominae

CAD_Lancetinae

CA_Paelobiidae

CA_Aspidytidae

CM_Torridincolidae

CM_Hydroscaphidae

CAr_Cupedidae

CM_Sphaeriusidae

;

end;

begin trees;

tree PAUP_1 = [&R] ((((((((((((((((((((CPCTeAn_Anthicinae:130.713201,((CPCTeMe_Lyttinae:64.38836,CPCTeMe_Meloinae:64.38836):34.579806,CPCTeMe_Nemognathinae:98.968166):31.745035):21.930423,CPCTeAn_Eurygeniinae:152.643624):16.708885,CPCTeCi_Ciinae:169.352509):25.218337,((((CPCTeTe_Phrenapatinae:80.051546,CPCTeTe_Diaperinae:80.051546):42.302912,(CPCTeTe_Alleculinae:99.872157,CPCTeTe_Coelometopinae:99.872157):22.4823):24.237037,CPCTeTe_Tenebrioninae:146.591494):44.129242,(((((((CPCTeZo_Usechinae:14.233277,CPCTeZo_Zopherinae:14.233277):18.625234,(CPCTePy_Agnathinae:21.800674,CPCTe_Monommatidae:21.800674):11.057837):47.209301,CPCTeTe_Pimeliinae:80.067813,((CPCTeSa_Prostominiinae:13.04013,CPCTeSa_Trogocryptinae:13.04013):31.297408,CPCTeMy_Mycetophaginae:44.337538):35.730274):25.811494,(CPCTeMea_Eustrophinae:82.38896,CPCTeSt_Cephaloinae:82.38896):23.490347):27.520711,(CPCTeZo_Colydiinae:104.275774,CPCTeSa_Othniinae:104.275774):29.124244):28.265276,((((CPCTe_Boridae:108.209141,CPCTeOe_Oedemerinae:108.209141):38.170045,((CPCTeSc_Anaspidinae:110.481955,CPCTeMea_Osphyinae:110.481955):21.34041,CPCTeSc_Scraptiinae:131.822365):14.556822):6.986961,((((CPCTeMea_Melandryinae:87.906278,CPCTeMea_Hypulinae:87.906278):15.157787,CPCTeTer_Penthinae:103.064065):23.006812,CPCTe_Trictenotomidae:126.070877):16.425241,((CPCTe_Pythidae:105.670971,(CPCTePy_Pedilinae:78.157309,CPCTePy_Pyrochroinae:78.157309):27.513662):21.936599,(CPCTeSa_Inopeplinae:117.886719,(CPCTeSa_Aegialitinae:91.100362,CPCTeSa_Salpinginae:91.100362):26.786357):9.72085):14.888549):10.870029):5.700661,(CPCTeMea_Hallomeninae:135.482767,(CPCTeTer_Tetratominae:107.043277,CPCTe_Perimylopidae:107.043277):28.43949):23.584042):2.598485):17.652887,((CPCTeAn_Ischaliinae:118.291151,CPCTe_Aderidae:118.291151):27.540715,CPCTeTe_Lagriinae:145.831867):33.486314):11.402555):3.850111):4.581185,(((CPCTeRi_Pelecotominae:146.710561,CPCTeRi_Rhipidiinae:146.710561):22.921098,CPCTeRi_Rhipiphorinae:169.631659):11.879963,((CPCLyLy_Hylecoetinae:85.119723,CPCLyLy_Melittommatinae:85.119723):55.423675,CPCTeMo_Mordellinae:140.543398):40.968223):17.640409):7.313873,CPCLyLy_Lymexylinae:206.465904):4.246155,CPCCuSpSp_Sphindus:210.712059):12.065438,((((((((CPCClCl_Clerinae:77.483528,CPCClCl_Hydnocerinae:77.483528):36.674422,(CPCClCl_Korynetinae:93.376278,CPCClCl_Enopliinae:93.376278):20.781673):11.256294,CPCClCl_Tillinae:125.414244):26.607486,(((CPCClMe_Prionocerinae:102.75814,((CPCClMe_Malachiinae:48.94591,CPCClMe_Dasytinae:48.94591):32.044599,CPCClMe_Melyrinae:80.990509):21.767631):17.603482,CPCClMe_Rhadalinae:120.361622):20.138155,CPCClTr_Peltinae:140.499777):11.521953):9.964614,CPCClTr_Lophocaterinae:161.986344):17.717791,((CPCCu_Byturidae:54.505008,CPCCu_Biphyllidae:54.505008):81.561567,CPCClPh_Phloiophilidae:136.066575):43.63756):10.665457,CPCClTr_Trogossitinae:190.369593):20.603954,(((((((CPCCuCo_Scymninae:81.160014,CPCCuCo_Chilocorinae:81.160014):19.91355,CPCCuCo_Coccidulinae:101.073564):12.935458,CPCCuCo_Epilachninae:114.009022):17.493876,CPCCuCo_Coccinellinae:131.502898):26.669294,CPCCu_Alexiidae:158.172193,CPCCuEn_Anamorphinae:158.172193):22.172638,((CPCCuEn_Leiestinae:139.089829,(CPCCuEn_Holoparamecinae:120.496413,(CPCCuCoy_Corylophinae:66.838095,CPCCuCoy_Sericoderinae:66.838095):53.658319):18.593416):31.255806,((CPCCuEn_Endomychinae:58.450079,CPCCuEn_Lycoperdininae:58.450079):85.87396,(CPCCuLah_Corticariinae:110.63905,CPCCuLah_Latridiinae:110.63905):33.684989):26.021596):9.999195):10.572581,(((CPCCu_Discolomatidae:98.806788,(CPCCuCe_Euxestinae:59.290487,CPCCuBo_Teredinae:59.290487):39.516301,CPCCuBo_Anommatinae:98.806788):41.956115,CPCCuBo_Xylariophilinae:140.762903):16.763337,CPCCuCe_Ceryloninae:157.52624):33.391172):20.056135):11.803951):3.59203,(((((((CPCCuPha_Phalacrinae:107.263153,(CPCCu_Laemophloeidae:34.532904,CPCCu_Propalticidae:34.532904):72.730248):54.147811,CPCCuCr_Cryptophaginae:161.410964):12.337321,(CPCCu_Passandridae:75.261785,CPCCu_Cucujidae:75.261785):98.4865):16.40472,((((((CPCCuEr_Tritominae:68.38799,(CPCCuLag_Xenoscelinae:40.31217,CPCCuLag_Languriinae:40.31217):28.07582,CPCCuEr_Megalodacninae:68.38799):55.136254,(((CPCCuEr_Encaustinae:73.28344,CPCCuEr_Dacninae:73.28344):20.901554,CPCCuLag_Toraminae:94.184993):9.05464,CPCCuLag_Cryptophilinae:103.239633):20.284611):9.36092,CPCCuEr_Erotylinae:132.885164):20.630187,CPCCu_Protocucujidae:153.515351):12.85591,CPCCuMo_Monotominae:166.371261):17.846856,(CPCCuNi_Carpophilinae:115.670522,((CPCCuNi_Cryptarchinae:24.656435,CPCCuNi_Cillaeinae:24.656435):13.349086,CPCCuNi_Nitidulinae:38.005521):77.665002):68.547594):5.934889):15.22844,((((CPCChCh_Chrysomelinae:138.942568,CPCChCh_Galerucinae:138.942568):33.497729,(CPCChCh_Lamprosmatinae:154.314869,(CPCChCh_Eumolpinae:125.157134,CPCChCh_Cryptocephalinae:125.157134):29.157735):18.125428):11.28482,((CPCChCh_Donaciinae:132.722165,CPCChCh_Criocerinae:132.722165):26.73755,CPCChCh_Bruchinae:159.459715):24.265402,CPCChCh_Sagrinae:183.725117):7.620446,((CPCChCe_Vesperinae:128.367417,CPCChOr_Aulacoscelidinae:128.367417,CPCChMe_Palophaginae:128.367417,((CPCChMe_Zeugophorinae:100.801976,CPCChCe_Parandrinae:100.801976):14.949735,(CPCChCe_Spondylidinae:100.2655,CPCChOr_Orsodacninae:100.2655):15.486211):12.615706):39.259017,(CPCChCe_Disteniinae:137.092832,(CPCChCe_Necydalinae:124.176387,CPCChCe_Lepturinae:124.176387):12.916445):30.533602):23.719129):14.035882):9.217322,(CPCChCe_Lamiinae:148.267678,(CPCChCe_Prioninae:111.054178,CPCChCe_Cerambycinae:111.054178):37.2135):66.331089):6.274647,((((((CPCCucNe_Rhinorhynchinae:53.351365,(CPCCucAn_Choraginae:32.844648,CPCCucAn_Anthribinae:32.844648):20.506717,CPCCucAn_Urodontinae:53.351365):13.333817,(CPCCucAt_Rhynchitinae:58.889506,CPCCucAt_Attelabinae:58.889506):7.795676):6.838224,CPCCucNe_Doydirhynchinae:73.523406):81.530892,((((CPCCucBr_Cycladinae:85.065205,CPCCucCu_Scolytinae:85.065205):18.571379,CPCCucBr_Brentinae:103.636584):33.903845,((((CPCCucCu_Cossoninae:67.54437,CPCCucCu_Platypodinae:67.54437):27.434326,CPCCucCu_Curculioninae:94.978695):14.252003,CPCCucCu_Dryophthorinae:109.230698):9.460973,CPCCuc_Caridae:118.691671,((CPCCucCu_Brachycerinae:70.356487,(CPCCucIt_Ithyceridae:67.879378,CPCCucBr_Nanophyinae:67.879378):2.477109):9.959317,CPCCucBr_Apioninae:80.315804):38.375866):18.848758):14.005907,(CPCCucBe_Belinae:58.747926,CPCCucBe_Oxycoryninae:58.747926):92.79841):3.507963):32.558311,(CPCChCh_Hispinae:125.677965,CPCChCh_Cassidinae:125.677965):61.934645):12.36795,(CPCCuSi_Silvaninae:143.982597,CPCCuSi_Brontinae:143.982597):55.997963):20.892854):5.496113):10.363488,(((((((CPEBy_Callirhipidae:129.383503,(CPEByPt_Ptilodactylinae:113.416393,CPEByPt_Cladotominae:113.416393):15.96711):15.608274,(CPEByEl_Larainae:95.477934,(CPEBy_Chelonariidae:64.808227,CPEByEl_Elminae:64.808227):30.669708):49.513843):10.674167,(CPEBy_Eulichadidae:115.339965,CPEByPs_Eubrianacinae:115.339965):40.325979):20.186772,(CPEByLi_Limnichinae:137.524766,CPEBy_Dryopidae:137.524766):38.32795):32.166318,((((((CPEElLa_Luciolinae:90.658062,CPEElLa_Ototretinae:90.658062):62.699575,(((((CPEElLy_Erotinae:60.187908,CPEElLy_Lycinae:60.187908):14.238478,CPEElLy_Calochrominae:74.426386):12.725702,CPEElLy_Metriorrhynchinae:87.152088):13.848871,CPEElLy_Ateliinae:101.000959):22.984063,CPEElLy_Leptolycinae:123.985022):29.372615):9.154417,((CPEElCa_Cantharinae:93.801393,CPEElCa_Chauliognathinae:93.801393):23.098502,CPEElCa_Malthininae:116.899894):45.61216):5.747364,(((((CPEElEl_Cardiophorinae:93.656266,CPEEl_Phengodidae:93.656266):21.810527,(CPEElEl_Elaterinae:97.924564,CPEElEl_Thylacosterninae:97.924563):17.54223):10.238421,CPEEl_Rhagophthalmidae:125.705215):14.225275,(CPEElElDe_Denticollis:117.373029,(CPEEl_Drilidae:94.027624,CPEElEl_Agrypninae:94.027624):23.345405):22.557461):13.702054,CPEEl_Omalisidae:153.632544):14.626874):14.124401,(CPEElEu_Eucneminae:142.524507,CPEEl_Throscidae:142.524507):39.859312):17.737037,(((CPEDaDa_Dascillinae:73.111166,CPEDa_Rhipiceridae:73.111166):66.995779,(CPEByBy_Byrrhinae:47.576306,CPEByBy_Syncalyptinae:47.576306):92.530639):28.182904,CPBBo_Nosodendridae:168.289849):31.831007):7.898178):9.665134,((((CPEBuBu_Agrilinae:93.727741,(CPEBuBu_Buprestinae:63.051166,CPEBuBu_Acmaeoderinae:63.051166):30.676575):32.12076,CPEBuBu_Julodinae:125.848501):16.666906,CPEBuBu_Trachyinae:142.515407):32.363743,CPEBy_Heterocerinae:174.87915):42.805018):12.532351,((((CPBBoAn_Dryophilinae:101.631929,(CPBBoBo_Bostrichinae:80.463337,CPBBoBo_Dinoderinae:80.463337):21.168591):24.842792,CPBBoBo_Lyctinae:126.474721):55.193283,((((CPBBoAn_Mesocoelopodinae:99.380145,CPBBoAn_Ptilininae:99.380145):29.36895,(CPBBoAn_Dorcatominae:97.200887,CPBBoAn_Xyletininae:97.200887):31.548207):12.978918,CPBBoAn_Anobiinae:141.728013):35.156093,(CPBBoAn_Gibbiinae:113.666038,CPBBoAn_Ptininae:113.666038):63.218068):4.783899):33.288941,((((CPBBoDe_Dermestinae:114.834904,CPBBoDe_Trinodinae:114.834904):8.215186,CPBBoDe_Megatominae:123.05009):40.140107,CPBBoDe_Attageninae:163.190197):17.512177,CPBBoDe_Orphilinae:180.702374):34.254571):15.259573):6.516497):1.662567,((((((CPScScSc_Dynastinae:92.196954,CPScScSc_Rutelinae:92.196954):36.268849,(CPScScSc_Orphninae:111.525301,CPScScSc_Melolonthinae:111.525301):16.940503):11.657065,CPScScSc_Cetoniinae:140.122869):28.858815,(CPScSc_Glaphyridae:97.662312,CPScScOc_Ochodaeinae:97.662312):71.319373):14.327834,(((CPScScSc_Scarabaeinae:94.772935,CPScSc_Glaresidae:94.772935):35.917334,CPScSc_Hybosoridae:130.690269):25.457974,CPScScSc_Aphodiinae:156.148243):27.161276):8.092642,(((((CPScScLu_Dorcinae:78.958949,CPScScLu_Lucaninae:78.958949):24.683298,(CPScScLu_Aesalinae:89.038679,CPScScLu_Nicaginae:89.038679):14.603568):8.517043,CPScSc_Ceratocanthidae:112.15929):10.882057,CPScScGe_Bolboceratinae:123.041346):14.838795,(CPScSc_Trogidae:121.358845,(CPScScPa_Aulacocyclinae:38.006055,CPScScPa_Passalinae:38.006055):83.35279):16.521297):53.522019):46.993423):1.644736,((CPSHyHy_Hydrophilinae:169.739316,((((CPSHy_Hydrochidae:52.109872,CPSHy_Spercheidae:52.109872):10.881745,CPSHy_Epimetopidae:62.991617):69.479161,CPSHy_Georissidae:132.470778):18.162157,(CPSHyHy_Sphaeridiinae:123.336455,CPSHy_Helophoridae:123.336455):27.29648):19.106381):58.045311,((((((CPSStSt_Staphylininae:135.390953,(CPSStSt_Phloeocharinae:82.742782,CPSStSt_Pseudopsinae:82.742782):52.648171):19.224403,(CPSStSt_Osoriinae:101.74582,CPSStSt_Oxytelinae:101.74582):52.869536):22.879322,(CPSStSt_Paederinae:145.62755,(CPSStSt_Piestinae:131.96982,(CPSStSt_Euaesthetinae:121.602841,(CPSStSt_Oxyporinae:67.061534,CPSStSt_Steninae:67.061534):54.541307):10.366979):13.657731):31.867127):21.172518,(CPSStSt_Tachyporinae:161.296353,CPSStSi_Silphinae:161.296353):37.370842):10.956288,((CPSStSt_Habrocerinae:151.494835,CPSStSt_Micropeplinae:151.494835):32.505434,((CPSStSt_Megalopsidiinae:121.40299,(CPSStSt_Aleocharinae:99.378925,CPSStSt_Proteininae:99.378925):22.024065):47.896732,(CPSStSt_Omalinae:134.689469,CPSStSt_Glypholomatinae:134.689469):34.610253):14.700548):25.623214):5.166687,CPSStSt_Scaphidiinae:214.79017):12.994457):12.255692):3.265094,(((CPSStLe_Leiodinae:120.577236,CPSStLeCh_Nargus:120.577237):8.394557,CPSStLe_Camiarinae:128.971794):9.938103,CPSSt_Agyrtidae:138.909896):104.395517):3.025783,((CPSStPt_Ptilinae:157.375493,(CPSStPt_Acrotrichinae:132.804582,CPSStPt_Cephaloplectinae:132.804582):24.57091):68.352109,CPSStHyHy_Hydraena:225.727601):20.603595):3.10211,(((CPSHi_Sphaeritidae:172.058513,((CPSHiHi_Saprininae:106.873796,(CPSHiHi_Onthophilinae:76.229616,CPSHiHi_Abraeinae:76.229616):30.64418):32.202667,(CPSHiHi_Dendrophilinae:118.885837,((CPSHiHi_Hetaeriinae:51.967131,CPSHiHi_Tribalinae:51.967131):33.487396,CPSHiHi_Histerinae:85.454526):33.431311):20.190626):32.98205):12.786825,CPSHi_Synteliidae:184.845338):43.105106,(CPSStSc_Scydmaeninae:162.85513,CPSStSc_Mastiginae:162.85513):65.095314):21.482862):9.968214,(CPEScCl_Clambinae:191.455566,CPESc_Eucinetidae:191.455566):67.945954):3.974247,CPBDeDe_Laricobiinae:263.375767):3.987135,CPEScSc_Scirtidae:267.362902):9.645983,(((CAC_Elaphrinae:124.300828,((CAC_Brachininae:86.915308,CAC_Siagoninae:86.915308):22.169508,CA_Trachypachidae:109.084816):15.216012):91.427461,((((((CAC_Rhysodinae:133.037496,CAC_Loricerinae:133.037496):13.082057,CAC_Migadopinae:146.119553):8.330687,CAC_Cicindelinae:154.45024):18.122937,(CAC_Paussinae:153.342423,CAC_Harpalinae:153.342423):19.230755):19.397407,(CAC_Carabinae:167.472135,CAC_Trechinae:167.472135):24.49845):11.216713,(CAC_Gehringiinae:181.192936,(CAC_Scaritinae:157.307598,CAC_Omophroninae:157.307598):23.885338):21.994362):12.540991):20.224959,(((CAG_Sphanglerogyrinae:152.898724,CAG_Gyrininae:152.898724):48.517216,(CA_Haliplidae:189.468345,(CAN_Notomicrinae:148.541772,CAN_Noterinae:148.541772):40.926573):11.947595):17.668904,((((CAD_Dytiscinae:115.689459,CAD_Laccophilinae:115.689459):24.827805,CAD_Copelatinae:140.517264):10.429245,((((CA_Amphizoidae:88.751628,CAD_Hydroporinae:88.751628):22.670546,CAD_Agabinae:111.422174):18.039298,CAD_Colymbetinae:129.461473):13.263393,(CAD_Coptotominae:110.539259,CAD_Lancetinae:110.539259):32.185607):8.221643):17.53053,(CA_Paelobiidae:134.07895,CA_Aspidytidae:134.07895):34.398089):50.607804):16.868405):41.055638):7.991114,((CM_Torridincolidae:178.393042,CM_Hydroscaphidae:178.393042):48.462542,(CAr_Cupedidae:173.471283,CM_Sphaeriusidae:173.471283):53.384302):58.144415);

end;
