# Supplementary material for: Herbivory increases diversification across insect clades
Source: Nat Commun. 2015 Sep 24;6:8370. doi: 10.1038/ncomms9370 (PMC4598556; doi:10.1038/ncomms9370)
Supplement: Supplementary Data 5 — Reduced phylogeny of dipteran families used in this study as modified from Wiegmann and colleagues13 [file ncomms9370-s6.docx]

**Supplementary Data 5. Reduced phylogeny of dipteran families used in this study as modified from Wiegmann and colleagues^13^**

#NEXUS

begin taxa;

dimensions ntax=142;

taxlabels

Deuterophlebiidae

Tipulidae

Trichoceridae

Perissomatidae

Canthyloscelididae

Scatopsidae

Axymyiidae

Manotidae

Ditomyiidae

Lygistorrhinidae

Cecidomyiidae

Sciaridae

Mycetophilidae

Bolitophilidae

Keroplatidae

Diadocidiidae

Bibionidae

Pachyneuridae

Anisopodidae

Apystomyiidae

Pipunculidae

Chyromyidae

Opomyzidae

Sepsidae

Acartophthalmidae

Richardiidae

Lonchaeidae

Ulidiidae

Ctenostylidae

Tachiniscidae

Pyrgotidae

Tephritidae

Platystomatidae

Piophilidae

Milichiidae

Chloropidae

Somatiidae

Diopsidae

Marginidae

Nannodastiidae

Canacidae

Inbiomyiidae

Neminidae

Aulacigastridae

Neriidae

Pseudopomyzidae

Micropezidae

Tanypezidae

Strongylophthalmyiidae

Teratomyzidae

Heleomyzidae

Natalimyzidae

Heterocheilidae

Dryomyzidae

Phaeomyiidae

Conopidae

Sciomyzidae

Coelopidae

Helosciomyzidae

Huttoninidae

Helcomyzidae

Celyphidae

Lauxaniidae

Chamaemyiidae

Rhopalomeridae

Fergusoninidae

Asteiidae

Xenasteiidae

Australimyzidae

Clusiidae

Neurochaetidae

Pallopteridae

Psilidae

Syringogastridae

Megamerinidae

Odiniidae

Agromyzidae

Sphaeroceridae

Periscelididae

Carnidae

Ephydridae

Camillidae

Diastatidae

Curtonotidae

Drosophilidae

Braulidae

Chryptochaetidae

Hippoboscidae

Glossinidae

Fanniidae

Muscidae

Anthomyiidae

Scathophagidae

Rhinophoridae

Calliphoridae

Tachinidae

Oestridae

Sarcophagidae

Syrphidae

Lonchopteridae

Ironomyiidae

Phoridae

Platypezidae

Opetiidae

Atelestidae

Dolichopodidae

Hybotidae

Empididae

Asilidae

Mydidae

Apioceridae

Scenopinidae

Therevidae

Evocoidae

Apsilocephalidae

Bombyliidae

Pantophthalmidae

Xylomyidae

Stratiomyidae

Hilarimorphidae

Acroceridae

Nemestrinidae

Xylophagidae

Rhagionidae

Vermileonidae

Pelecorhynchidae

Oreoleptidae

Tabanidae

Athericidae

Dixidae

Corethrellidae

Chaoboridae

Culicidae

Ceratopogonidae

Chironomidae

Thaumaleidae

Simuliidae

Blephariceridae

Tanyderidae

Psychodidae

Ptychopteridae

Nymphomyiidae

;

end;

begin trees;

tree PAUP_1 = [&R] (Deuterophlebiidae:240.0,(((Tipulidae:230.604249,Trichoceridae:230.60425):3.739674,((Perissomatidae:227.234738,((((Canthyloscelididae:137.850797,Scatopsidae:137.850797):70.391972,(Axymyiidae:198.257441,(((Manotidae:132.697137,Ditomyiidae:132.697137):31.725808,((((Lygistorrhinidae:96.232947,Cecidomyiidae:96.232947):21.134398,Sciaridae:117.367344):18.034201,(Mycetophilidae:124.635329,(Bolitophilidae:105.928751,Keroplatidae:105.928751):18.706578):10.766216):8.412535,Diadocidiidae:143.81408):20.608865):15.579041,(Bibionidae:147.393552,Pachyneuridae:147.393552):32.608433):18.255455):9.985329):6.159617,Anisopodidae:214.402387):10.436521,(((Apystomyiidae:167.216603,(((Pipunculidae:89.978303,(((((Chyromyidae:59.278832,(Opomyzidae:54.224391,(Sepsidae:46.561136,Acartophthalmidae:46.561136):7.663255):5.054441):4.294721,((Richardiidae:53.50199,Lonchaeidae:53.50199):7.399771,((Ulidiidae:48.15968,((Ctenostylidae:33.308169,((Tachiniscidae:24.899758,Pyrgotidae:24.899758):5.031203,Tephritidae:29.930961):3.377208):5.011786,Platystomatidae:38.319955):9.839725):7.582843,Piophilidae:55.742523):5.159238):2.671792):5.563333,(((((Milichiidae:43.073477,Chloropidae:43.073477):14.334573,Somatiidae:57.40805):4.33521,(Diopsidae:58.264019,(Marginidae:54.324041,(Nannodastiidae:50.967971,Canacidae:50.967972):3.35607):3.939977):3.479242):2.078619,((Inbiomyiidae:44.957012,Neminidae:44.957012):14.24639,(Aulacigastridae:57.33362,((Neriidae:38.001568,Pseudopomyzidae:38.001568):14.499057,Micropezidae:52.500625):4.832995):1.869782):4.618476):3.466947,((Tanypezidae:48.779912,Strongylophthalmyiidae:48.779912):14.352699,(((Teratomyzidae:54.72233,Heleomyzidae:54.722329):2.68492,(((Natalimyzidae:36.813904,Heterocheilidae:36.813904):6.432522,((Dryomyzidae:37.452227,(Phaeomyiidae:32.037258,(Conopidae:30.006991,Sciomyzidae:30.006991):2.030267):5.414969):2.92542,((Coelopidae:33.505592,Helosciomyzidae:33.505592):3.836897,(Huttoninidae:33.611683,Helcomyzidae:33.611683):3.730806):3.035158):2.868779):4.011463,((Celyphidae:28.174192,Lauxaniidae:28.174192):16.100639,Chamaemyiidae:44.274832):2.983058):10.14936):4.012304,((Rhopalomeridae:53.771237,((Fergusoninidae:43.657096,Asteiidae:43.657096):5.859541,(Xenasteiidae:34.563645,Australimyzidae:34.563645):14.952992):4.2546):4.963055,((Clusiidae:51.488758,(Neurochaetidae:45.521984,Pallopteridae:45.521984):5.966773):3.035497,(Psilidae:49.844784,Syringogastridae:49.844784):4.679471):4.210037):2.685261):1.713059):4.156215):1.84806):3.092983,Megamerinidae:72.229869):2.76924,((Odiniidae:69.852082,((Agromyzidae:64.968344,Sphaeroceridae:64.968345):2.99253,(Periscelididae:61.196636,Carnidae:61.196636):6.764239):1.891207):2.902895,((Ephydridae:63.86003,((Camillidae:51.676736,(Diastatidae:46.957992,Curtonotidae:46.957992):4.718744):5.532881,(Drosophilidae:51.112537,(Braulidae:44.285501,Chryptochaetidae:44.285501):6.827035):6.09708):6.650414):5.111367,((Hippoboscidae:44.422604,Glossinidae:44.422605):13.791047,(Fanniidae:55.28718,(Muscidae:50.6634,((Anthomyiidae:34.846597,Scathophagidae:34.846597):9.15434,((Rhinophoridae:32.852768,(Calliphoridae:28.744622,Tachinidae:28.744622):4.108146):3.948964,(Oestridae:31.170895,Sarcophagidae:31.170895):5.630837):7.199206):6.662462):4.62378):2.926472):10.757745):3.783579):2.244132):14.979194):12.313158,Syrphidae:102.29146):49.929565,(Lonchopteridae:131.475522,(((Ironomyiidae:94.361368,Phoridae:94.361368):16.446165,Platypezidae:110.807532):13.523102,Opetiidae:124.330634):7.144888):20.745504):14.995577):16.42191,(Atelestidae:157.090399,(Dolichopodidae:124.768098,(Hybotidae:115.922499,Empididae:115.922498):8.845599):32.322301):26.548115):16.493359,(((((Asilidae:133.274832,(Mydidae:118.779037,Apioceridae:118.779037):14.495795):10.670332,((Scenopinidae:117.426422,Therevidae:117.426422):16.312405,(Evocoidae:122.902416,Apsilocephalidae:122.902416):10.836411):10.206336):25.532832,Bombyliidae:169.477996):15.67178,((Pantophthalmidae:155.00151,(Xylomyidae:138.214319,Stratiomyidae:138.214318):16.787191):22.191922,(Hilarimorphidae:161.597273,Acroceridae:161.597273):15.596159):7.956343):8.324119,((Nemestrinidae:164.465944,Xylophagidae:164.465944):19.895071,((Rhagionidae:153.003893,Vermileonidae:153.003893):28.274477,(Pelecorhynchidae:154.753349,(Oreoleptidae:144.123412,(Tabanidae:130.001669,Athericidae:130.001669):14.121743):10.629937):26.52502):3.082646):9.112879):6.657979):24.707035):2.395831):5.133129,((((Dixidae:213.854302,((Corethrellidae:143.570835,Chaoboridae:143.570835):38.165396,Culicidae:181.736231):32.118072):7.807847,((Ceratopogonidae:210.000612,Chironomidae:210.000612):9.246357,(Thaumaleidae:163.055769,Simuliidae:163.055769):56.1912):2.41518):7.919507,(Blephariceridae:221.362627,(Tanyderidae:180.001387,Psychodidae:180.001388):41.36124):8.219029):1.515416,Ptychopteridae:231.097072):1.270794):1.976057):2.1241,Nymphomyiidae:236.468023):3.531977);

end;
