# Supplementary material for: Herbivory increases diversification across insect clades
Source: Nat Commun. 2015 Sep 24;6:8370. doi: 10.1038/ncomms9370 (PMC4598556; doi:10.1038/ncomms9370)
Supplement: Supplementary Data 6 — Reduced phylogeny of hemipteran families used in this study as modified from Rainford and colleagues12 [file ncomms9370-s7.docx]

**Supplementary Data 6. Reduced phylogeny of hemipteran families used in this study as modified from Rainford and colleagues^12^**

#NEXUS

begin taxa;

dimensions ntax=93;

taxlabels

He_Psylloi

He_Aleyrod

He_Coccoid

He_Phyllox

He_Aphidoi

He_Myerslo

He_Cicadel

He_Membrac

He_Aetalio

He_Cicadid

He_Machaer

He_Cercopi

He_Aphroph

He_Clastop

He_Epipygi

He_Derbida

He_Meenopl

He_Flatida

He_Delphac

He_Cixiida

He_Achilid

He_Tropidu

He_Fulgori

He_Tettigo

He_Dictyop

He_Issidae

He_Nogodin

He_Eurybra

He_Ricanni

He_Lophopi

He_Achilix

He_Calisce

He_Pelorid

He_Naucori

He_Notonec

He_Apheloc

He_Ochteri

He_Gelasto

He_Belosto

He_Nepidae

He_Corixid

He_Enicoce

He_Schizop

He_Dipsoco

He_Hydrome

He_Veliida

He_Gerrida

He_Hermato

He_Mesovel

He_Macrove

He_Paraphy

He_Hebrida

He_Pleidae

He_Saldida

He_Leptopo

He_Miridae

He_Tingida

He_Thaumas

He_Plokiop

He_Lycetoc

He_Cimicid

He_Anthoco

He_Joppeic

He_Microph

He_Phymati

He_Nabidae

He_Velocip

He_Reduvii

He_Berytid

He_Aradida

He_Canopid

He_Cydnida

He_Dinidor

He_Tessara

He_Platasp

He_Pentato

He_Phloeid

He_Lestoni

He_Acantho

He_Scutell

He_Idiosto

He_Hyoceph

He_Alydida

He_Coreida

He_Rhopali

He_Stenoce

He_Largida

He_Pyrrhoc

He_Piesmat

He_Lygaeio

He_Malcida

He_Termita

He_Colobat

;

end;

begin trees;

tree PAUP_1 = [&R] (((He_Psylloi:321.3974,He_Aleyrod:321.3974):29.55692,(He_Coccoid:286.4426,(He_Phyllox:87.13851,He_Aphidoi:87.13851):199.3041):64.5117):40.77385,(((He_Myerslo:287.1723,((He_Cicadel:170.6714,(He_Membrac:127.2758,He_Aetalio:127.2758):43.39561):90.14826,(He_Cicadid:224.8581,(He_Machaer:162.3958,(He_Cercopi:128.2812,(He_Aphroph:101.9798,(He_Clastop:69.14481,He_Epipygi:69.14481):32.83497):26.30146):34.11458):62.46226):35.96163):26.35257):57.31865,(He_Derbida:304.7509,((He_Meenopl:182.2549,He_Flatida:182.2549):111.091,((He_Delphac:190.6426,He_Cixiida:190.6426):70.21615,((He_Achilid:176.3839,He_Tropidu:176.3839):45.85781,((He_Fulgori:154.2368,(He_Tettigo:96.17745,He_Dictyop:96.17745):58.05939):51.89819,((He_Issidae:88.13178,He_Nogodin:88.13178):100.0794,(He_Eurybra:157.4173,(He_Ricanni:141.7221,(He_Lophopi:108.6603,(He_Achilix:81.15765,He_Calisce:81.15765):27.50262):33.06186):15.6952):30.7938):17.92389):16.10664):38.61707):32.48713):11.40499):39.74006):21.67247,(He_Pelorid:336.1533,((((He_Naucori:192.3887,(He_Notonec:129.4199,He_Apheloc:129.4199):62.96871):93.45146,((He_Ochteri:179.2143,He_Gelasto:179.2143):85.73937,(He_Belosto:239.4364,He_Nepidae:239.4364):25.51726):20.88647):18.72672,(He_Corixid:263.6769,(He_Enicoce:250.2628,((He_Schizop:160.0609,He_Dipsoco:160.0609):67.8111,((He_Hydrome:159.8218,(He_Veliida:118.0078,He_Gerrida:118.0078):41.81391):34.11992,((He_Hermato:122.3117,He_Mesovel:122.3117):39.21565,(He_Macrove:135.2462,(He_Paraphy:101.3662,He_Hebrida:101.3662):33.88001):26.2811):32.41438):33.93035):22.39075):13.41411):40.88993):17.33273,((He_Pleidae:253.9732,(He_Saldida:172.733,He_Leptopo:172.733):81.24019):51.11897,((((He_Miridae:140.7011,He_Tingida:140.7011):84.61733,(He_Thaumas:204.6459,(He_Plokiop:177.0309,(He_Lycetoc:147.9228,(He_Cimicid:125.2985,He_Anthoco:125.2985):22.62433):29.10812):27.61497):20.67249):29.79845,(He_Joppeic:231.8878,(He_Microph:212.2647,(He_Phymati:178.6019,((He_Nabidae:103.2646,He_Velocip:103.2646):48.96412,(He_Reduvii:95.246,He_Berytid:95.246):56.98273):26.37314):33.66284):19.62306):23.22907):37.08026,(He_Aradida:272.6347,(((He_Canopid:131.825,He_Cydnida:131.825):78.03549,((He_Dinidor:123.5364,He_Tessara:123.5364):65.22941,((He_Platasp:124.1235,He_Pentato:124.1235):37.92377,(He_Phloeid:141.8907,(He_Lestoni:118.2775,(He_Acantho:72.53056,He_Scutell:72.53056):45.74696):23.61322):20.15649):26.7186):21.09462):51.99564,((He_Idiosto:178.6455,He_Hyoceph:178.6455):68.97378,((He_Alydida:222.7753,(He_Coreida:213.9783,He_Rhopali:213.9783):8.797014):11.33559,((He_Stenoce:150.5309,(He_Largida:104.7187,He_Pyrrhoc:104.7187):45.81219):67.68101,(He_Piesmat:185.3944,(He_Lygaeio:157.547,(He_Malcida:134.162,(He_Termita:94.7507,He_Colobat:94.7507):39.41131):23.38501):27.84734):32.81751):15.89902):13.50839):14.23681):10.77863):19.56238):12.8951):16.80735):14.25379):30.01004):25.56479);

end;
