# Supplementary material for: Herbivory increases diversification across insect clades
Source: Nat Commun. 2015 Sep 24;6:8370. doi: 10.1038/ncomms9370 (PMC4598556; doi:10.1038/ncomms9370)
Supplement: Supplementary Data 7 — Reduced phylogeny of hymenopteran families used in this study as modified from Rainford and colleagues12 [file ncomms9370-s8.docx]

**Supplementary Data 7. Reduced phylogeny of hymenopteran families used in this study** **as modified** **from Rainford and colleagues^12^**

#NEXUS

begin taxa;

dimensions ntax=77;

taxlabels

Hy_Xyelida

Hy_Blastic

Hy_Pergida

Hy_Tenthre

Hy_Argidae

Hy_Cimbici

Hy_Diprion

Hy_Pamphil

Hy_Megalod

Hy_Xiphydr

Hy_Cephida

Hy_Siricid

Hy_Anaxeli

Hy_Orussid

Hy_Stephan

Hy_Megalyr

Hy_Scelion

Hy_Platyga

Hy_Helorid

Hy_Maaming

Hy_Mymaron

Hy_Diaprii

Hy_Monomac

Hy_Proctot

Hy_Vanhorn

Hy_Pelecin

Hy_Roprion

Hy_Figitid

Hy_Cynipid

Hy_Liopter

Hy_Ibaliid

Hy_Euchari

Hy_Encyrti

Hy_Agaonid

Hy_Mymarid

Hy_Eulophi

Hy_Pteroma

Hy_Torymid

Hy_Perilam

Hy_Aphelin

Hy_Tetraca

Hy_Rotoiti

Hy_Trichog

Hy_Eupelmi

Hy_Eurytom

Hy_Chalcid

Hy_Trigona

Hy_Braconi

Hy_Ichneum

Hy_Ceraphr

Hy_Megaspi

Hy_Gasteru

Hy_Evaniid

Hy_Aulacid

Hy_Sierolo

Hy_Mutilid

Hy_Sapygid

Hy_Scoliid

Hy_Pompili

Hy_Ampulic

Hy_Bradyno

Hy_Formici

Hy_Chrysid

Hy_Scoleby

Hy_Plumari

Hy_Bethyli

Hy_Crabron

Hy_Tiphiid

Hy_Vespida

Hy_Sphecid

Hy_Andreni

Hy_Halicti

Hy_Stenotr

Hy_Colleti

Hy_Melitti

Hy_Apidae

Hy_Megachi

;

end;

begin trees;

tree PAUP_1 = [&R] (Hy_Xyelida:257.2901,((Hy_Blastic:187.4191,(Hy_Pergida:148.5929,((Hy_Tenthre:76.68761,Hy_Argidae:76.68761):40.83015,(Hy_Cimbici:70.70218,Hy_Diprion:70.70218):46.81559):31.07517):38.82619):64.07693,((Hy_Pamphil:186.488,Hy_Megalod:186.488):60.17937,(Hy_Xiphydr:239.9396,(Hy_Cephida:235.9364,((Hy_Siricid:136.7405,Hy_Anaxeli:136.7405):95.3333,((Hy_Orussid:147.1096,Hy_Stephan:147.1096):80.23359,(((Hy_Megalyr:177.379,(Hy_Scelion:110.3334,Hy_Platyga:110.3334):67.04556):40.49218,((Hy_Helorid:176.2314,(Hy_Maaming:121.9638,Hy_Mymaron:121.9638):54.26763):32.8927,(((Hy_Diaprii:103.8914,Hy_Monomac:103.8914):89.65138,((Hy_Proctot:107.6689,Hy_Vanhorn:107.6689):73.40022,((Hy_Pelecin:158.8354,Hy_Roprion:158.8354):10.43906,((Hy_Figitid:103.4603,Hy_Cynipid:103.4603):27.46697,(Hy_Liopter:79.20717,Hy_Ibaliid:79.20717):51.72009):38.34721):11.79467):12.4736):9.560329,((Hy_Euchari:143.7983,(Hy_Encyrti:123.1046,(Hy_Agaonid:89.35121,Hy_Mymarid:89.35121):33.75336):20.69375):31.11842,(((Hy_Eulophi:48.57657,Hy_Pteroma:48.57657):78.01182,(Hy_Torymid:96.63939,(Hy_Perilam:66.31041,Hy_Aphelin:66.31041):30.32898):29.94901):21.94406,((Hy_Tetraca:83.41545,Hy_Rotoiti:83.41545):36.83201,(Hy_Trichog:97.5552,(Hy_Eupelmi:83.04234,(Hy_Eurytom:45.76513,Hy_Chalcid:45.76513):37.27721):14.51285):22.69226):28.28499):26.38428):28.18633):6.021075):8.747003):5.676466,(((Hy_Trigona:131.1363,Hy_Braconi:131.1363):58.90993,(Hy_Ichneum:136.9222,(Hy_Ceraphr:75.91769,Hy_Megaspi:75.91769):61.00448):53.12405):27.61778,((Hy_Gasteru:149.6837,(Hy_Evaniid:110.2558,Hy_Aulacid:110.2558):39.42793):63.12467,(((Hy_Sierolo:94.99117,Hy_Mutilid:94.99117):82.89446,(Hy_Sapygid:149.2829,(Hy_Scoliid:122.8755,Hy_Pompili:122.8755):26.40744):28.6027):29.23741,(((Hy_Ampulic:145.64,(Hy_Bradyno:108.6693,Hy_Formici:108.6693):36.97077):34.75056,(Hy_Chrysid:163.1938,(Hy_Scoleby:140.9128,(Hy_Plumari:123.6116,Hy_Bethyli:123.6116):17.3012):22.28105):17.19677):14.39109,((Hy_Crabron:158.4217,(Hy_Tiphiid:116.3308,Hy_Vespida:116.3308):42.09097):18.62656,(Hy_Sphecid:154.1938,(Hy_Andreni:130.6676,(Hy_Halicti:112.7857,((Hy_Stenotr:52.19141,Hy_Colleti:52.19141):45.31524,(Hy_Melitti:69.51568,(Hy_Apidae:37.56132,Hy_Megachi:37.56132):31.95436):27.99096):15.27909):17.88186):23.52617):22.85454):17.73338):12.34137):5.685351):4.855597):5.88362):3.79559):4.730583):3.862596):4.003269):6.727747):4.828653):5.794016);

end;
