# Supplementary material for: Herbivory increases diversification across insect clades
Source: Nat Commun. 2015 Sep 24;6:8370. doi: 10.1038/ncomms9370 (PMC4598556; doi:10.1038/ncomms9370)
Supplement: Supplementary Data 8 — Reduced phylogeny of orthopteran families used in this study as modified from Rainford and colleagues12 [file ncomms9370-s9.docx]

**Supplementary Data 8. Reduced phylogeny of orthopteran families used in this study** **as modified from Rainford and colleagues^12^**

#NEXUS

begin taxa;

dimensions ntax=26;

taxlabels

Or_E_Grylli

Or_E_Myrme

Or_E_Gryllot

Or_E_Steno

Or_E_Gryllac

Or_E_Anost

Or_E_Rhaph

Or_E_Tetti

Or_E_Proph

Or_C_Cylin

Or_C_Trida

Or_C_Theri

Or_C_Tetri

Or_C_Eusch

Or_C_Eumas

Or_C_Episa

Or_C_Choro

Or_C_Prosc

Or_C_Tanao

Or_C_Pneum

Or_C_Trigo

Or_C_Pyrgo

Or_C_Pamph

Or_C_Lentu

Or_C_Acrid

Or_C_Romal

;

end;

begin trees;

tree PAUP_1 = [&R] (((Or_E_Grylli:195.1914,(Or_E_Myrme:129.7351,Or_E_Gryllot:129.7351):65.45628):40.45091,((Or_E_Steno:129.637,(Or_E_Gryllac:88.91178,Or_E_Anost:88.91178):40.72524):31.26344,(Or_E_Rhaph:118.8688,(Or_E_Tetti:77.82393,Or_E_Proph:77.82393):41.04488):42.03165):74.7418):73.23417,((Or_C_Cylin:211.5551,Or_C_Trida:211.55509):59.52725,((Or_C_Theri:117.4836,Or_C_Tetri:117.4836):93.25394,((Or_C_Eusch:128.4138,(Or_C_Eumas:94.63456,(Or_C_Episa:63.327,Or_C_Choro:63.327):31.30756):33.77922):36.48433,(Or_C_Prosc:137.6494,((Or_C_Tanao:71.80931,Or_C_Pneum:71.80931):54.63257,(Or_C_Trigo:110.7068,(Or_C_Pyrgo:94.4352,(Or_C_Pamph:76.36786,(Or_C_Lentu:56.83744,(Or_C_Acrid:43.17991,Or_C_Romal:43.17991):13.65753):19.53043):18.06734):16.2716):15.73508):11.20753):27.2487):45.83947):60.34476):37.7941);

end;
